# Supplementary material for: A retrieval-augmented knowledge mining method with deep thinking LLMs for biomedical research and clinical support
Source: Gigascience. 2025 Sep 17;14:giaf109. doi: 10.1093/gigascience/giaf109 (PMC12448786; doi:10.1093/gigascience/giaf109)
Supplement: giaf109_Supporting_Information [file giaf109_supporting_information.pdf]

A Retrieval-Augmented Knowledge Mining Method with Deep Thinking LLMs for Biomedical Research and Clinical Support

Yichun Feng[1,2,3], Jiawei Wang[4], Ruikun He[5], Lu Zhou[3*], Yixue Li[2,3,6,7,8*]

[1]School of Advanced Interdisciplinary Sciences, University of Chinese Academy of Sciences, 380, Huaibei Town, Huairou District, Beijing, 100049, Beijing, China.
[2]Key Laboratory of Systems Health Science of Zhejiang Province, School of Life Science, Hangzhou Institute for Advanced Study, University of Chinese Academy of Sciences, No. 1, Xiangshan Zhinuo, Xihu District, Hangzhou, 310024, Zhejiang, China.
[3]Guangzhou National Laboratory, No. 9 XingDaoHuanBei Road, Guangzhou, 510005, Guangdong, China.
[4]Department of EEIS, University of Science and Technology of China, No. 443, Huangshan Road, Shushan District, Hefei, 230026, Anhui, China.
[5]BYHEALTH Institute of Nutrition & Health, Guangzhou, 510663, Guangdong, China.
[6]GZMU-GIBH Joint School of Life Sciences, The Guangdong-Hong Kong-Macau Joint Laboratory for Cell Fate Regulation and Diseases, Guangzhou Medical University, Guangzhou, 511436, Guangdong, China.
[7]School of Life Sciences and Biotechnology, Shanghai Jiao Tong University, Shanghai, 200240, Shanghai, China.
[8]Shanghai Institute of Nutrition and Health, Chinese Academy of Sciences, Shanghai, 200030, Shanghai, China.

*Corresponding author(s). E-mail(s): zhou_lu@gzlab.ac.cn; li_yixue@gzlab.ac.cn;
Contributing authors: fengyichun22@mails.ucas.ac.cn; wangjiawei@mail.ustc.edu.cn; herk@by-health.com;

1 Supporting Information

1.1 Evaluation Criteria

For the BioCDQA dataset, we use custom evaluation metrics as described below.
Document Retrieval Performance Evaluation
we employ several key metrics: Mean Precision assesses the proportion of relevant documents among retrieved results, while Mean Recall measures the fraction of relevant documents successfully retrieved. Mean F-measure provides a balanced evaluation of precision and recall. Together, these metrics offer a comprehensive evaluation of retrieval accuracy and completeness.
Answer Accuracy Evaluation
To evaluate the accuracy and quality of the generated answers, we rely on a single key metric: evaluators based on GPT-4. This metric involves assigning scores on a five-point scale, where a score of 5 is given only when the generated answer fully addresses the question, is semantically identical to the reference answer, and contains no irrelevant or additional information. This stringent evaluation criterion ensures that both semantic accuracy and precision are emphasized. The detailed GPT-4-based Evaluation Prompt is presented in Figure S1. We chose to focus exclusively on this evaluation approach because our dataset primarily consists of summary-type questions. Due to the nature of summary generation, the answers produced by large models may vary in format, making it difficult to apply traditional metrics such as Precision, Recall, and F1-score in a consistent and meaningful way. Evaluators based on GPT-4 allow us to assess the quality of the generated answers holistically, accounting for both the semantic content and the overall structure of the response. This method provides a comprehensive evaluation framework, particularly suitable for tasks where the format and phrasing of the answer can vary, but the underlying accuracy and relevance remain the primary considerations.

GPT4-based Evaluation Prompt

You need to score the answers generated by the LLM based on the question and the standard answer. Use a scale from 0 to 5, where:
5 points: The LLM's answer fully addresses the question, covers all core elements of the standard answer, and conveys the same meaning without any irrelevant information.
4 points: The LLM's answer addresses the question well, covers most core elements of the standard answer, but has minor deviations, omissions, or includes a small amount of irrelevant information.
3 points: The LLM's answer addresses the question but has significant deviations or omissions from the core elements of the standard answer. It also contains noticeable irrelevant information that affects clarity.
2 points: The LLM's answer partially addresses the question and has major deviations or omissions from the core elements of the standard answer. It includes a substantial amount of irrelevant information.
1 point: The LLM's answer barely addresses the question and has severe deviations or omissions from the core elements of the standard answer. It includes misleading or irrelevant information.
0 points: The LLM's answer fails to address the question or conveys a meaning completely opposite to the standard answer. It may also be entirely irrelevant or incomprehensible.
Note: Only the numerical part of the score needs to be output.

Fig. S1 Prompt for GPT-4-based evaluation.

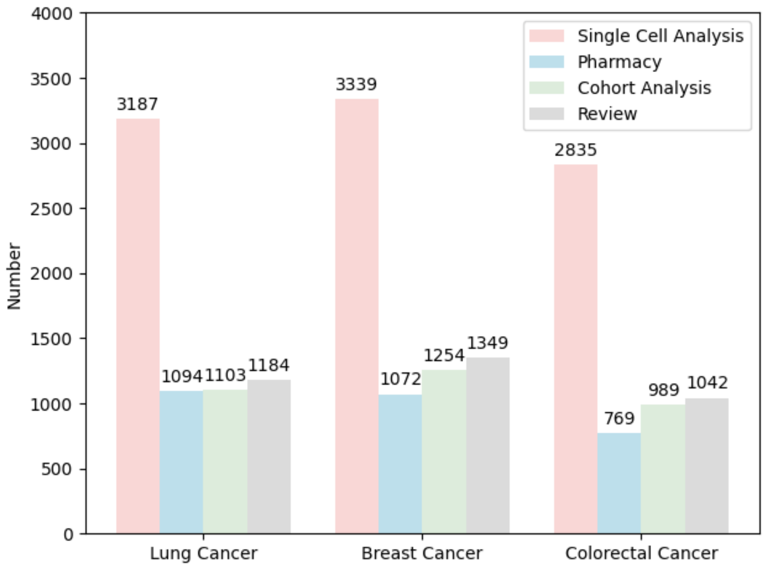

Fig. S2 The number of papers in the dataset. The core themes are Lung Cancer, Breast Cancer, and Colorectal Cancer, and the four analytical methods are single-cell analysis, pharmacology, cohort analysis, and review articles.

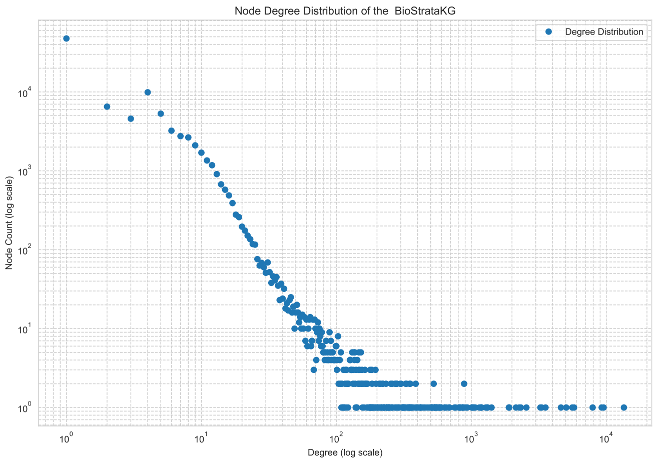

Fig. S3 Node Degree Distribution of the BioStrataKG. The plot shows the distribution of node degrees on a log-log scale.

Triplet Extraction Prompt

Please extract the conclusion triples (entity, relation, entity) from the abstract. The extracted triples must meet the following conditions:
1. (Drug, relation, Drug) with relations: Synergistic Effect, Antagonistic Effect, Metabolic Interaction, Combination Therapy, Alternative Therapy;
2. (Disease, relation, Biomarker) with relations: Diagnosis, Prognosis, Prediction, Therapeutic Monitoring;
3. (Gene, relation, Disease) with relations: Pathogenic Mutation, Susceptibility Variant, Gene Expression, Polygenic Risk;
4. (Drug, relation, Disease) with relations: Disease Indication, Contraindication, Disease Pathology;
5. (Gene, relation, Gene) with relations: Regulation, Interaction, Metabolic Pathway, Co-expression, Inhibition;
6. (Drug, relation, Protein) with relations: Inhibition Target, Promotion Target, Metabolism.
Entities can have the following attributes:
- Drug Attributes: Name, Target, Category, Pathway, Disease Indication, Contraindication, Side Effect, Clinical Trial, Approval.
- Gene Attributes: Name, Symbol, Location, Function, Related Diseases, Mutation Type, Expression Pattern, Regulators, Interactions.
- Protein Attributes: Name, Symbol, Function, Structure, Related Gene, Interactions, Modifications, Localization, Expression Pattern.
- Disease Attributes: Name, Category, Symptoms, Causes, Pathology, Diagnostic Methods, Treatments, Prognosis, Related Genes, Related Drugs.

Only extract the triples that meet the above conditions.
Please provide the triples in the following JSON format:

```
{
 "triples": [
  {
   "entity1": {
    "name": "Your entity name here",
    "type": "Your entity type here",
    "attributes": {
     "attribute1": "value1",
     "attribute2": "value2"
    }
   },
   "relation": "Your relation here",
   "entity2": {
    "name": "Your entity name here",
    "type": "Your entity type here",
    "attributes": {
     "attribute1": "value1",
     "attribute2": "value2"
    }
   }
  }
 ]
}
```

Fig. S4 Prompt for triplet extraction.

Extraction of Paper Information Prompt

Summarize the following parts of the research paper:
1. Basic Research Methodology: Describe the fundamental methodology commonly used in the biomedical field, which serves as the basis for many studies.
2. Innovative Research Methodology: Highlight the unique methodology proposed by this paper that distinguishes it from other studies.
3. Research Domain: The general areas of study within the biomedical field covered in this paper.
4. Datasets Used: The names of the datasets utilized.
5. Dataset Characteristics: Specific features of the datasets used.
Please provide the response in the following JSON format:

```
{
  "basic_research_methodology": "",
  "innovative_research_methodology": "",
  "research_domain": "",
  "datasets_used": "",
  "dataset characteristics": "datasetA: XXX. "
}
```

Fig. S5 Prompt for extraction of paper information.

Dataset Inspection Prompt

You will receive multiple documents from different papers, each containing information relevant to the question. Your task is to identify the passage(s) in each paper that answer the question, and return the exact sentence or passage from the paper. Each paper is guaranteed to contain at least one relevant passage that answers the question, and there may be multiple passages. Please provide the response in the following JSON format:

```
{
 "papers": [
  {
   "paperid": "Paper ID 1",
   "passages": [
    "The exact sentence or passage from Paper ID 1.",
    "Another sentence or passage from Paper ID 1."
   ]
  },
  {
   "paperid": "Paper ID 2",
   "passages": [
    "The exact sentence or passage from Paper ID 2.",
    "Another sentence or passage from Paper ID 2."
   ]
  }
 ]
}
```

Fig. S6 Prompt for dataset inspection.

Pre-Retrieval Reasoning Prompt

Based on the given question, help me identify the key terms and actions in the question.
1. Key terms: These are the entities, subjects, or objects related to the question.
2. Actions: These are the tasks or actions the question focuses on.
3. Generate related synonyms based on key terms and actions to expand the query vocabulary.
4. Additionally, based on the question, generate a plausible virtual answer that could hypothetically address the question, even if it is not grounded in actual data.
Please provide the response in the following JSON format:

```
{
  "key_terms": ["key_term_1","key_term_2"],
  "actions": ["action_1","action_2"],
  "key_terms synonyms": {
    "key_term_1": ["",""],
    "key_term_2": ["",""]
  },
  "actions synonyms": {
    "action_1": ["",""],
    "action_2": ["",""]
  },
  "virtual_answer": "Your generated virtual answer here."
}
```

Fig. S7 Prompt for Pre-Retrieval Reasoning.
