# Supplementary material for: A retrieval-augmented knowledge mining method with deep thinking LLMs for biomedical research and clinical support
Source: Gigascience. 2025 Sep 17;14:giaf109. doi: 10.1093/gigascience/giaf109 (PMC12448786; doi:10.1093/gigascience/giaf109)
Supplement: giaf109_GIGA-D-25-00234_Revision_1 [file giaf109_giga-d-25-00234_revision_1.pdf]

GigaScience

A Retrieval-Augmented Knowledge Mining Method with Deep Thinking LLMs for Biomedical Research and Clinical Support

--Manuscript Draft--

**Manuscript Number:**	GIGA-D-25-00234R1
**Full Title:**	A Retrieval-Augmented Knowledge Mining Method with Deep Thinking LLMs for Biomedical Research and Clinical Support
**Article Type:**	Research
**Funding Information:**	National Key R&D Program (No. 2022YFF1202101, 2023YFF1204701) — Ph.D. Yixue Li<br>the CAS Research Fund (No. XDB38050200) — Ph.D. Yixue Li<br>the Self-supporting Program of Guangzhou National Laboratory (No. SRPG22007) — Ph.D. Yixue Li<br>the Startup Program of Guangzhou National Laboratory (No. YW-YFYJ0101) — Ph.D. Yixue Li

Abstract:

Background. Knowledge graphs and large language models (LLMs) are key tools for biomedical knowledge integration and reasoning, facilitating structured organization of scientific articles and discovery of complex semantic relationships. However, current methods face challenges: knowledge graph construction is limited by complex terminology, data heterogeneity, and rapid knowledge evolution, while LLMs show limitations in retrieval and reasoning, making it difficult to uncover cross-document associations and reasoning pathways.

Results. We propose a pipeline that uses LLMs to construct a biomedical knowledge graph (BioStrataKG) from large-scale articles and builds a cross-document question-answering dataset (BioCDQA) to evaluate latent knowledge retrieval and multi-hop reasoning. We then introduce Integrated and Progressive Retrieval-Augmented Reasoning (IP-RAR) to enhance retrieval accuracy and knowledge reasoning. IP-RAR maximizes information recall through Integrated Reasoning-based Retrieval and refines knowledge via Progressive Reasoning-based Generation, using self-reflection to achieve deep thinking and precise contextual understanding. Experiments show that IP-RAR improves document retrieval F1 score by 20% and answer generation accuracy by 25% over existing methods.

Conclusions. The IP-RAR helps doctors efficiently integrate treatment evidence to inform the development of personalized medication plans and enables researchers to analyze advancements and research gaps, accelerating the hypothesis generation phase of scientific discovery and decision-making.

**Corresponding Author:**	Yichun Feng<br>University of the Chinese Academy of Sciences<br>Hangzhou, CHINA
**Corresponding Author Secondary Information:**	
**Corresponding Author's Institution:**	University of the Chinese Academy of Sciences
**Corresponding Author's Secondary Institution:**	
**First Author:**	Yichun Feng
**First Author Secondary Information:**	
**Order of Authors:**	Yichun Feng
	Jiawei Wang
	Ruikun He

Powered by Editorial Manager® and ProduXion Manager® from Aries Systems Corporation

	Lu Zhou
	Yixue Li
**Order of Authors Secondary Information:**	
**Response to Reviewers:**	Revision Notes for GIGA-D-25-00234<br><br>The authors would like to thank editor and reviewers for their constructive comments and suggestions on our submission. We have addressed the comments and modified the manuscript accordingly. In this revised version, changes to our manuscript within the document have been highlighted by using blue colored text. Please refer to the file “Revision_highlight_giga_234.pdf” in the supplementary materials. Point-by-point responses to the editor and reviewers are listed below.<br><br>Response to EIC:<br>#Q1:The introductory assertions (e.g., supporting drug discovery) require supporting results or concrete examples.<br><br># Response: Thanks for your suggestion. We have performed a systematic revision of the language throughout the entire manuscript. In multiple sections, including the Abstract, Key Points, Introduction (lines 126 - lines 130), Figure Captions, and Discussion (lines 881 - lines 884), we have replaced overly broad verbs and nouns with more precise phrasing. For example, "drug repurposing" has been revised to "generating hypotheses for drug repurposing," and "accelerating scientific discovery" has been refined to "accelerating the hypothesis generation phase of scientific discovery." Through these changes, we have clearly defined the tool's role as a powerful hypothesis generation and decision support tool, rather than an end-to-end discovery platform.<br><br>#Q2:New tools must obtain RRIDs (via bio.tools/SciCrunch), and workflows require DOI registration on workflowhub.eu<br><br># Response: Thanks for your suggestion. We fully agree on the importance of these standard registrations for improving the discoverability and reproducibility of our work. We have already included our registered RRID (SCR_027068) in the "Availability of Source Code and Requirements" section of the manuscript. Furthermore, we have successfully registered our computational workflow on WorkflowHub and add the citable DOI (https://doi.org/10.48546/WORKFLOWHUB.WORKFLOW.1744.1) to this section as well (lines 909- lines 910).<br><br>Response to Reviewer #1:<br>#Q1: Paper collection method is not clear. PubMed has PubMed Central Open Access Subset FTP Bulk Download, which offers XML files in two distinct groups: commercial use and non-commercial use only. The paper collection needs to be clarified further why the PDFs were downloaded instead of XML files. Converting PDFs to Markdown format can introduce noises and the choice of PDFs is less appropriate as there were no usage of Figures or Tables. Also, how the PDFs were converted to markdown is unclear.<br><br># Response: Thanks for your suggestion.<br>(1)We initially considered using the XML files available from open-access repositories. However, we discovered that for a significant portion of the literature, the XML files contained only metadata and abstracts, with the full text being either missing or improperly formatted. As a key objective of our study is to perform full-text knowledge mining, relying solely on XMLs would have been insufficient. The PDF format was therefore essential to ensure we obtained the most complete information, including the detailed discourse within the body of the papers. To leverage the best of both, we adopted a hybrid approach: we preserved the structured metadata and abstracts from the XML files and integrated them with the complete full-text content extracted from the corresponding PDFs (lines 171 - lines 179).<br>(2)For the conversion, we utilized the marker toolkit, which is cited in our manuscript (references [25, 26]). We specifically chose marker because it excels at not only extracting text but also preserving the logical structure of the document (e.g., headings, lists, and paragraphs). We ensure that the revised Methods section clearly states the

Powered by Editorial Manager® and ProduXion Manager® from Aries Systems Corporation

tool used(lines 179 - lines 181).

#Q2: No comparison of LLMs in constructing the KG and QA. Authors just used GPT4o-mini without comparing the performance of this model with other models to support this model selection for both the knowledge graph construction and QA dataset generation. This is important to ensure the quality of the knowledge graph, BioStrataKG, and the dataset, BioCDQA.

Response: Thanks for your suggestion. We ensured quality through a multi-faceted approach. Our model selection was based on three key considerations. First, for practical reasons, GPT-4o mini offered the best balance of performance and cost-effectiveness for our large-scale task. Second, there was a strong technical fit: our "triplet extraction" is a highly structured task with predefined entity and relation types, which aligns perfectly with the strong instruction-following and contextual understanding capabilities of the GPT-4 series models. Third, to quantitatively validate this choice, we conducted a targeted evaluation on a 20-paper sample and found that GPT-4o mini achieved an extraction accuracy of 88.5%. This high accuracy is further supported by our process and product validation measures. Our construction process has built-in quality controls, such as a two-stage entity normalization process that requires alignment with authoritative databases (MeSH and UniProt) and secondary LLM confirmation, which inherently prevents a large number of errors. Finally, we conducted a functional validation via our downstream task. The successful creation of the BioCDQA dataset, which itself underwent rigorous manual curation (lines 458 - lines 475) (filtering from 5,000+ to 1,183 pairs), would have been infeasible if the underlying knowledge graph—the final product—were of poor quality.

#Q3: Limited novelty in the Progressive Reasoning-based Generation It is very similar to Self-Refine: Iterative Refinement with Self-Feedback (NeurIPS 2023). Especially the self-reflective evaluation is just another way of using self-refine.

Response: Thanks for your suggestion. While both approaches leverage a "self-reflection" loop, they are designed for fundamentally different purposes, which is where our novelty lies. Self-Refine's purpose is to iteratively improve a generated output using the model's internal knowledge—a task akin to "writing and editing." In contrast, our Progressive Reasoning-based Generation module was designed for the distinct purpose of selecting and distilling reliable evidence from a noisy pool of externally retrieved, multi-document sources, a task more akin to "intelligence analysis and verification." Therefore, our novelty is in adapting this reflective mechanism to solve a unique RAG challenge: preparing high-quality external evidence for complex downstream reasoning.

#Q4:Experiments should include other prompting methods such as Chain of Thought, In-context Learning (Few Shot), and medical agent based prompting methods such as MedAgent (MedAgents: Large Language Models as Collaborators for Zero-shot Medical Reasoning, ACL2024 Findings). This would ensure that IP-RAR is not only outperforming RAG approaches, but also other baseline methods.

Response: Thanks for your suggestion. Following your suggestion, we have added a crucial set of baseline experiments designed to directly contrast our RAG-based approach with non-RAG methods. Specifically, we evaluated the performance of several leading prompting strategies that rely solely on the model's internal, parametric knowledge, without the use of any retriever (Table 1) (lines 588 - lines 614). These include Chain of Thought , Few-Shot prompting, and MedAgents, for which we have now added a brief introduction in the Introduction section (lines 69 - lines 72). The purpose of this comparison is to clearly demonstrate that for our specific cross-document question-answering task, which requires precise, multi-source evidence, the Retrieval-Augmented Generation (RAG) paradigm is indispensable. By comparing the performance of our complete IP-RAR system against these non-RAG baselines, we can quantitatively show the significant performance gains afforded by external knowledge retrieval. This, in combination with our comparisons against other RAG methods (like CRAG and SELF-RAG), serves to prove that IP-RAR not only excels among RAG approaches but is also fundamentally superior to baseline methods that do not leverage an external knowledge corpus.

#Q5:How the Protein, Gene, and Biomarkers are ensured that they do not have redundant entities?

Response: Thanks for your suggestion. We describe our solution in the "Entity Normalization in Triplet Extraction" section. This normalization process is specifically designed to address the issue of redundancy: Phase 1 (Database-based Candidate Retrieval): For each entity extracted from the text , we use the all-MiniLM-L6-v2 embedding model to retrieve the top 5 most similar standardized terms from authoritative databases (UniProt/MeSH). Phase 2 (LLM-based Contextual Adjudication): Subsequently, GPT-4o mini utilizes the context from the original text to select the most semantically appropriate term from these 5 candidates to serve as the final, standardized entity. This process effectively handles synonyms and abbreviations, thereby mapping different expressions to the same standard entity and preventing redundancy.

#Q6:The relationship has disease indication, but there is no node type for the indication such as symptom. Does this mean symptom is included in the disease node type?

Response: Thanks for your suggestion. In the current version of BioStrataKG, in order to simplify the ontology of the knowledge graph, we do consider "symptoms" as part of the concept of "disease" and classify them uniformly as "Disease" nodes.

#Q7: Including inference time for each method would support that IP-RAR's performance gain is not too costly.

Response: Thanks for your suggestion. To demonstrate that the performance advantages of our IP-RAR framework do not come at an excessive computational cost (lines 615 - lines 629), we analyzed its inference time and GPU memory usage in a direct comparison against a strong baseline, SELF-RAG, with all tests performed on a single NVIDIA A100 GPU. For a typical query, IP-RAR has an average inference time of 26.7 seconds and a peak GPU memory usage of only 3GB. In contrast, SELF-RAG takes 32.4 seconds and requires a peak of 78GB of GPU memory. Therefore, IP-RAR achieves its state-of-the-art accuracy while also being faster and vastly more computationally efficient than the baseline method, demonstrating the advanced and practical nature of its architecture.

Response to Reviewer #2:
#Q1:The paper asserts capabilities (e.g., drug repurposing) but lacks concrete examples or experimental validation. I would also suggest the introduction of keywords that help in understanding the scope and depth of the tool's application.

Response: Thanks for your suggestion. We have performed a systematic revision of the language throughout the entire manuscript. In multiple sections, including the Abstract, Key Points, Introduction (lines 126 - lines 130), Figure Captions, and Discussion (lines 881 - lines 884), we have replaced overly broad verbs and nouns with more precise phrasing. For example, "drug repurposing" has been revised to "generating hypotheses for drug repurposing," and "accelerating scientific discovery" has been refined to "accelerating the hypothesis generation phase of scientific discovery." Through these changes, we have clearly defined the tool's role as a powerful hypothesis generation and decision support tool, rather than an end-to-end discovery platform.

#Q2:Missing fundamental metrics (node degree distribution, edge cardinality) and graph measures (e.g., centrality)

Response: Thanks for your suggestion. We completely agree that introducing classic graph-theoretic metrics provides a deeper understanding of the knowledge graph's structural properties. As you pointed out, we had already provided initial statistics on the distribution of node and relationship types in Figure 2(b). Building on this, in our revised manuscript, we have supplemented this with the average degree and the node degree distribution (lines 205 - lines 207). We believe this enhanced analysis more comprehensively characterizes the topology and scale of BioStrataKG, highlighting the

Powered by Editorial Manager® and ProduXion Manager® from Aries Systems Corporation

most influential nodes and the overall connectivity, thereby giving readers a clearer understanding of its structural features.

#Q3:No clarification on distinguishing genes/proteins (both use UniProt IDs) or mapping relationships to reference ontologies (e.g., RO)

Response: Thanks for your suggestion. Differentiating between genes and proteins from text is a classic challenge in biomedical natural language processing. Our method addresses this through a two-step process. First, for each potential entity, the LLM analyzes its surrounding sentence context. If the text contains explicit identifiers, such as 'gene X' or 'expression of Y,' the model assigns the Genetype. In other cases, particularly when the text pertains to function, structure, or binding, the model assigns the Proteintype. Once the entity type is determined, the normalization process is performed strictly within that scope: entities marked as Gene are disambiguated against gene entries in UniProt, while those marked as Protein are disambiguated against protein entries. We fully agree that the RO is the recognized reference ontology for standardizing relationships in the biomedical domain. In the current version of our work, we defined a custom set of relationship types. This set was designed for pragmatic reasons, with the primary goal of effectively supporting our core downstream task: building the BioCDQA dataset. This approach facilitated more rapid development and allowed us to precisely capture the information required for our question-answering task.

#Q4:The large use of LLMs in the pipeline raises the concern of hallucinations. The authors should address how they mitigate this risk, especially since hallucinations can affect the accuracy of the entire pipeline, from query to final output. In particular, how are hallucinations handled at each stage of the process, from academic database query to knowledge graph & Q/A dataset generation?

Response: Thanks for your suggestion. We fully agree that mitigating the risk of hallucination in Large Language Models is critical for ensuring the accuracy of our entire pipeline. To this end, we have designed specific mitigation strategies for each key stage of our process:
(1)In the Knowledge Graph Construction Stage: We employ a strict entity normalization process. This process requires every extracted entity to be mapped to a corresponding official entry in authoritative databases (e.g., MeSH and UniProt). This fundamentally constrains the model from fabricating entities, ensuring the authenticity of the nodes in our knowledge graph.
(2)In the Dataset Construction Stage: We conducted rigorous manual verification for all machine-generated question-answer pairs. This process involved domain experts tracing back to the source literature to check the accuracy and faithfulness of each answer, thereby guaranteeing that every answer in the BioCDQA dataset is grounded in evidence.
(3)In the Question-Answering Stage: We have implemented a multi-layered hallucination suppression framework. First, the core principle of Retrieval-Augmented Generation (RAG) is to ground the LLM's response using retrieved text from real literature. Second, the Progressive Reasoning and Self-reflection mechanism within our framework further filters out irrelevant text chunks that could introduce noise, preventing the LLM from reasoning based on unrelated information. Finally, this evidence-based requirement for answers significantly suppresses any ungrounded generation.

#Q5:The paper mentions that the QA dataset is generated from a knowledge graph using an LLM-based procedure, and the EM metrics and the GPT scoring system are used to evaluate the performance of another LLM. There seems to be a risk of a circular evaluation loop, where the same technology is used to assess itself.

Response: Thanks for your suggestion. We acknowledge the potential bias in using an LLM to evaluate the output of another LLM. However, given the practical constraints of large-scale human annotation, employing a more capable, advanced LLM (like GPT-4) as an evaluator is a widely accepted and pragmatic solution in the current NLP landscape, especially for complex generation tasks with non-deterministic answers that require deep semantic understanding. To further mitigate bias, we intentionally used a heterogeneous model setup: our question-answering generation relies primarily on the

Powered by Editorial Manager® and ProduXion Manager® from Aries Systems Corporation

DeepSeek series, while performance evaluation is conducted using OpenAI's GPT-4 series. This design effectively circumvents self-preference bias, preventing a model from receiving an unfairly high score due to stylistic similarities with the evaluator. Furthermore, to guarantee the absolute reliability of our benchmark dataset, we implemented an exceptionally rigorous manual curation protocol during its construction (lines 458 - lines 475). From an initial pool of over 5,000 machine-generated QA pairs, we filtered down to 1,183 high-quality entries. This process was independently conducted by two authors with biomedical backgrounds, who evaluated each pair against a comprehensive set of criteria. These criteria included factual correctness and faithfulness (ensuring every assertion in the answer is clearly supported by and traceable to the source literature, and is free of hallucinations), completeness (no omission of critical information), relevance and conciseness(the answer is focused and without redundant details), and the quality of the question itself (clear and unambiguous). For each QA pair, reviewers meticulously compared the question, the generated answer, and the source text, and only those that met all standards were provisionally accepted. Following the independent reviews, the results were compared, and only QA pairs with consensus approval from both reviewers were retained for the final dataset.

#Q6:It is unclear the reason for which the authors have chosen Neo4j for their knowledge graph when the relationships are modeled using triples rather than properties. The properties of nodes and edges are not discussed in the paper, though the supplementary materials mention extracting node properties from texts. It would be helpful to explain how these properties are derived, and how issues like data duplication are prevented, especially when the same entity may be linked to different attributes across various texts. Additionally, the authors mention extracting properties from nodes but do not discuss any similar extraction for edges. Since relationships are central to the knowledge graph, it would be essential to include property extraction for edges to enhance the graph's semantic richness.

Response: Thanks for your suggestion. Our choice of Neo4j is based on its mature ecosystem, a powerful visualization interface, and the intuitive Cypher query language. Although our core data model is based on triplets, Cypher is more convenient for performing native graph operations such as multi-hop queries and pathfinding, which is highly valuable for exploratory analysis. In fact, every relationship (edge) in our knowledge graph includes a crucial property: the Evidence Source, which is the ID of the original paper that supports the relationship. This attribute is fundamental to ensuring that every relationship in our knowledge graph is traceable. We will clarify and emphasize this point in our revised manuscript (lines 246 - lines 250). To ensure the consistency of our knowledge graph, we designed a clear de-duplication strategy. For Nodes, we prevent node duplication through a rigorous Entity Normalization process, which maps different textual mentions to a single, canonical entity ID. For Relationships, we allow multiple edges of different types to exist between two nodes, but we de-duplicate any identical (node1, relation, node2) triplets to ensure their uniqueness.

#Q7:The authors do not clarify which vector database they are using. Is it ChromaDB? Moreover, the chunking strategy is not explained. How are large texts split into manageable pieces for processing?The use of the Max factor in the similarity formula implies that every chunk is compared with all other chunks. This approach is computationally expensive, especially as the number of articles increases. The authors should discuss the scalability of their method and propose potential optimizations for larger datasets.

Response: Thanks for your suggestion. Our retrieval pipeline does not utilize a dedicated vector database like ChromaDB; instead, its core is based on the Contriever model. The recommended implementation for Contriever involves pairing it with an efficient vector indexing library. In our implementation, we follow this best practice: the vector embeddings generated by Contriever are stored in an IVF+HNSW index built using the FAISS library. This indexing structure enables Approximate Nearest Neighbor (ANN) search, which significantly reduces the computational complexity of retrieval from linear time, O(N) (where N is the total number of text chunks), to logarithmic time, O(log N). This means that even as the number of articles in our corpus grows from tens of thousands to millions or more, the retrieval time will only

Powered by Editorial Manager® and ProduXion Manager® from Aries Systems Corporation

increase very slowly. Therefore, our method is computationally efficient and fully capable of scaling to much larger datasets without requiring architectural changes to the core retrieval component. For the full text of the papers, we employed the Recursive Character Text Splitter strategy (lines 321 - lines 329). We set the chunk size to 500 characters with a 100-character overlap between consecutive chunks. This strategy was chosen because it effectively attempts to keep sentences whole when splitting large documents, thereby maximally preserving local context and reducing information loss from improper segmentation. We add these crucial technical details to the Methods section of our revised manuscript to improve its clarity.

#Q8:The related work section does not adequately introduce Contriever and CRAG, two methods that appear relevant to the problem. The authors should provide more detailed comparisons to these systems. Additionally, bioinfo-0 and dmiip3 are mentioned but not introduced.

Response: Thanks for your suggestion. We have expanded the descriptions of Contriever (lines 54 - lines 57) and CRAG (lines 63 - lines 67) in the Introduction section, detailing their core mechanisms. Regarding bioinfo-o and dmiip3, we have now added a brief introduction in the Results section (lines 634 - lines 643) to establish a clear benchmark for our performance comparison and to provide the necessary background information for the reader.

#Q9:Citation [1] is not pertinent and should be revised.

Response: Thanks for your suggestion. We have replaced this citation with a review article that is more directly relevant to our work on the applications of LLMs in the biomedical domain.

#Q10:Figure 1: It is not clear why a query would be placed in the biological data sources box. A query should typically be used to extract or search data, not act as data itself. Moreover, there is a lack of clarity on the transition between steps b and c in the figure. Figure 3b is introduced without proper context. It should be clearly explained in the text as an example of the relevant concept, as it seems to play a key role in the argument but is not sufficiently introduced.

Response: Thanks for your suggestion. To resolve the logical issue of placing a "Query" within "Knowledge Sources," we have restructured the figure (Figure 1) to show the "Knowledge Source" (i.e., papers) separately from the "Query," which now acts as a direct input to the processing pipeline. Furthermore, to clarify the transition from the process to its applications, we have replaced the single generic arrow with more specific arrows, explicitly linking the "Knowledge Graph" and the "Answer" outputs to their respective downstream applications in part (C). To improve the figure's clarity, we will revise the caption for Figure 3 in our revised manuscript (the caption for Figure 3). The updated caption will provide a more detailed explanation of the 'Relevant Text Chunks' section in Figure 3(B), making it clear that these are evidence fragments retrieved from the source literature by our various retrieval strategies in part (A) (e.g., 'Question Retrieval,' 'Keyword matching,' and 'Virtual Answer Retrieval'), each marked with its source.

#Q11:In Figure 4, the interaction with the user is unclear. The figure suggests that the system involves expert interaction, but this is never elaborated on in the text.

Response: Thanks for your suggestion. Figure 4 illustrates an automated workflow designed to provide decision support for domain experts. The system automatically generates a complete, evidence-based analytical report, upon which an expert can base their subsequent decisions. The key point of potential confusion lies with the human icon in Figure 4(b). We wish to clarify that this icon represents a "patient" in a clinical case study (specifically, a patient with colorectal neoplasms and lymphoma), and not a "user" who is interacting with our system. We will clarify this in the figure caption of our revised manuscript to eliminate any ambiguity.

#Q12:The authors use @prefix ex: <http://example.org/> as a namespace in the turtle representation of the graph. This is unclear to me.

Powered by Editorial Manager® and ProduXion Manager® from Aries Systems Corporation

	# Response: Thanks for your suggestion. We completely agree that using <http://example.org/> as a namespace was unclear. This was indeed a placeholder in our data export scripts. Following your suggestion, we have now corrected our data files. We have updated this placeholder to a unique and persistent namespace that points to our project's GitHub repository (e.g., https://github.com/yichun10/BioCDQA/schema#). Furthermore, we have added a detailed SCHEMA.md file to the GitHub repository (https://github.com/yichun10/BioCDQA/blob/main/SCHEMA.md) to clearly define and describe all the nodes, relationships, and properties in our knowledge graph.<br><br>#Q13:Additionally, providing an online endpoint for Neo4j rather than just a dump would improve the system's accessibility for future research.<br><br># Response: Thanks for your suggestion. Regarding the suggestion to provide an online Neo4j endpoint for live queries, we agree this is a valuable idea for enhancing data accessibility. However, considering the long-term maintenance costs and resources required, we have prioritized providing the data in two more stable and reproducible formats at this stage. We offer: The complete Neo4j database dump file, which allows other researchers to perfectly replicate our graph environment; An open RDF-formatted version of the graph, which has been uploaded to the GigaDB FTP server in accordance with the journal's data sharing standards. We believe these two options will fully support the reuse and exploration of our data by the research community.<br><br>#Q14:In Figure 2, is the Academic Database PubMed?<br><br># Response: Thanks for your suggestion. Yes, the papers used in our study were primarily sourced from PubMed. This is described in the "Paper Collection" section of our manuscript.
**Additional Information:**	
**Question**	**Response**
Are you submitting this manuscript to a special series or article collection?	No
**Experimental design and statistics**<br><br>Full details of the experimental design and statistical methods used should be given in the Methods section, as detailed in our Minimum Standards Reporting Checklist. Information essential to interpreting the data presented should be made available in the figure legends.<br><br>Have you included all the information requested in your manuscript?	Yes
**Resources**<br><br>A description of all resources used, including antibodies, cell lines, animals and software tools, with enough information to allow them to be uniquely	Yes

Powered by Editorial Manager® and ProduXion Manager® from Aries Systems Corporation

identified, should be included in the Methods section. Authors are strongly encouraged to cite Research Resource Identifiers (RRIDs) for antibodies, model organisms and tools, where possible.<br><br>Have you included the information requested as detailed in our Minimum Standards Reporting Checklist?	
**Availability of data and materials**<br><br>All datasets and code on which the conclusions of the paper rely must be either included in your submission or deposited in publicly available repositories (where available and ethically appropriate), referencing such data using a unique identifier in the references and in the "Availability of Data and Materials" section of your manuscript.<br><br>Have you have met the above requirement as detailed in our Minimum Standards Reporting Checklist?	Yes
GigaScience has policies and guidelines in place for the use of generative AI-writing tools such as ChatGPT. If you have used such writing tools to assist with writing the manuscript this must be declared and cited in the text. Authors should not list AI-writing tools and other AI-assisted technologies as an author or co-author and should acknowledge that they are fully responsible for text generated or refined by AI-writing tools.<p><br><br>A summary of use (particularly in the introduction or among methods) needs to be included at the end of the paper, and the outputs should also be included as a supplementary file hosted in GigaDB or other open repositories. Please read our guidelines for more information. <p><br><br>By submitting to GigaScience, you are aware of the journal's AI-writing tools policy, and if you have declared use of	No

such tools below, you have acknowledged this where appropriate in your manuscript and have made a summary of use and outputs available. <p> <b>AI-assisted writing tools have been used in the preparation of this manuscript?	

Manuscript

Click here to access/download;Manuscript;Knowledge Mining in Biomedical Literature.pdf

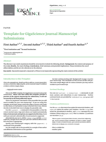

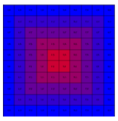

Journal of X, 2025, 1–14

Manuscript in Preparation
Research

RESEARCH

A Retrieval-Augmented Knowledge Mining Method with Deep Thinking LLMs for Biomedical Research and Clinical Support

Yichun Feng[1,2,3], Jiawei Wang[4], Ruikun He[5], Lu Zhou[3,*] and Yixue Li[2,3,6,7,8*]

[1]School of Advanced Interdisciplinary Sciences, University of Chinese Academy of Sciences, 100049 Beijing, China and [2]Key Laboratory of Systems Health Science of Zhejiang Province, School of Life Science, Hangzhou Institute for Advanced Study, University of Chinese Academy of Sciences, 310024 Hangzhou, China and [3]Guangzhou National Laboratory, No. 9 XingDaoHuanBei Road, Guangzhou International Bio Island, 510005 Guangzhou, China and [4]Department of EEIS, University of Science and Technology of China, 230026 Hefei, China and [5]BYHEALTH Institute of Nutrition & Health, 510663 Guangzhou, China and [6]GZMU-GIBH Joint School of Life Sciences, The Guangdong-Hong Kong-Macau Joint Laboratory for Cell Fate Regulation and Diseases, 511436 Guangzhou, China and [7]School of Life Sciences and Biotechnology, Shanghai Jiao Tong University, 200240 Shanghai, China and [8]Shanghai Institute of Nutrition and Health, Chinese Academy of Sciences Shanghai, 200030 Shanghai, China

[*]Correspondence address. Yixue Li, Guangzhou National Laboratory, No. 9 XingDaoHuanBei Road, Guangzhou International Bio Island, 510005 Guangzhou, China. E-mail: li_yixue@gzlab.ac.cn; Lu Zhou, Guangzhou National Laboratory, No. 9 XingDaoHuanBei Road, Guangzhou International Bio Island, 510005 Guangzhou, China. E-mail: zhou_lu@gzlab.ac.cn

Abstract

Background. Knowledge graphs and large language models (LLMs) are key tools for biomedical knowledge integration and reasoning, facilitating structured organization of scientific articles and discovery of complex semantic relationships. However, current methods face challenges: knowledge graph construction is limited by complex terminology, data heterogeneity, and rapid knowledge evolution, while LLMs show limitations in retrieval and reasoning, making it difficult to uncover cross-document associations and reasoning pathways.
Results. We propose a pipeline that uses LLMs to construct a biomedical knowledge graph (BioStrataKG) from large-scale articles and builds a cross-document question-answering dataset (BioCDQA) to evaluate latent knowledge retrieval and multi-hop reasoning. We then introduce Integrated and Progressive Retrieval-Augmented Reasoning (IP-RAR) to enhance retrieval accuracy and knowledge reasoning. IP-RAR maximizes information recall through Integrated Reasoning-based Retrieval and refines knowledge via Progressive Reasoning-based Generation, using self-reflection to achieve deep thinking and precise contextual understanding. Experiments show that IP-RAR improves document retrieval F1 score by 20% and answer generation accuracy by 25% over existing methods.
Conclusions. The IP-RAR helps doctors efficiently integrate treatment evidence to inform the development of personalized medication plans and enables researchers to analyze advancements and research gaps, accelerating the hypothesis generation phase of scientific discovery and decision-making.

Key words: Large Language Model, Knowledge Graph, Knowledge Mining, Retrieval-Augmented Generation, Deep Thinking

Compiled on: August 7, 2025.
Draft manuscript prepared by the author.

Key Points

- We construct the Biomedical Stratified Knowledge Graph (BioStrataKG) from large-scale research articles, which combines an entity-level graph with a document-level graph to support rich biomedical relationship discovery.
- We introduce BioCDQA — a biomedical cross-document question answering dataset built upon BioStrataKG — to address the limitations of existing QA datasets.
- We propose the IP-RAR framework, which combines Integrated Reasoning-based Retrieval with Progressive Reasoning-based Generation, enhanced by a self-reflection mechanism and LLMs with deep-thinking capabilities, to improve the accuracy and contextual relevance of answer generation.
- Experimental results show that IP-RAR consistently outperforms existing methods across all evaluation metrics, achieving state-of-the-art performance.
- The proposed framework supports a variety of downstream biomedical applications, including analysis for drug synergy and antagonism, generating hypotheses for drug repurposing, providing evidence for precision medicine, and assisting in scientific knowledge discovery, ultimately assisting clinical decision-making and accelerating biomedical research.

Introduction

The advancement of large language models (LLMs) has significantly accelerated progress in natural language processing (NLP), particularly in complex tasks like question answering (QA), with notable breakthroughs in specialized fields such as biomedical science [1]. However, despite these advancements, the application of LLMs for knowledge mining in specialized domains like biomedical science remains limited, where rigorous precision and robust evidence validation are essential for extracting meaningful insights [2]. Biomedical articles is vast, containing massive amounts of information [3], much of which remains underutilized. This inefficiency hinders scientific discovery and limits our ability to address complex biomedical challenges that require deep connections and advanced reasoning [4]. The wealth of knowledge within biomedical research, if effectively harnessed, could drive breakthroughs in critical areas such as clinical medicine, pharmacology, and molecular biology [5].

In biomedical knowledge mining, the construction of knowledge graphs is essential for enhancing information integration and reasoning capabilities. However, this process presents several challenges. First, the biomedical domain has a highly complex terminology system, with numerous synonyms, polysemous terms, and hierarchical classifications, making the precise extraction of entities and relationships particularly difficult [6]. Second, biomedical knowledge is fragmented and heterogeneous, spanning research papers, clinical reports, and databases, posing a fundamental challenge in unifying and linking these diverse data sources effectively [7]. Additionally, the rapid evolution of biomedical research constantly introduces new findings, requiring knowledge graphs to be dynamically expandable to maintain long-term relevance [8]. Despite these challenges, high-quality knowledge graphs can explicitly capture the intricate relationships among genes, proteins, diseases, and drugs, providing structured support for cross-document reasoning and deep knowledge extraction [9].

Building a high-quality biomedical QA dataset that effectively leverages knowledge graphs is crucial for evaluating and enhancing models' abilities in cross-document reasoning and knowledge integration. While existing datasets such as MASH-QA [10], which addresses multi-span questions across long documents, BioASQ [11], which focuses on biomedical semantic indexing and QA, MEDHOP [12], which targets multi-hop reasoning across multiple paragraphs, MedicationQA [13], which enhances understanding of medication-related queries, MedMCQA [14], which provides large-scale multiple-choice QA for medical examinations, and PcQA [15], which facilitates structured knowledge graph question answering, offer valuable benchmarks, they often fail to capture the deep, interconnected knowledge hidden within multiple sources.

Retrieval-Augmented Generation (RAG) has emerged as a promising approach for biomedical knowledge mining by integrating external knowledge into the response generation process [16]. However, its effectiveness relies on refining the retrieval mechanism to focus on high-quality, contextually relevant content, ensuring more accurate and reliable knowledge extraction [17]. Against this backdrop, advanced retrieval models like Contriever [18] have provided a powerful foundation for RAG systems by using unsupervised contrastive learning to learn high-quality text representations without relying on labeled data. SELF-RAG [19] dynamically adjusts the retrieval process by evaluating the quality of the retrieved content, thereby enhancing the accuracy of long-form answers in complex domains like biomedical research. Other innovative approaches include GraphRAG [20], which structures retrieved passages into graphs to capture relationships between them, improving coherence and relevance. CRAG [21] further refines the process by using a lightweight evaluator to assess retrieved passages and triggering new, web-based corrective searches for irrelevant documents, thereby actively improving the quality of the knowledge source before generation. Additionally, RAPTOR [22] organizes retrieval results into a tree structure, recursively summarizing data to facilitate more effective reasoning. Further extending this paradigm, agent-based frameworks like MedAgents[23] structure LLMs into a collaborative group of experts that can collectively reason and utilize tools to solve complex medical problems.

In addition to extracting high-quality information from external knowledge bases using RAG, LLMs have recently made breakthrough progress in their inherent reasoning capabilities. For instance, OpenAI's GPT-o1 leverages reinforcement learning and a chain-of-thought mechanism to demonstrate reasoning on complex tasks in mathematics, programming, and science—achieving performance comparable to that of PhD-level experts [24]. Meanwhile, DeepSeek has released its reasoning-oriented model, DeepSeek-R1 [25], which performs on par with GPT-o1 in logical reasoning, mathematical computation, and code generation, yet its training cost is only a small fraction of that of GPT-o1. These breakthroughs not only expand the functionalities of large models but also provide stronger intrinsic support for complex domains such as biomedical knowledge mining, helping to yield more accurate answers in cross-document and multi-layer relational reasoning tasks.

In this paper, we present a comprehensive framework for biomedical knowledge mining, as illustrated in Figure 1. This framework processes diverse biomedical knowledge sources, such as research papers and user queries, through a systematic pipeline that involves constructing knowledge graphs and generating precise responses to user queries. Within this pipeline, LLMs collaborate with retrieval-augmented reasoning techniques to extract, organize, and integrate domain-specific knowledge. First, an entity-level knowledge graph and a document-level knowledge

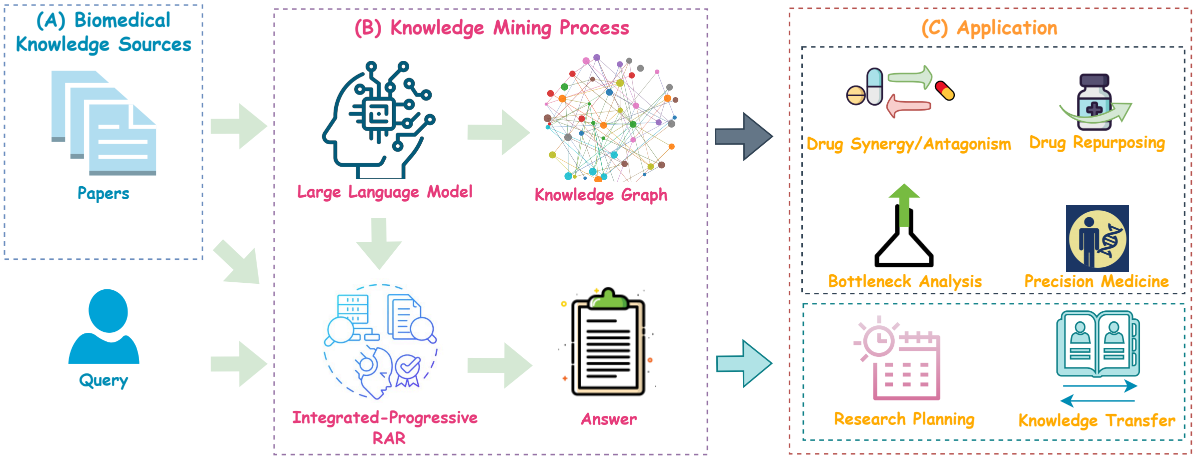

Figure 1. Overview of the proposed framework for biomedical knowledge mining. (A) A Biomedical Knowledge Source, consisting of a large corpus of research papers, provides the foundational knowledge for the system. A user's query serves as the direct input to trigger the question-answering process. (B) A knowledge mining pipeline leverages a LLM to build a knowledge graph from the papers and utilizes the IP-RAR approach to generate a precise answer. (C) The outputs from the pipeline enable diverse applications: the generated Knowledge Graph supports analytical tasks such as generating hypotheses for drug repurposing and analysis for drug synergy/antagonism, while the precise Answers from the QA process facilitate applications such as supporting research planning and facilitating knowledge transfer.

graph are constructed from large-scale biomedical research papers and merged into the Biomedical Stratified Knowledge Graph (BioStrataKG). The entity-level graph captures relationships between biomedical entities such as genes, proteins, diseases, and drugs, while the document-level graph represents connections between research papers based on shared methods, datasets, and research directions, as well as citation and reference relationships. Together, these knowledge graphs uncover complex biomedical relationships and reveal latent patterns, thereby supporting advanced knowledge discovery. Since existing datasets do not meet the requirements for cross-document reasoning and knowledge mining, we develop a new biomedical cross-document question answering dataset, called BioCDQA, based on the BioStrataKG to support these tasks. Second, we propose the Integrated and Progressive Retrieval-Augmented Reasoning (IP-RAR) framework, which synergizes Integrated Reasoning-based Retrieval with Progressive Reasoning-based Generation to seamlessly integrate relevant knowledge, enabling more precise and contextually relevant reasoning. The framework first maximizes the recall of pertinent information from large-scale biomedical articles through Integrated Reasoning-based Retrieval, ensuring comprehensive coverage of relevant data. Subsequently, the progressive reasoning-based generation mechanism refines and enhances the extracted knowledge while leveraging a self-reflection mechanism to continuously optimize the accuracy and contextual relevance of the answers. Ultimately, LLMs with deep thinking capability further refine the reasoning process to derive a high-quality answer. The IP-RAR framework enables precise reasoning and knowledge integration, providing effective technical support for a wide range of downstream biomedical research applications, including analysis to support drug synergy/antagonism studies, generating hypotheses for drug repurposing, and informing precision medicine strategies. This framework helps doctors quickly identify and integrate relevant treatment evidence from vast biomedical articles, enabling more precise personalized medication plans. It also allows researchers to systematically analyze cutting-edge advancements and potential research gaps, accelerating research strategy formulation and decision-making. These advancements in knowledge mining pave the way for more efficient and accurate solutions in contemporary biomedical research.

Materials and Methods

Construction of the BioStrataKG

This section introduces the construction method of the BioStrataKG, proposing a document-entity dual-layer representation fusion architecture based on LLMs, which effectively captures complex relationship networks among biomedical entities and establishes a cross-document knowledge association system based on this network. As shown in Figure 2a, the construction process of BioStrataKG begins with large-scale biomedical articles, utilizing GPT-4o mini [26] for fine-grained knowledge extraction, including entity-relationship triple extraction and the structured representation of semantic information such as research methods and fields in the article. Subsequently, cross-document association networks are established through entity co-occurrence analysis, achieving a hierarchical expansion of the knowledge graph from micro-level entity relationships to macro-level document associations. The entity types and relationship types within the knowledge graph are detailed in Figure 2b.

Data Collection and Processing

Paper Collection. The papers included in our dataset are all sourced from the open-access database PubMed, which allows anyone to download and access biomedical and life sciences article via PMID. We have downloaded over 8,000,000 papers, extracting their titles, abstracts, publication years, keywords, and citation relationships.
Paper selection. We filtered papers based on keywords related to lung cancer, breast cancer, and colorectal cancer, focusing on studies involving single-cell analysis, pharmacology, and clinical trials. We further narrowed our selection to papers published within the last decade and those with more than 10 citations. In total, we selected 15,585 papers to build the knowledge graph for generating the bioCDQA dataset. Some of the basic statistics of the papers involved in our dataset are detailed in Supporting Information Fig. S2.
PDF to Markdown Conversion. While we initially attempted to use XML files, we found that for a portion of the open-access literature, the XML versions contained only metadata and abstracts, with the full text being either missing or poorly formatted. As a key objective of our study is full-text knowledge mining, the PDF format was essential for obtaining the most complete information. Therefore, we adopted a hybrid approach, retaining available metadata and abstracts while processing the complete full text from the corre-

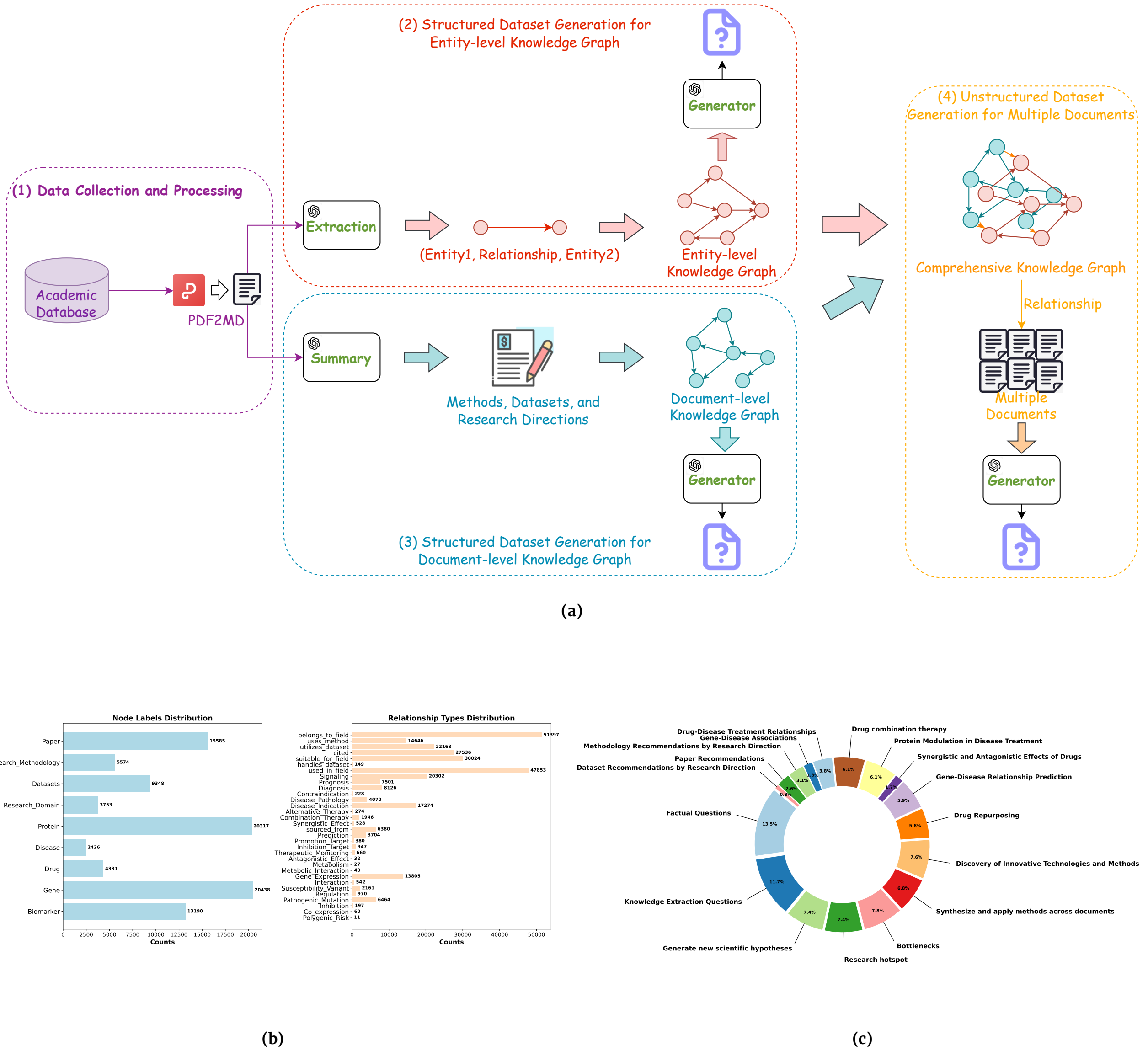

Figure 2. Construction Pipeline and Statistical Analysis of the Dataset. (a) BioStrataKG and BioCDQA Construction Workflow Diagram. (1) Data Collection and Processing: The process begins by converting research papers from PDF to markdown (MD) format to facilitate content extraction. (2) Structured Dataset Generation for Entity-level Knowledge Graph: An LLM is used to extract entities and relationships (Entity1, Relationship, Entity2), which are then standardized to construct an entity-level knowledge graph. This graph supports downstream tasks such as generating hypotheses for drug repurposing, drug interaction analysis for comorbid conditions , and gene-disease associations. (3) Structured Dataset Generation for Document-level Knowledge Graph: Summarization is performed using an LLM to extract key aspects such as methods, datasets, and research directions. The resulting document-level knowledge graph facilitates tasks such as research strategic planning and research paper recommendations. (4) Unstructured Dataset Generation for Multiple Documents: Integration of the entity-level and document-level knowledge graphs produces a comprehensive knowledge graph. This integrated graph enables connections across multiple documents and supports downstream tasks such as content-based factual questioning, knowledge transfer, trend analysis, and hotspot detection. (b) Statistics of node labels and relationship types in BioStrataKG. (c) Statistics of question categories in BioCDQA.

sponding PDFs to ensure comprehensive data coverage. Since the papers downloaded from the database are in PDF format, we convert them to Markdown format using the marker toolkit [27].

Knowledge Graph Construction

We leverage the GPT-4o mini to extract biomedical-related triplet information (e.g., genes, proteins, diseases, drugs, etc.) from research papers to construct an entity-level knowledge graph. Simultaneously, we structurally summarize the research methods, datasets used, and research domains of the papers to build a document-level knowledge graph. On this basis, each paper serves as a node within the document-level knowledge graph. By identifying recurring biomedical terms, related datasets, research methods across multiple papers, and analyzing citation and reference relationships, we establish inter-document linkages and achieve comprehensive knowledge integration. This cross-document information connection not only facilitates the construction of high-quality cross-document question-answering datasets but also provides richer contexts and deeper opportunities for knowledge discovery in biomedical knowledge mining tasks. Additionally, we have open-sourced this knowledge graph, which can be accessed at https://github.com/yichun10/BioCDQA.git. Beyond supporting our dataset, the knowledge graph can serve as a valuable resource for researchers, aiding in knowledge discovery, identifying trends, and exploring relationships between biomedical entities and research methods. The knowledge graph contains

94,962 nodes and 290,403 relationships. Detailed descriptions of the nodes and relationships are provided Figure 2b. The knowledge graph has an average degree of 6.11, and its node degree distribution is provided in Supporting Information Fig. S3.

Entity-Level Knowledge Graph

Triplet Extraction Based on LLMs. In the KG, a triplet is the fundamental unit of information used to represent entities and their relationships. Specifically, a triplet is defined as (e_1, r, e_2), where e_1 and e_2 are entities, representing nodes in the knowledge graph, and r is a relation, representing the connection between the entities. We utilize the GPT-4o mini to extract triplets from the abstracts of papers. The entity types (ET) we extract are Gene, Protein, Drug, and Disease. The relation types (RT) we define and Triplet Extraction Prompt are detailed in Supporting Information Fig. S4.

Entity Normalization in Triplet Extraction. Entity normalization is essential for ensuring consistent and standardized representation of extracted entities in knowledge graph construction. To address this, we adopt a two-stage approach that combines domain-specific databases with advanced language models. First, we reference the MeSH database [28] for diseases and drugs, and the UniProt database [29] for genes and proteins. Using the all-MiniLM-L6-v2 [30] embedding model, we retrieve the top 5 candidate terms from these databases by generating dense vector representations of the extracted entities, ensuring computational efficiency and high recall. Next, GPT-4o mini evaluates the semantic and contextual alignment between the extracted entity and the retrieved candidates to identify the most appropriate standardized term. This approach resolves ambiguities, such as homonyms or abbreviations, and ensures that entities are contextually accurate and semantically consistent.

Constructing Entity-Level Knowledge Graphs. Once the standardized triplets (e_1, r, e_2) are obtained, the next step is to construct the entity-level knowledge graph, where entities e_1 and e_2 are represented as nodes and the relation r forms the directed edge between them. The process begins with node creation, where each unique entity from the standardized triplets is instantiated as a node in the graph and categorized based on its type (e.g., Gene, Protein, Drug, Disease) to ensure consistency and facilitate downstream analysis. Relations are then represented as directed edges, labeled with their relation types, connecting the corresponding nodes and formalizing the interactions between entities. In practice, every relationship in the knowledge graph includes a key attribute: the Evidence Source, which is the ID of the original paper supporting the relationship. This attribute is fundamental for ensuring that every relationship in the graph is traceable to its origin. The graph supports multiple labeled edges between the same nodes, capturing diverse relationships such as a gene being associated with multiple diseases, and manages complexity by representing each distinct relationship as a separate edge. During the construction process, rigorous checks are applied to detect and handle duplicate nodes and edges, ensuring that new relationships are accurately linked to existing nodes, thereby preventing redundant entries. The completed KG is stored in the Neo4j graph database, where Neo4j's Cypher query language enables efficient and intuitive exploration of the graph. Users can retrieve complex relationship networks, such as querying all drugs related to a specific disease or identifying potential relationship chains between entities, facilitating flexible and powerful knowledge discovery.

Document-Level Knowledge Graph

Paper Information Extraction Based on LLMs. We utilize the GPT-4o mini to extract the fundamental research methods of each paper, the datasets used, and the respective research domains. These extracted elements, along with the paper titles, are considered as nodes. Each node and its relationship constitute a document-level triplet (e_1, r, e_2), facilitating subsequent knowledge graph construction. The prompt for extraction of paper information is presented in Supporting Information Fig. S5.

Normalization. To achieve standardization and consistency of entity names in document-level knowledge graphs, we propose a standardization workflow based on vector matching and LLM-assisted decision-making. The workflow processes each entity name in the triples sequentially, ensuring that all potential duplicate entities are incorporated into subsequent matching and standardization steps. For each entity name, the existing vector database (e.g., ChromaDB) is traversed to calculate the cosine similarity with other entities. If a record with a similarity score greater than 0.5 is found, the two entity names are considered to refer to the same concept (e.g., a method or dataset), and the current entity name is merged with the existing record. Conversely, if no matching entity name is found, the current entity name is inserted into the vector database for use in future standardization steps. Building on the initial steps, GPT-4o mini further optimizes merged entity names by identifying the most semantically relevant and contextually accurate choices, ensuring standardization, precision, and robustness in the final output.

Constructing Document-Level Knowledge Graphs from Triplets. After normalizing document-level triplets (e_1, r, e_2), the document-level knowledge graph is constructed in a manner similar to the entity-level KG. Each unique entity, including papers, research methods, datasets, and research domains, is instantiated as a node in the graph. Directed edges are created between these nodes to represent the relationships, ensuring an efficient and comprehensive structure for knowledge discovery.

Construction of the Biomedical Cross-Document Question Answering Dataset

Based on BioStrataKG, we introduce a biomedical question-answering dataset—BioCDQA, designed to support cross-document reasoning and biomedical knowledge mining. The construction process of the dataset is illustrated in Figure 2a. The dataset integrates data from both unstructured text and structured knowledge graphs. By extracting information from text, we obtain rich contextual data, while the knowledge graph provides precise relationships between entities such as genes, diseases, drugs, and proteins. These data sources collectively ensure the diversity and practicality of the dataset. This dataset consists of tuples containing the following elements: question, question type, answer, source papers for the answer, and source sentences for the answer. Each tuple includes a natural language question, whose answer is composed of one or more sentences extracted from the source papers. These answers may originate from a single paper or multiple papers and can consist of multiple sentences, either from a single span or dispersed across different sections of various source papers.

The dataset contains a total of 1,183 question-answer pairs, covering 68,428 papers and providing over 1.85 million document chunks available for retrieval. To create this large-scale retrieval corpus, we employed a text chunking strategy. We utilized a Recursive Character Text Splitter to segment the full text of the papers, setting the chunk size to 500 characters with a 100-character overlap between consecutive chunks. This method was chosen for its effectiveness in attempting to preserve sentence integrity when splitting long texts, thereby maximally maintaining local contextual coherence and reducing information loss from improper segmentation. The dataset and the corresponding set of retrievable papers are available at https://github.com/yichun10/BioCDQA.git. The distribution of question types is shown in Figure 2c. The definitions and characteristics of each type are elaborated in the subsequent sections.

Dataset Generation from Unstructured Data

We employ the BioStrataKG to establish relationships among multiple research papers. Randomly selecting 1 to 5 interconnected articles, we input the full text of the chosen papers into GPT-4o mini. This system then formulates pertinent questions from the following perspectives and generates responses based on both the questions and the text.

Factual Questions. In the domain of biology, factual questions typically revolve around specific biological facts, data, or phenomena. Examples include inquiries about the sequence of a particular gene, the function of a specific protein, epidemiological data of a disease, or the detailed steps of a biological process. These questions can be answered through a thorough examination of scientific literature, experimental data, or validated biological databases, ensuring responses are grounded in objective, verifiable information rather than subjective opinions.

Knowledge Extraction Questions. The primary objective of knowledge extraction questions is to identify and extract specific categories of information from a given text, typically presented in a list format. In the biomedical domain, such questions may involve extracting names of drugs, genes, diseases, or symptoms. For instance, when asked, "What are the drugs used to treat lung cancer?", the system must extract relevant drug names from the text and present them in a list, typically identifying three to five commonly used drugs. The focus of these questions is on precise extraction rather than interpretation or reasoning. The goal is to quickly distill key entity information from large volumes of unstructured text, enabling researchers to efficiently retrieve the necessary biomedical data and accelerate literature analysis.

Knowledge Discovery Questions. Knowledge discovery questions represent a key feature of our dataset, aiming to extract deeper insights and foster innovative scientific inquiry. These questions not only facilitate the generation of new hypotheses from the findings and conclusions presented in the literature but also assist in designing subsequent research plans. By analyzing existing literature, these questions help identify hidden themes and emerging research hotspots, providing valuable insights into current trends. Additionally, they empower researchers to analyze publication trends, assess keyword relevance, and evaluate citation metrics, uncovering critical areas of interest and guiding potential future directions in biomedical research. Moreover, knowledge discovery questions allow for the identification of bottlenecks within specific fields, based on comprehensive reviews, which highlights pressing challenges that need addressing. Through the synthesis of information across multiple documents, these questions support the transfer of methodologies and concepts to other research areas, fostering interdisciplinary innovation. Overall, knowledge discovery questions play a pivotal role in advancing the scientific dialogue by tracking the evolution of knowledge, revealing critical nodes in research development, and ultimately guiding future investigations in the biomedical domain.

Dataset Generation from Structured Data

We generate various Cypher query statements based on BioStrataKG, which features two distinct granularity-based knowledge graphs (entity-level and document-level), to extract relevant subgraphs and relationship chains. The Entity-Level Knowledge Graph provides a comprehensive framework for exploring complex biomedical interactions, such as those between diseases, genes, drugs, and proteins. This supports key research areas, including drug repurposing, gene-disease prediction, and treatment optimization. Meanwhile, the Document-Level Knowledge Graph is designed to enhance research efficiency by offering tailored recommendations aligned with specific research directions. These queries retrieve nodes and relationships pertinent to specific research topics, enabling the extraction of focused subgraphs and relationship chains. By leveraging these, GPT-4o mini formulates targeted questions and generates corresponding answers, improving question precision and dataset quality.

Drug Repurposing. Triplet relationships between diseases, genes, and drugs are established to uncover new applications for existing drugs in treating previously unrelated diseases. By leveraging data on drug effects and identifying hidden connections, innovative treatment pathways are discovered.

Gene-Disease Relationship Prediction. Known associations between genes and diseases are analyzed to predict new gene-disease relationships. Patterns and similarities within the data highlight potential genetic markers involved in disease development, aiding early diagnosis and targeted therapies.

Synergistic and Antagonistic Effects of Drugs. Drug interactions are examined with a focus on synergistic (enhancing) or antagonistic (counteracting) effects. For patients with comorbid conditions, harmful interactions are identified, and safer alternatives are suggested, improving patient safety and optimizing treatment effectiveness.

Protein Modulation in Disease Treatment. The inhibitory or promotive effects of proteins on disease management are analyzed, providing insights into protein-drug interactions and supporting the design of effective therapeutic strategies.

Drug combination therapy. Drug combination therapies are analyzed to optimize dosages and interactions for treating complex conditions, ensuring maximum therapeutic benefits with minimal side effects.

Drug-Disease Treatment Relationships. Relationships between specific drugs and the diseases they treat are explored to gain insights into effective treatment options and potential new therapeutic applications for existing drugs.

Gene-Disease Associations. Comprehensive information on gene-disease associations reveals genetic predispositions, enabling the development of genetic tests for early detection and personalized treatments based on individual genetic profiles.

Dataset Recommendations by Research Direction. Metadata and literature are analyzed to identify and recommend datasets that align with specific research goals, providing high-quality, curated resources tailored to researchers' needs.

Methodology Recommendations by Research Direction. Suitable research methodologies are recommended based on the identified direction, equipping researchers with effective approaches ranging from traditional techniques to emerging methods.

Paper Recommendations by Research Direction. Relevant papers are suggested based on research direction, including seminal works, recent publications, and highly cited studies, enabling researchers to build on existing knowledge and stay updated on advancements in their field.

Dataset Inspection with LLM

Leveraging both unstructured and structured data, we utilize the capabilities of GPT-4o mini to generate a substantial corpus of over 5,000 question-answer pairs. To verify the reliability of the generated answers, we then employ GPT-4o. Given the original source articles, we design prompts for GPT-4o to identify the specific passage within the article that supports each QA pair. Dataset Inspection Prompt are detailed in Supporting Information Fig. S6.

Manual Inspection and Selection

To ensure the absolute reliability of the BioCDQA dataset, we implemented an exceptionally rigorous manual curation and selection protocol. This process was independently conducted by two authors with biomedical backgrounds, who filtered an initial pool of over 5,000 machine-generated QA pairs down to the final 1,183 high-quality entries. The reviewers evaluated each pair against a comprehensive set of criteria, including: Factual Correctness and Faithfulness, to ensure every assertion in the answer was clearly supported by and traceable to the source documents and free of

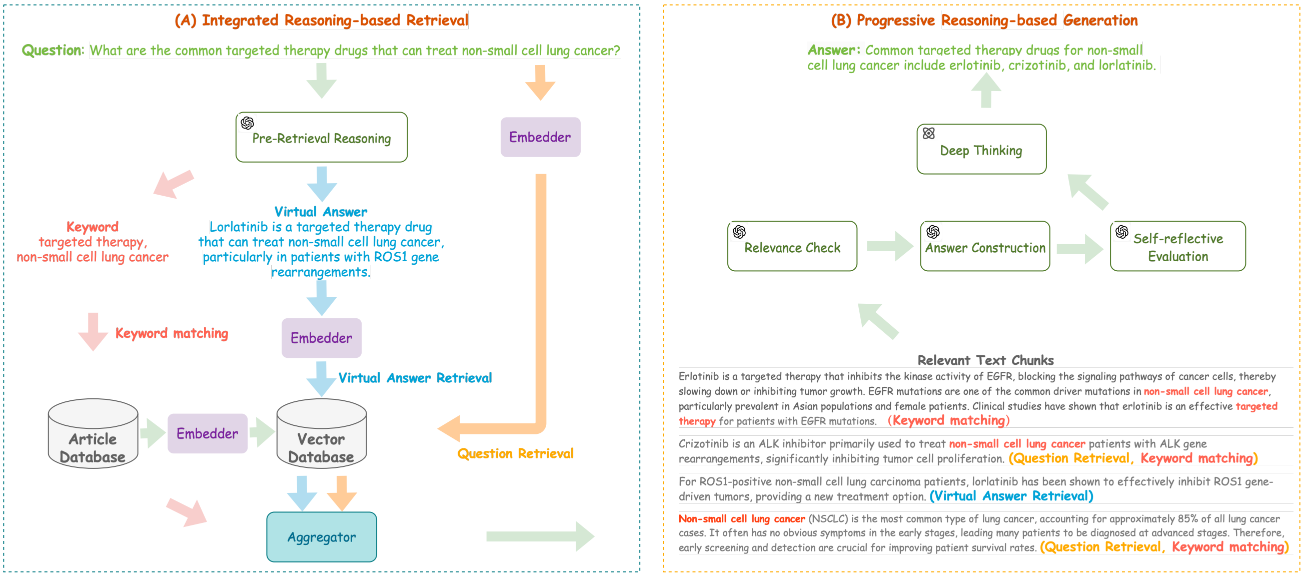

Figure 3. Framework of IP-RAR. (A) Integrated Reasoning-based Retrieval: Performs pre-retrieval reasoning, extracting keywords and generating a virtual answer. Then, a multi-level, multi-granularity retrieval strategy is used to retrieve relevant text chunks, which are ranked based on relevance. (B) Progressive Reasoning-based Generation: This stage begins with the Relevant Text Chunks retrieved from the process in (A). The labels next to each chunk (e.g., (Keyword matching), (Question Retrieval), (Virtual Answer Retrieval)) indicate which retrieval strategy sourced that piece of evidence.
Then, filters out irrelevant text chunks through explanations or self-reflection, then leverages DeepSeek-R1 for deep-thinking-based reasoning on the valid text chunks, generating a precise final response.

hallucinations; Completeness, to verify that no critical information was omitted; Relevance and Conciseness, to confirm the answer was focused and without redundant details; and the overall Question Quality (i.e., clarity and non-ambiguity). For the final selection, only those QA pairs that received consensus approval from both reviewers were retained. This high-quality dataset facilitates deep knowledge mining across a complex space of 68,428 research papers by providing contextually relevant questions linked to precise biomedical concepts for advanced reasoning.

Integrated and Progressive Retrieval-Augmented Reasoning Framework

In this study, we propose the IP-RAR framework, specifically designed for deep-thinking-based knowledge mining and question answering in large-scale biomedical articles. The overall framework is illustrated in Figure 3.

Integrated Reasoning-based Retrieval

Pre-Retrieval Reasoning. The first step employs the DeepSeek-V3 [31] to perform pre-retrieval reasoning by extracting key terms to identify relevant biomedical entities and generating synonyms to enhance matching flexibility. This approach overcomes the limitations of existing methods that struggle to precisely locate answers in complex queries, enabling more accurate targeting of specific biomedical entities. Furthermore, based on the pre-retrieval reasoning process, the system generates a virtual answer as a hypothesis to refine retrieval precision, improving the identification of relevant information. This improves the system's ability to link related paragraphs and retrieve contextually consistent information, with the prompt for pre-retrieval reasoning detailed in Supporting Information Fig. S7.

Multi-Level and Multi-Granularity Retrieval Strategy. To enhance recall and maximize the retrieval of relevant knowledge from millions of text chunks, we employ a retrieval strategy that integrates multi-level and multi-granularity approaches. The multi-level aspect combines abstract-based and full-text-based retrieval to capture relevant content across varying depths of detail. The multi-granularity aspect involves question-based, keyword-based, and virtual answer-based retrieval, ensuring a comprehensive exploration of the corpus. Using the Contriever-MS MARCO model [18], we first retrieve the top 10 abstracts based on the similarity between the question and text chunks, followed by another top 10 abstracts based on the similarity between the virtual answer and text chunks. At the full-text level, we retrieve the top 10 chunks for the question and an additional top 10 for the virtual answer using the same model. Additionally, we apply keyword-based matching techniques, expanding retrieval by including synonyms to increase flexibility. This multi-level and multi-granularity retrieval strategy effectively captures relevant content from different perspectives and depths, significantly improving the alignment of retrieved knowledge with the question's intent from a vast corpus.

Aggregator. The aggregator framework employs a weighted normalization function to rank retrieved chunks effectively. This function integrates three key factors: similarity score, method diversity, and intra-document repetition, ensuring contextually relevant and diverse rankings. Each factor is weighted according to its relevance, producing a normalized score S_i for each chunk, allowing for comparison across all retrieved chunks. The score for each chunk i is calculated as:

$$S_i = w_S \times \frac{S_{\text{sim},i}}{S_{\text{sim},max}} + w_M \times \frac{M_i}{M max} + w_R \times \frac{R_i}{R max} \quad (1)$$

where $S_{\text{sim},i}$ represents the similarity score of chunk i, M_i indicates the number of distinct retrieval methods that identified chunk i, and R_i reflects the count of retrieved chunks from the same document as chunk i. The terms $S_{\text{sim,max}}$, $M\text{max}$, and $R\text{max}$ are the maximum values of these metrics across all chunks, used for normalization. The weights w_S, w_M, and w_R control the importance of each factor, where w_S emphasizes the relevance of similarity scores, w_M highlights chunks retrieved by multiple methods, and w_R prioritizes chunks with higher intra-document retrieval coverage. The resulting normalized score S_i provides a robust ranking metric that integrates query relevance, retrieval diversity, and intra-document coverage, providing a comprehensive and balanced ranking metric. In our experiments, these weights are set to $w_S = 5$, $w_M = 3$, and

$w_R = 1$.

Progressive Reasoning-based Generation

The Progressive Reasoning-based Generation process in our proposed IP-RAR framework is designed to ensure that only the most relevant evidence is employed in generating the final response. This process unfolds in four distinct phases:

Relevance Check. In this phase, all candidate text chunks are ranked in descending order based on their retrieval scores. The ranked chunks are then sequentially presented to the DeepSeek-V3, which evaluates whether each chunk sufficiently answers the posed question. This process continues until 5 relevant chunks are identified. If fewer than 5 relevant chunks are available, only those identified as relevant are used; if no relevant chunks are identified, the top 5 highest-scoring chunks are selected regardless of their assessed relevance.

Answer Construction. After selecting the candidate text chunks, a simple prompt is constructed to allow the DeepSeek-V3 to generate an initial response to the question. The prompt incorporates the context of the question and the selected chunks to facilitate a coherent and informed answer.

Self-Reflective Evaluation. Following the generation of the response, a self-reflection evaluation is performed to systematically assess the degree to which each text chunk supports the generated response. The DeepSeek-V3 is prompted to evaluate the relevance of each chunk with respect to the question and the proposed answer, assigning a support score to each. Scores are given on a scale from 0 to 100, where 100 represents maximum relevance, and 0 indicates that the chunk is either entirely irrelevant or contradicts the proposed answer. Intermediate scores reflect varying levels of support based on the chunk's contribution to the response.

Deep Thinking. Based on the support scores assigned during the self-reflective evaluation, the system prioritizes the text chunks corresponding to the most relevant answers and utilizes DeepSeek-R1 [25] to perform deep-thinking-based reasoning for the final answer generation. The DeepSeek-R1 is prompted to integrate these highly relevant text chunks, producing a cohesive and precise final response to ensure both contextual consistency and comprehensive accuracy.

Results

Results of Various Approaches on the BioCDQA Dataset

For the BioCDQA dataset, we design a set of evaluation metrics. Document retrieval performance is assessed using Mean Precision, Mean Recall, and Mean F-measure to evaluate retrieval accuracy and completeness. Answer accuracy evaluation relies on a GPT-4-based five-point scoring system, ensuring a comprehensive assessment of the semantic consistency and precision of generated answers. This approach is particularly suitable for summary-type question-answering tasks, where traditional metrics struggle to provide stable quality measurements. A detailed introduction to the evaluation metrics can be found in the Supporting Information section 1.1.

As shown in Table 1, we evaluated IP-RAR against two distinct categories of methods: non-RAG and RAG-based. The non-RAG methods—Chain of Thought (CoT), Few-Shot, and MedAgents, all utilizing the DeepSeek-V3 model—rely solely on the LLM's internal, parametric knowledge. The results clearly demonstrate the limitations of this approach for our evidence-based, cross-document task; without the support of external data, all non-RAG methods achieve significantly lower answer scores. Among them, MedAgents performs best. This is likely because its multi-agent collaborative framework can more effectively extract the latent medical knowledge within the base LLM. However, its performance is still fundamentally capped by the lack of retrieved, context-specific evidence.

In contrast, RAG-based methods show a marked improvement. In the document retrieval stage, IP-RAR achieves the highest F1-score, demonstrating a superior balance between Precision and Recall. This high-fidelity retrieval is crucial for downstream performance. For instance, while SELF-RAG attains the highest recall, its extremely low precision indicates that it retrieves a large volume of irrelevant noise, which significantly hinders its answer generation performance. Similarly, CRAG's modest retrieval quality leads to a lower answer score. This highlights the importance of high-quality retrieval, which directly contributes to IP-RAR's state-of-the-art answer score of 76.41%. The overall results confirm that a RAG-based approach is essential for this task and that within this paradigm, IP-RAR's sophisticated architecture comprehensively outperforms all tested baseline systems.

In addition to retrieval and generation accuracy, we analyzed the inference time and GPU memory usage of the IP-RAR framework to evaluate its practical feasibility, conducting a direct comparison against the SELF-RAG baseline. The analysis was conducted on a single NVIDIA A100 GPU against our retrieval corpus of over 1.85 million document chunks. For a typical query, IP-RAR required an average of 26.7 seconds for a complete response, with a peak GPU memory usage of only 3GB. In contrast, SELF-RAG was slower, with an average time of 32.4 seconds, and required a substantially larger peak memory of 78GB. These results demonstrate that IP-RAR is not only faster but also orders of magnitude more resource-efficient. This comprehensive efficiency, especially when compared to the hours a human expert might need for manual synthesis, proves that the performance advantages of IP-RAR do not come at an impractical computational cost.

Performance of IP-RAR on Different Datasets

For the BioASQ and MASH-QA datasets, we adhere to the evaluation criteria established by their respective datasets.

BioASQ dataset

In our evaluation on the BioASQ dataset, IP-RAR is benchmarked against several top-performing systems from the challenge, which serve as robust baselines. The first baseline, dmiip3, combines BM25 and GPT-3.5 for initial retrieval, employs a cross-encoder re-ranker based on biomedical pretrained models like PubMedBERT, and finally generates answers through GPT-3.5. Another strong baseline, bioinfo-0, adopts a multi-stage pipeline: it begins with traditional BM25 retrieval, then enhances results using Transformer-based neural re-rankers such as PubMedBERT. For the BioASQ dataset, Table 2(a) demonstrates the superior document retrieval performance of IP-RAR. With higher precision and F1-score, IP-RAR effectively filters out irrelevant documents, while its leading performance in MAP and GMAP highlights its strength in ranking relevant documents. In contrast, although SELF-RAG achieves a higher recall, IP-RAR's precision-focused trade-off results in better overall retrieval quality. Methods like bioinfo-0 and dmiip3 improve recall but perform worse in precision and ranking, often retrieving excessive irrelevant content.

It is noteworthy that both IP-RAR and SELF-RAG employ similar workflows: an ideal answer is first generated through their respective systems, followed by refinement using DeepSeek-V3 to extract the final response tailored to the specific question type. For these two systems, all evaluations are conducted on the extracted answers to ensure consistency and comparability. Other methods, such as dmiip3 and BioASQ Baseline, are evaluated based on their original outputs to maintain fairness and a consistent basis for comparison. Table 2(b) presents a comparison of exact answer results across IP-RAR and other methods. For Yes/No questions, IP-RAR achieves an F1 score and accuracy close to 96%, second only to IISR-2. In Factoid questions, it ranks first with a Strict Accuracy of 68.36%

System	Document Retrieval			Answer
	Prec. (%)	Rec. (%)	F1 (%)	GPT-4 Eval (%)
**Non-RAG Methods**				
CoT	/	/	/	21.83
Few-Shot	/	/	/	15.62
MedAgents	/	/	/	38.89
**RAG-based Methods**				
Contriever+Llama 3 8B	24.53	19.40	21.67	25.86
CRAG	12.18	18.49	14.69	29.27
SELF-RAG	7.12	**31.28**	11.59	41.16
**IP-RAR (ours)**	**47.18**	27.76	**34.96**	**76.41**

Table 1. Comparison of IP-RAR against non-RAG and RAG-based methods on the BioCDQA dataset. The best results are highlighted in bold.

System	P (%)	R (%)	F1 (%)	MAP (%)	GMAP (%)
A&Q4	10.27	58.16	17.46	44.04	2.15
dmiip3	11.33	61.27	19.12	44.62	2.40
bioinfo-0	21.18	60.47	31.37	45.90	2.67
SELF-RAG	49.82	**74.53**	59.72	89.33	22.15
**IP-RAR**	**85.87**	57.95	**69.20**	**95.12**	**35.66**

(a)

System	Yes/No		Factoid		List		
	F1	Acc.	Str. Acc.	MRR	Prec.	Rec.	F1
Baseline	60.00	46.67	9.09	11.36	11.85	27.84	16.13
dmiip3	85.71	87.30	31.82	39.92	28.51	24.64	22.32
UR-gpt4	94.74	95.64	54.55	56.82	37.42	43.69	38.28
IISR-2	**100.00**	**100.00**	54.55	59.09	50.99	35.77	39.80
SELF-RAG	85.14	82.55	30.61	28.31	51.10	22.38	29.19
**IP-RAR**	95.91	95.34	**68.36**	**67.34**	**88.78**	**54.53**	**63.04**

(b)

Table 2. Comparison of document retrieval and exact answer results between IP-RAR and commonly used methods on the BioASQ dataset. (a) Document retrieval performance comparison. (b) Exact answer generation performance comparison. The best results are highlighted in bold.

and an MRR of 67.34%. For List questions, IP-RAR outperforms all systems with the highest Precision, Recall, and F1, demonstrating superior retrieval completeness.

In summary, IP-RAR outperforms other methods on the BioASQ dataset in both document retrieval and exact answer tasks, demonstrating superior precision, recall, ranking, and comprehensiveness.

MASH-QA dataset

Table 3 highlights IP-RAR's superior performance on the MASH-QA dataset. For sentence retrieval, it achieves the highest F1 score (64.44%) with a strong balance of Precision and Recall, outperforming SELF-RAG, which suffers from low Precision (27.46%) despite its high Recall.

In answer prediction, IP-RAR's Exact Match (47.29%) more than doubles that of MultiCo, demonstrating superior accuracy. Notably, since both IP-RAR and SELF-RAG rely on LLMs to generate answers, the EM metric was evaluated using a GPT-4o-based [32] scoring system, which assigns a score of 1 only when the predicted answer and the gold standard convey the exact same meaning without any extraneous sentences. Any discrepancy in meaning or the inclusion of irrelevant sentences results in a score of 0. This strict scoring criterion highlights IP-RAR's ability to produce concise and semantically accurate answers.

Compared to baseline models like BERT, RoBERTa, and XLNet, which exhibit low EM scores (below 10%), IP-RAR excels in aligning retrieved sentences with precise answers.

Model name	Sentence			Answer
	P	R	F1	EM
TANDA	56.48	16.42	25.44	8.95
BERT	56.18	16.25	25.21	8.89
RoBERTa	57.70	19.06	28.65	9.40
XLNet	56.05	19.73	29.19	9.09
MultiCo	58.16	55.90	57.00	22.05
SELF-RAG	27.46	**82.84**	41.26	12.96
**IP-RAR(ours)**	**60.95**	68.35	**64.44**	**47.29**

Table 3. Comparison of preliminary sentence retrieval results between IP-RAR and commonly used methods on the MASH-QA dataset. The best results are highlighted in bold.

Analysis of the Recall for Multi-Level and Multi-Granularity Retrieval Strategy

Table 4 presents the recall (%) results of the Multi-Level and Multi-Granularity Retrieval Strategy and its ablation variants on the BioCDQA dataset. From the multi-level perspective, combining abstract-level and full-text-level retrieval ensures a more comprehensive capture of relevant information, covering knowledge sources from concise to detailed. Multi-level retrieval improves re-

Method	Hierarchical Retrieval			Multi-Granularity Retrieval	
	Abstract	Full-Text	Multi-Level	Value	Strategy Composition
Question-based	36.80	31.28	40.04	40.04	Only Question-based
Virtual Answer-based	34.79	27.63	39.62	44.06	+ Virtual Answer-based
Keyword-based	4.10	42.46	42.46	66.10	+ Keyword-based

Table 4. Recall (%) for Multi-Level and Multi-Granularity Retrieval Strategy.

call by 8.76% and 11.99% for Question-based Retrieval and Virtual Answer-based Retrieval, respectively, demonstrating that single-level retrieval alone is insufficient to achieve full coverage. By integrating results across levels, multi-level retrieval significantly enhances performance. However, for Keyword-based Retrieval, multi-level integration shows limited improvement, as keyword-based methods struggle to retrieve meaningful information at the abstract level. This indicates that keywords are less effective in abstracts, but their strong performance at the full-text level compensates for this limitation.

From the multi-granularity perspective, the strategy combines question-based, virtual answer-based, and keyword-based retrieval to leverage different representations of relevance. The ablation results demonstrate that each granularity contributes effectively to the final outcome. Question-based retrieval achieves a recall of 40.04%, while virtual answer-based retrieval further improves it to 44.06%. Keyword-based retrieval, particularly with synonym expansion, significantly boosts recall to 66.10%. This highlights that keyword-based retrieval complements the other granularities by capturing additional variations in linguistic expression. Importantly, each granularity contributes meaningfully, and their combined strengths play a crucial role in achieving the highest recall.

In summary, the integration of multi-level and multi-granularity strategies ensures the retrieval process captures relevant content comprehensively and effectively. The synergy between these dimensions is critical for achieving high recall, especially in large-scale datasets like BioCDQA, where maximizing coverage is essential for downstream applications.

Impact of IP-RAR Components on Performance

The ablation study presented in Table 5 provides insights into the contributions of various components in the IP-RAR framework under the DeepSeek-V3. By isolating individual components, the analysis demonstrates how each affects the overall system's ability to generate accurate and contextually appropriate answers.

In the ablation study, *w/o* Retrieval examines the system's performance when bypassing document retrieval entirely, relying solely on the DeepSeek-V3 to generate answers. In this configuration, the Precision, Recall, and F1 score metrics are not applicable, as no documents are retrieved. This setup leads to a significant performance drop, with a GPT-4 evaluation score of only 37.12%. These results underscore the vital role of the retrieval process in grounding the model's answers with relevant context. Without retrieval, the system lacks access to supporting information, resulting in vague or less accurate answers.

For *w/o* Integrated Reasoning-based Retrieval, the system bypasses the multi-level and multi-granularity retrieval strategy, directly retrieving a flat set of the top 50 text chunks using Contriever-MS MARCO and ranking the top 5 chunks during the progressive reasoning-based generation process. In this configuration, the Precision is 27.95%, Recall is 18.04%, and F1 score is 21.29%. This results in a GPT-4 evaluation score of 50.18%, reflecting the importance of the multi-level and multi-granularity retrieval strategy in refining the retrieval of highly relevant text chunks. While this alternative method retrieves some relevant documents, the overall recall remains too low to support comprehensive and accurate answer generation.

The *w/o* Progressive Reasoning-based Generation ablation removes the Progressive Reasoning-based Generation module and generates answers directly from the content retrieved by the Integrated reasoning-based Retrieval module. In this setup, the Precision drops to 16.13%, Recall increases to 32.81%, and F1 score decreases to 21.62%. Without the Progressive Reasoning-based Generation process, the system generates answers based on the top 5 chunks identified by the recall process, resulting in a GPT-4 evaluation score of 52.36%. Experimental results show that retrieving too much irrelevant information either introduces large amounts of invalid knowledge that disrupts the generation process or causes the context to exceed the token limit of the LLM, leading to the removal of potentially crucial information. The complete IP-RAR framework, with all components integrated, achieves the highest performance, with a GPT-4 evaluation score of 76.41%. Specifically, the Precision is 47.18%, Recall is 27.76%, and F1 score is 34.96%. This result underscores the importance of each component in ensuring the system's ability to deliver precise and contextually grounded answers. Removing any component results in a significant performance degradation, affirming that retrieval, integrated reasoning-based retrieval, and progressive reasoning-based generation are all essential for achieving optimal results in biomedical question answering.

Formulating Scientific Questions and Planning Research

Formulating scientific questions and designing research plans are critical steps in driving innovation and breakthroughs in biomedical research. A well-defined and challenging scientific question not only determines the direction of the study but also influences data collection, selection of experimental methods, and the interpretability of research outcomes. A well-structured research plan, in turn, optimizes resource allocation and enhances both feasibility and impact. By efficiently leveraging existing articles to identify research gaps and propose novel scientific hypotheses, researchers can significantly improve research efficiency. These hypotheses not only provide a solid theoretical foundation for subsequent experiments or clinical studies but also accelerate the understanding of disease mechanisms and the development of novel therapeutic strategies. Systematic analysis and integration of articles enable researchers to uncover unknown aspects of disease biology or limitations in current therapeutic approaches, thereby improving the rationality of hypotheses and the relevance of experimental designs. This process is crucial for drug development and the optimization of precision medicine strategies, ultimately expediting scientific discovery [33, 34].

Our framework facilitates the efficient extraction of key insights from existing article, generating scientific hypotheses and designing research plans. Figure 4a illustrates the systematic process of retrieval, integration, and reasoning, which ultimately generates evidence-based scientific questions to guide subsequent experimental designs. For instance, miR-375 may play distinct roles in the progression of different colorectal cancer subtypes, such as adenocarcinoma, squamous cell carcinoma, and small cell lung cancer. Its target gene, ITPKB, may be directly regulated by miR-375 and play a crucial role in cancer development. Based on this hypothesis, researchers can conduct targeted in vitro experiments,

System	Document Retrieval			Answer
	Prec. (%)	Rec. (%)	F1 (%)	GPT-4 Eval (%)
IP-RAR(ours)	47.18	27.76	34.96	76.41
w/o Progressive Reasoning-based Generation	16.13	32.81	21.62	52.36
w/o Integrated Reasoning-based Retrieval	27.95	18.04	21.29	50.18
w/o Retrieval	/	/	/	37.12

Table 5. Ablation study of the IP-RAR framework

such as luciferase reporter assays and Western blot analysis, to validate the regulation of ITPKB by miR-375. Additionally, in vivo studies using mouse models can effectively evaluate the impact of miR-375 on tumor growth and metastasis across different cancer subtypes. Further, large-scale high-throughput analyses of miR-375 expression patterns and its target genes in colorectal cancer patient cohorts, combined with correlations to clinical outcomes, can provide actionable research insights. This structured approach not only guides experimental design but also optimizes research workflows, enhancing both rigor and efficiency. By leveraging this literature-driven research methodology, researchers can accelerate scientific discoveries and improve the planning and execution of biomedical studies.

Drug Interaction Research in Clinical Decision Support

Research on drug interactions plays a crucial role in clinical decision support, ensuring treatment safety and efficacy, particularly in the context of polypharmacy and personalized medicine [35]. Many patients require multiple medications to manage comorbidities, making a deep understanding of drug interactions essential for clinicians to identify potential risks, such as enhanced side effects, altered drug metabolism, or reduced therapeutic effectiveness. Leveraging our approach, clinicians can more efficiently uncover synergistic effects that enhance therapeutic outcomes or identify antagonistic interactions that compromise efficacy [36]. This process not only aids in preventing adverse reactions but also supports the development of safer and more effective treatment plans, especially for elderly patients and those with multiple comorbid conditions.

For patients with coexisting lymphoma and colorectal cancer, robust clinical decision support is essential for optimizing treatment plans and reducing the risks associated with drug interactions. Figure 4b illustrates the system's retrieval and reasoning process, ultimately generating evidence-based answers. IP-RAR first performs pre-retrieval reasoning using a large language model, extracting keywords from the query and generating an initial response. It then retrieves relevant medical articles from databases to extract supporting evidence, including the facts that Cisplatin may induce resistance in lymphoma and that it has an antagonistic interaction with Cetuximab. The system then applies self-reflection to determine which text chunks are relevant to the question, followed by deep thinking to infer drug interactions. Finally, it recommends avoiding Cisplatin and suggests Carboplatin as an alternative therapy. This evidence-based reasoning approach assists clinicians in developing safer and more effective treatment plans, thereby enhancing patient safety and improving outcomes for individuals with complex, multi-disease conditions.

Discussion

To advance biomedical knowledge extraction and application, this paper proposes a comprehensive framework that integrates knowledge graphs with LLMs. By constructing the BioStrataKG from large-scale biomedical articles, the framework systematically uncovers multi-layered relationships among biomedical entities, such as genes, proteins, diseases, and drugs, as well as interactions between research papers in terms of methodologies, datasets, research directions, and citation relationships. To support cross-document reasoning and biomedical knowledge mining, we develop the BioCDQA dataset based on BioStrataKG, addressing the limitations of existing biomedical QA datasets in handling cross-document and high-level reasoning tasks. Furthermore, we introduce the IP-RAR framework, which combines Integrated Reasoning-based Retrieval with Progressive Reasoning-based Generation, enabling LLMs to efficiently retrieve, synthesize, and utilize multi-source evidence.

Experimental results demonstrate that IP-RAR significantly outperforms previous approaches in both retrieval efficiency and answer accuracy. In clinical applications, the framework assists physicians in rapidly identifying and synthesizing critical information from vast biomedical articles, facilitating the development of more precise personalized treatment plans. In research settings, it enables systematic analysis of cutting-edge advancements and identification of potential research gaps, accelerating research strategy formulation and decision-making. IP-RAR holds potential in various biomedical domains, including assisting in drug synergy/antagonism analysis, generating hypotheses for drug repurposing, and providing evidence synthesis for precision medicine, providing essential technical support for advancing biomedical research and clinical practice.

Despite its promising results, IP-RAR still faces challenges in handling highly complex multimodal data, dynamically evolving scientific knowledge, and human-AI interaction. Future work will explore multimodal information integration and agent-based interactions to further enhance the robustness and adaptability of the framework, ensuring more comprehensive support for biomedical knowledge mining and clinical applications. By continuously improving the dynamic maintenance of knowledge graphs and refining reasoning mechanisms, the proposed framework is expected to establish a more efficient and precise paradigm for biomedical knowledge discovery, fostering rapid advancements in scientific research and clinical decision-making.

Availability of Source Code and Requirements

Project name: BioCDQA

- Project homepage: https://github.com/yichun10/BioCDQA.git.
- Operating system(s): Linux (Ubuntu)
- Programming language: Shell Script (Bash) with Python 3.10.13
- Other requirements: Python 3.10.13 with GPU/CPU support, neo4j 5.13.0 (please see more requirements on Github repository).
- Licenses: MIT license
- Research Resource Identifier (#RRID): SCR_027068
- Computational Workflow DOI: https://doi.org/10.48546/WORKFLOWHUB.WORKFLOW.1744.1

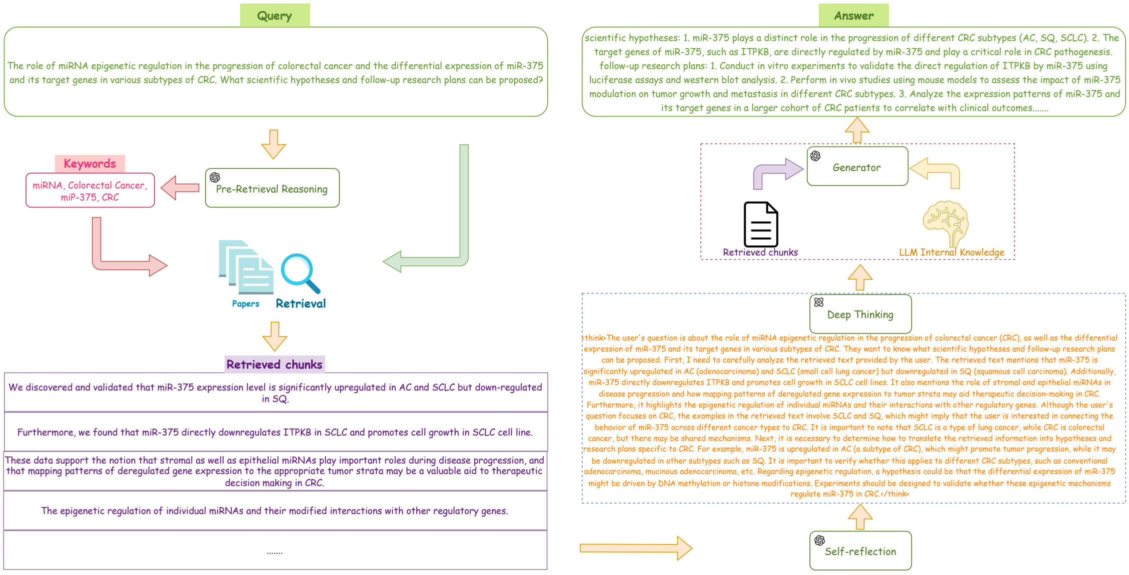

(a)

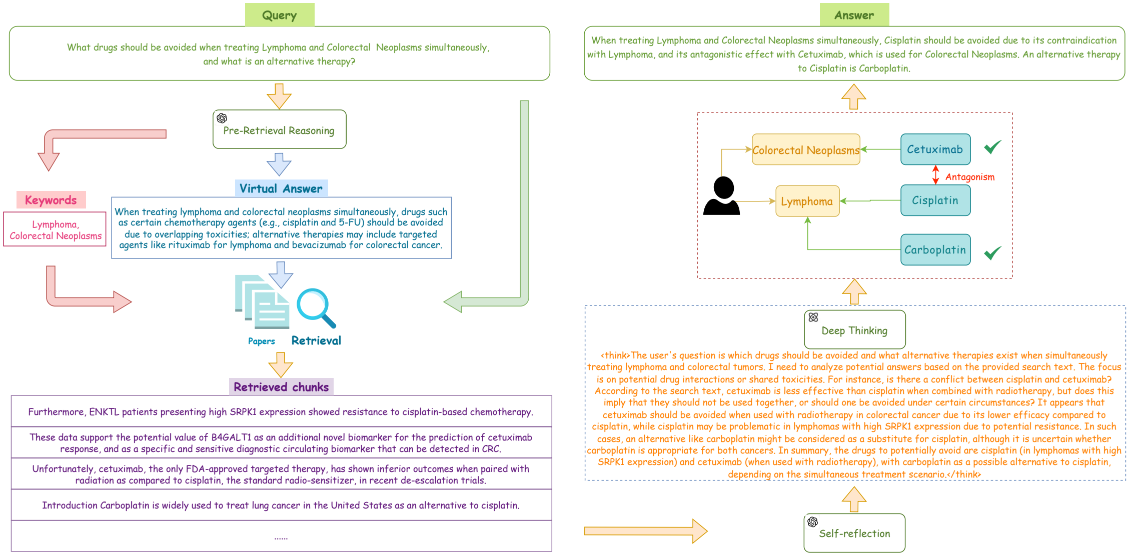

(b)

Figure 4. Examples of Applications in Biomedical Research and Clinical Decision Support. (a) An Example of Formulating Scientific Questions and Planning Research. (b) An Example of Drug Interaction Research for Clinical Decision Support.

Data Availability

The codes and datasets are available for access at https://github.com/yichun10/BioCDQA.git.

Supplementary material

Supplementary material is available at Supplementary material.pdf.

Abbreviations

KG: knowledge graph; LLM: large language model; NLP: natural language processing; QA: question answering; RAG: Retrieval-Augmented Generation; IP-RAR: Integrated and Progressive Retrieval-Augmented Reasoning; GPT: generative pre-trained transformer.

Competing Interests

No competing interest is declared.

Funding

This work was supported in part by funds from the National Key R&D Program (No. 2022YFF1202101, 2023YFF1204701); the CAS Research Fund (No. XDB38050200); the Self-supporting Program of Guangzhou National Laboratory (No. SRPG22007); the Startup Program of Guangzhou National Laboratory (No. YW-YFYJ0101).

Authors' Contributions

Y.F. and L.Z. performed conceptualization and investigation; Y.F. performed data curation, formal analysis, methodology, and original draft writing; Y.L. and L.Z. performed resources and supervision; Y.F. , J.W., L.Z., R.H., and Y.L. reviewed and edited the manuscript.

References

1. Omiye JA, Gui H, Rezaei SJ, Zou J, Daneshjou R. Large language models in medicine: the potentials and pitfalls: a narrative review. Annals of internal medicine 2024;177(2):210–220.
2. Liu T, Zheng X, Chang B, Sui Z. Towards faithfulness in open domain table-to-text generation from an entity-centric view. In: Proceedings of the AAAI Conference on Artificial Intelligence, vol. 35; 2021. p. 13415–13423.
3. Comeau DC, Islamaj Doğan R, Ciccarese P, Cohen KB, Krallinger M, Leitner F, et al. BioC: a minimalist approach to interoperability for biomedical text processing. Database (Oxford) 2013;2013:bat064.
4. Doan S, Conway M, Phuong TM, Ohno-Machado L. Natural language processing in biomedicine: a unified system architecture overview. Clinical bioinformatics 2014;p. 275–294.
5. Cai L, Li J, Lv H, Liu W, Niu H, Wang Z. Integrating domain knowledge for biomedical text analysis into deep learning: A survey. Journal of Biomedical Informatics 2023;143:104418.
6. Chang D, Balažević I, Allen C, Chawla D, Brandt C, Taylor RA. Benchmark and best practices for biomedical knowledge graph embeddings. In: Proceedings of the conference. Association for Computational Linguistics. Meeting, vol. 2020; 2020. p. 167.
7. Li L, Wang P, Yan J, Wang Y, Li S, Jiang J, et al. Real-world data medical knowledge graph: construction and applications. Artificial intelligence in medicine 2020;103:101817.
8. Zheng S, Rao J, Song Y, Zhang J, Xiao X, Fang EF, et al. PharmKG: a dedicated knowledge graph benchmark for bomedical data mining. Briefings in bioinformatics 2021;22(4):bbaa344.
9. Wu X, Duan J, Pan Y, Li M. Medical knowledge graph: Data sources, construction, reasoning, and applications. Big Data Mining and Analytics 2023;6(2):201–217.
10. Zhu M, Ahuja A, Juan DC, Wei W, Reddy CK. Question answering with long multiple-span answers. In: Findings of the Association for Computational Linguistics: EMNLP 2020; 2020. p. 3840–3849.
11. Nentidis A, Katsimpras G, Krithara A, Lima López S, Farré-Maduell E, Gasco L, et al. Overview of bioasq 2023: The eleventh bioasq challenge on large-scale biomedical semantic indexing and question answering. In: International Conference of the Cross-Language Evaluation Forum for European Languages Springer; 2023. p. 227–250.
12. Welbl J, Stenetorp P, Riedel S. Constructing datasets for multi-hop reading comprehension across documents. Transactions of the Association for Computational Linguistics 2018;6:287–302.
13. Abacha AB, Mrabet Y, Sharp M, Goodwin TR, Shooshan SE, Demner-Fushman D. Bridging the gap between consumers' medication questions and trusted answers. In: MEDINFO 2019: Health and Wellbeing e-Networks for All IOS Press; 2019.p. 25–29.
14. Pal A, Umapathi LK, Sankarasubbu M. Medmcqa: A large-scale multi-subject multi-choice dataset for medical domain question answering. In: Conference on health, inference, and learning PMLR; 2022. p. 248–260.
15. Feng Y, Zhou L, Ma C, Zheng Y, He R, Li Y. Knowledge Graph-based Thought: a knowledge graph enhanced LLMs framework for pan-cancer question answering. GigaScience 2024;Accepted for publication, to appear.
16. Lewis P, Perez E, Piktus A, Petroni F, Karpukhin V, Goyal N, et al. Retrieval-augmented generation for knowledge-intensive nlp tasks. Advances in Neural Information Processing Systems 2020;33:9459–9474.
17. Jegal Y, Choi J, Lee J, Park KS, Lee S, Yoon J. Learning a Patent-Informed Biomedical Knowledge Graph Reveals Technological Potential of Drug Repositioning Candidates. arXiv preprint arXiv:230903227 2023;.
18. Izacard G, Caron M, Hosseini L, Riedel S, Bojanowski P, Joulin A, et al. Unsupervised dense information retrieval with contrastive learning. arXiv preprint arXiv:211209118 2021;.
19. Asai A, Wu Z, Wang Y, Sil A, Hajishirzi H. Self-rag: Learning to retrieve, generate, and critique through self-reflection. arXiv preprint arXiv:231011511 2023;.
20. Edge D, Trinh H, Cheng N, Bradley J, Chao A, Mody A, et al. From local to global: A graph rag approach to query-focused summarization. arXiv preprint arXiv:240416130 2024;.
21. Yan SQ, Gu JC, Zhu Y, Ling ZH. Corrective retrieval augmented generation. arXiv preprint arXiv:240115884 2024;.
22. Sarthi P, Abdullah S, Tuli A, Khanna S, Goldie A, Manning CD. Raptor: Recursive abstractive processing for tree-organized retrieval. arXiv preprint arXiv:240118059 2024;.
23. Tang X, Zou A, Zhang Z, Li Z, Zhao Y, Zhang X, et al. MedAgents: Large Language Models as Collaborators for Zero-shot Medical Reasoning. In: Findings of the Association for Computational Linguistics ACL 2024; 2024. p. 599–621.
24. Jaech A, Kalai A, Lerer A, Richardson A, El-Kishky A, Low A, et al. Openai o1 system card. arXiv preprint arXiv:241216720 2024;.
25. Guo D, Yang D, Zhang H, Song J, Zhang R, Xu R, et al. Deepseek-r1: Incentivizing reasoning capability in llms via reinforcement learning. arXiv preprint arXiv:250112948 2025;.
26. OpenAI, GPT-4o mini: advancing cost-efficient intelligence; 2024. https://openai.com/index/gpt-4o-mini-advancing-cost-efficient-intelligence/.
27. Paruchuri V, Marker;. https://github.com/VikParuchuri/marker.

28. National Library of Medicine, Medical Subject Headings (MeSH); 2023. `https://www.nlm.nih.gov/mesh/meshhome.html`.
29. UniProt: the universal protein knowledgebase in 2023. Nucleic acids research 2023;51(D1):D523–D531.
30. Wang W, Wei F, Dong L, Bao H, Yang N, Zhou M. Minilm: Deep self-attention distillation for task-agnostic compression of pre-trained transformers. Advances in Neural Information Processing Systems 2020;33:5776–5788.
31. Liu A, Feng B, Xue B, Wang B, Wu B, Lu C, et al. Deepseek-v3 technical report. arXiv preprint arXiv:241219437 2024;.
32. Hurst A, Lerer A, Goucher AP, Perelman A, Ramesh A, Clark A, et al. Gpt-4o system card. arXiv preprint arXiv:241021276 2024;.
33. Rajpal DK, Qu XA, Freudenberg JM, Kumar VD. Mining emerging biomedical literature for understanding disease associations in drug discovery. Biomedical Literature Mining 2014;p. 171–206.
34. Mohs RC, Greig NH. Drug discovery and development: Role of basic biological research. Alzheimer's & Dementia: Translational Research & Clinical Interventions 2017;3(4):651–657.
35. Maher RL, Hanlon J, Hajjar ER. Clinical consequences of polypharmacy in elderly. Expert opinion on drug safety 2014;13(1):57–65.
36. Neuvonen PJ, Niemi M, Backman JT. Drug interactions with lipid-lowering drugs: mechanisms and clinical relevance. Clinical Pharmacology & Therapeutics 2006;80(6):565–581.

Click here to access/download
Supplementary Material
Revision_highlight_giga_234.pdf

Supplementary Material

Click here to access/download
Supplementary Material
Supporting Information.pdf

Click here to access/download
Supplementary Material
Revision Notes for GIGA-D-25-00234.docx
