# Supplementary material for: A retrieval-augmented knowledge mining method with deep thinking LLMs for biomedical research and clinical support
Source: Gigascience. 2025 Sep 17;14:giaf109. doi: 10.1093/gigascience/giaf109 (PMC12448786; doi:10.1093/gigascience/giaf109)
Supplement: giaf109_GIGA-D-25-00234_Revision_3 [file giaf109_giga-d-25-00234_revision_3.pdf]

GigaScience

A Retrieval-Augmented Knowledge Mining Method with Deep Thinking LLMs for Biomedical Research and Clinical Support

--Manuscript Draft--

**Manuscript Number:**	GIGA-D-25-00234R3
**Full Title:**	A Retrieval-Augmented Knowledge Mining Method with Deep Thinking LLMs for Biomedical Research and Clinical Support
**Article Type:**	Research
**Funding Information:**	National Key R&D Program (No. 2022YFF1202101, 2023YFF1204701) — Ph.D. Yixue Li<br>the CAS Research Fund (No. XDB38050200) — Ph.D. Yixue Li<br>the Self-supporting Program of Guangzhou National Laboratory (No. SRPG22007) — Ph.D. Yixue Li<br>the Startup Program of Guangzhou National Laboratory (No. YW-YFYJ0101) — Ph.D. Yixue Li
**Abstract:**	Background. Knowledge graphs and large language models (LLMs) are key tools for biomedical knowledge integration and reasoning, facilitating structured organization of scientific articles and discovery of complex semantic relationships. However, current methods face challenges: knowledge graph construction is limited by complex terminology, data heterogeneity, and rapid knowledge evolution, while LLMs show limitations in retrieval and reasoning, making it difficult to uncover cross-document associations and reasoning pathways.<br>Results. We propose a pipeline that uses LLMs to construct a biomedical knowledge graph (BioStrataKG) from large-scale articles and builds a cross-document question-answering dataset (BioCDQA) to evaluate latent knowledge retrieval and multi-hop reasoning. We then introduce Integrated and Progressive Retrieval-Augmented Reasoning (IP-RAR) to enhance retrieval accuracy and knowledge reasoning. IP-RAR maximizes information recall through Integrated Reasoning-based Retrieval and refines knowledge via Progressive Reasoning-based Generation, using self-reflection to achieve deep thinking and precise contextual understanding. Experiments show that IP-RAR improves document retrieval F1 score by 20% and answer generation accuracy by 25% over existing methods.<br>Conclusions. The IP-RAR helps doctors efficiently integrate treatment evidence to inform the development of personalized medication plans and enables researchers to analyze advancements and research gaps, accelerating the hypothesis generation phase of scientific discovery and decision-making.
**Corresponding Author:**	Yichun Feng<br>University of the Chinese Academy of Sciences<br>Hangzhou, CHINA
**Corresponding Author Secondary Information:**	
**Corresponding Author's Institution:**	University of the Chinese Academy of Sciences
**Corresponding Author's Secondary Institution:**	
**First Author:**	Yichun Feng
**First Author Secondary Information:**	
**Order of Authors:**	Yichun Feng<br>Jiawei Wang<br>Ruikun He

Powered by Editorial Manager® and ProduXion Manager® from Aries Systems Corporation

	Lu Zhou
	Yixue Li
**Order of Authors Secondary Information:**	
**Response to Reviewers:**	Response to EIC:<br><br>#Q1: Corresponding Author Clarification<br><br># Response: To clarify, Dr. Lu Zhou and Dr. Yixue Li have been clearly indicated as the corresponding authors at all stages of manuscript preparation and submission. I was listed as the corresponding author in the submission system purely for administrative convenience and to facilitate communication during the submission process. In the manuscript itself, from the initial submission to the final accepted version, Dr. Zhou and Dr. Li were consistently marked as the corresponding authors.<br><br>According to Gigascience's guidelines, corresponding authors must be readily available throughout the submission and peer-review process to respond to editorial queries promptly. They are also expected to address post-publication critiques and comply with requests for additional data or information to address any issues that may arise after publication. If an author's seniority makes them too busy to fulfill this role effectively, it is important to ensure that the designated corresponding authors can respond promptly. Busy researchers often face delays due to travel, institutional responsibilities, or high email volumes, which can significantly hinder the publication process. This is why I acted as the corresponding author in the submission system—to streamline communication and ensure the timely handling of relevant matters.<br><br>#Q2: Data Availability Statement<br><br># Response: We have updated the Data Availability section with the following statement: "The codes and datasets are openly available in the GigaScience repository, GigaDB [REF#], and have been formally cited in the References list.<br><br>#Q3: Citations Update<br><br># Response: All arXiv references with published versions have been updated to their official journal citations. References not yet published in peer-reviewed journals or conferences remain in their preprint versions.
**Additional Information:**	
**Question**	**Response**
Are you submitting this manuscript to a special series or article collection?	No
**Experimental design and statistics**<br><br>Full details of the experimental design and statistical methods used should be given in the Methods section, as detailed in our Minimum Standards Reporting Checklist. Information essential to interpreting the data presented should be made available in the figure legends.<br><br>Have you included all the information requested in your manuscript?	Yes

**Resources**<br><br>A description of all resources used, including antibodies, cell lines, animals and software tools, with enough information to allow them to be uniquely identified, should be included in the Methods section. Authors are strongly encouraged to cite Research Resource Identifiers (RRIDs) for antibodies, model organisms and tools, where possible.<br><br>Have you included the information requested as detailed in our Minimum Standards Reporting Checklist?	Yes
**Availability of data and materials**<br><br>All datasets and code on which the conclusions of the paper rely must be either included in your submission or deposited in publicly available repositories (where available and ethically appropriate), referencing such data using a unique identifier in the references and in the "Availability of Data and Materials" section of your manuscript.<br><br>Have you have met the above requirement as detailed in our Minimum Standards Reporting Checklist?	Yes
GigaScience has policies and guidelines in place for the use of generative AI-writing tools such as ChatGPT. If you have used such writing tools to assist with writing the manuscript this must be declared and cited in the text. Authors should not list AI-writing tools and other AI-assisted technologies as an author or co-author and should acknowledge that they are fully responsible for text generated or refined by AI-writing tools.<p><br><br>A summary of use (particularly in the introduction or among methods) needs to be included at the end of the paper, and the outputs should also be included as a supplementary file hosted in GigaDB or other open repositories. Please <a	No

href=https://academic.oup.com/gigascience/pages/editorial_policies_and_reporting_standards target="_new" > read our guidelines for more information. <p><br><br>By submitting to GigaScience, you are aware of the journal's AI-writing tools policy, and if you have declared use of such tools below, you have acknowledged this where appropriate in your manuscript and have made a summary of use and outputs available. <p><br><b>AI-assisted writing tools have been used in the preparation of this manuscript?	

Powered by Editorial Manager® and ProduXion Manager® from Aries Systems Corporation

Click here to access/download;LaTeX - Main Document (TeX file);main.tex

```
This is pdfTeX, Version 3.141592653-2.6-1.40.26 (TeX Live 2024)
(preloaded format=pdflatex 2024.8.2)  21 AUG 2025 00:26
entering extended mode
 restricted \write18 enabled.
 %&-line parsing enabled.
**main.tex
(./main.tex
LaTeX2e <2024-06-01> patch level 2
L3 programming layer <2024-05-27>
(./oup-contemporary.cls
Document Class: oup-contemporary 2023/06/12, v1.2
(c:/texlive/2024/texmf-dist/tex/latex/base/article.cls
Document Class: article 2024/02/08 v1.4n Standard LaTeX document class
(c:/texlive/2024/texmf-dist/tex/latex/base/size10.clo
File: size10.clo 2024/02/08 v1.4n Standard LaTeX file (size option)
)
\c@part=\count194
\c@section=\count195
\c@subsection=\count196
\c@subsubsection=\count197
\c@paragraph=\count198
\c@subparagraph=\count199
\c@figure=\count266
\c@table=\count267
\abovecaptionskip=\skip49
\belowcaptionskip=\skip50
\bibindent=\dimen141
) (c:/texlive/2024/texmf-dist/tex/latex/base/inputenc.sty
Package: inputenc 2024/02/08 v1.3d Input encoding file
\inpenc@prehook=\toks17
\inpenc@posthook=\toks18
) (c:/texlive/2024/texmf-dist/tex/latex/base/fontenc.sty
Package: fontenc 2021/04/29 v2.0v Standard LaTeX package
) (c:/texlive/2024/texmf-dist/tex/generic/iftex/ifpdf.sty
Package: ifpdf 2019/10/25 v3.4 ifpdf legacy package. Use iftex instead.
(c:/texlive/2024/texmf-dist/tex/generic/iftex/iftex.sty
Package: iftex 2022/02/03 v1.0f TeX engine tests
)) (c:/texlive/2024/texmf-dist/tex/latex/microtype/microtype.sty
Package: microtype 2024/03/29 v3.1b Micro-typographical refinements (RS)
(c:/texlive/2024/texmf-dist/tex/latex/graphics/keyval.sty
Package: keyval 2022/05/29 v1.15 key=value parser (DPC)
\KV@toks@=\toks19
) (c:/texlive/2024/texmf-dist/tex/latex/etoolbox/etoolbox.sty
Package: etoolbox 2020/10/05 v2.5k e-TeX tools for LaTeX (JAW)
\etb@tempcnta=\count268
)
\MT@toks=\toks20
\MT@tempbox=\box52
\MT@count=\count269
LaTeX Info: Redefining \noprotrusionifhmode on input line 1061.
LaTeX Info: Redefining \leftprotrusion on input line 1062.
\MT@prot@toks=\toks21
LaTeX Info: Redefining \rightprotrusion on input line 1081.
LaTeX Info: Redefining \textls on input line 1392.
```

```
\MT@outer@kern=\dimen142
LaTeX Info: Redefining \textmicrotypecontext on input line 2013.
\MT@listname@count=\count270
(c:/texlive/2024/texmf-dist/tex/latex/microtype/microtype-pdftex.def
File: microtype-pdftex.def 2024/03/29 v3.1b Definitions specific to
pdftex (RS)

LaTeX Info: Redefining \lsstyle on input line 902.
LaTeX Info: Redefining \lslig on input line 902.
\MT@outer@space=\skip51
)
Package microtype Info: Loading configuration file microtype.cfg.
(c:/texlive/2024/texmf-dist/tex/latex/microtype/microtype.cfg
File: microtype.cfg 2024/03/29 v3.1b microtype main configuration file
(RS)
)) (c:/texlive/2024/texmf-dist/tex/latex/euler/euler.sty
Package: euler 1995/03/05 v2.5
Package: `euler' v2.5 <1995/03/05> (FJ and FMi)
LaTeX Font Info:    Redeclaring symbol font `letters' on input line 35.
LaTeX Font Info:    Encoding `OML' has changed to `U' for symbol font
(Font)              `letters' in the math version `normal' on input line
35.
LaTeX Font Info:    Overwriting symbol font `letters' in version `normal'
(Font)                  OML/cmm/m/it --> U/eur/m/n on input line 35.
LaTeX Font Info:    Encoding `OML' has changed to `U' for symbol font
(Font)              `letters' in the math version `bold' on input line
35.
LaTeX Font Info:    Overwriting symbol font `letters' in version `bold'
(Font)                  OML/cmm/b/it --> U/eur/m/n on input line 35.
LaTeX Font Info:    Overwriting symbol font `letters' in version `bold'
(Font)                  U/eur/m/n --> U/eur/b/n on input line 36.
LaTeX Font Info:    Redeclaring math symbol \Gamma on input line 47.
LaTeX Font Info:    Redeclaring math symbol \Delta on input line 48.
LaTeX Font Info:    Redeclaring math symbol \Theta on input line 49.
LaTeX Font Info:    Redeclaring math symbol \Lambda on input line 50.
LaTeX Font Info:    Redeclaring math symbol \Xi on input line 51.
LaTeX Font Info:    Redeclaring math symbol \Pi on input line 52.
LaTeX Font Info:    Redeclaring math symbol \Sigma on input line 53.
LaTeX Font Info:    Redeclaring math symbol \Upsilon on input line 54.
LaTeX Font Info:    Redeclaring math symbol \Phi on input line 55.
LaTeX Font Info:    Redeclaring math symbol \Psi on input line 56.
LaTeX Font Info:    Redeclaring math symbol \Omega on input line 57.
\symEulerFraktur=\mathgroup4
LaTeX Font Info:    Overwriting symbol font `EulerFraktur' in version
`bold'
(Font)                  U/euf/m/n --> U/euf/b/n on input line 63.
LaTeX Info: Redefining \oldstylenums on input line 85.
\symEulerScript=\mathgroup5
LaTeX Font Info:    Overwriting symbol font `EulerScript' in version
`bold'
(Font)                  U/eus/m/n --> U/eus/b/n on input line 93.
LaTeX Font Info:    Redeclaring math symbol \aleph on input line 97.
LaTeX Font Info:    Redeclaring math symbol \Re on input line 98.
LaTeX Font Info:    Redeclaring math symbol \Im on input line 99.
```

```
LaTeX Font Info:    Redeclaring math delimiter \vert on input line 101.
LaTeX Font Info:    Redeclaring math delimiter \backslash on input line
103.
LaTeX Font Info:    Redeclaring math symbol \neg on input line 106.
LaTeX Font Info:    Redeclaring math symbol \wedge on input line 108.
LaTeX Font Info:    Redeclaring math symbol \vee on input line 110.
LaTeX Font Info:    Redeclaring math symbol \setminus on input line 112.
LaTeX Font Info:    Redeclaring math symbol \sim on input line 113.
LaTeX Font Info:    Redeclaring math symbol \mid on input line 114.
LaTeX Font Info:    Redeclaring math delimiter \arrowvert on input line
116.
LaTeX Font Info:    Redeclaring math symbol \mathsection on input line
117.
\symEulerExtension=\mathgroup6
LaTeX Font Info:    Redeclaring math symbol \coprod on input line 125.
LaTeX Font Info:    Redeclaring math symbol \prod on input line 125.
LaTeX Font Info:    Redeclaring math symbol \sum on input line 125.
LaTeX Font Info:    Redeclaring math symbol \intop on input line 130.
LaTeX Font Info:    Redeclaring math symbol \ointop on input line 131.
LaTeX Font Info:    Redeclaring math symbol \braceld on input line 132.
LaTeX Font Info:    Redeclaring math symbol \bracerd on input line 133.
LaTeX Font Info:    Redeclaring math symbol \bracelu on input line 134.
LaTeX Font Info:    Redeclaring math symbol \braceru on input line 135.
LaTeX Font Info:    Redeclaring math symbol \infty on input line 136.
LaTeX Font Info:    Redeclaring math symbol \nearrow on input line 153.
LaTeX Font Info:    Redeclaring math symbol \searrow on input line 154.
LaTeX Font Info:    Redeclaring math symbol \nwarrow on input line 155.
LaTeX Font Info:    Redeclaring math symbol \swarrow on input line 156.
LaTeX Font Info:    Redeclaring math symbol \Leftrightarrow on input line
157.
LaTeX Font Info:    Redeclaring math symbol \Leftarrow on input line 158.
LaTeX Font Info:    Redeclaring math symbol \Rightarrow on input line
159.
LaTeX Font Info:    Redeclaring math symbol \leftrightarrow on input line
160.
LaTeX Font Info:    Redeclaring math symbol \leftarrow on input line 161.
LaTeX Font Info:    Redeclaring math symbol \rightarrow on input line
163.
LaTeX Font Info:    Redeclaring math delimiter \uparrow on input line
166.
LaTeX Font Info:    Redeclaring math delimiter \downarrow on input line
168.
LaTeX Font Info:    Redeclaring math delimiter \updownarrow on input line
170.
LaTeX Font Info:    Redeclaring math delimiter \Uparrow on input line
172.
LaTeX Font Info:    Redeclaring math delimiter \Downarrow on input line
174.
LaTeX Font Info:    Redeclaring math delimiter \Updownarrow on input line
176.
LaTeX Font Info:    Redeclaring math symbol \leftharpoonup on input line
177.
LaTeX Font Info:    Redeclaring math symbol \leftharpoondown on input
line 178.
```

```
LaTeX Font Info:    Redeclaring math symbol \rightharpoonup on input line
179.
LaTeX Font Info:    Redeclaring math symbol \rightharpoondown on input
line 180
.
LaTeX Font Info:    Redeclaring math delimiter \lbrace on input line 182.
LaTeX Font Info:    Redeclaring math delimiter \rbrace on input line 184.
\symcmmigroup=\mathgroup7
LaTeX Font Info:    Overwriting symbol font `cmmigroup' in version `bold'
(Font)                  OML/cmm/m/it --> OML/cmm/b/it on input line 200.
LaTeX Font Info:    Redeclaring math accent \vec on input line 201.
LaTeX Font Info:    Redeclaring math symbol \triangleleft on input line
202.
LaTeX Font Info:    Redeclaring math symbol \triangleright on input line
203.
LaTeX Font Info:    Redeclaring math symbol \star on input line 204.
LaTeX Font Info:    Redeclaring math symbol \lhook on input line 205.
LaTeX Font Info:    Redeclaring math symbol \rhook on input line 206.
LaTeX Font Info:    Redeclaring math symbol \flat on input line 207.
LaTeX Font Info:    Redeclaring math symbol \natural on input line 208.
LaTeX Font Info:    Redeclaring math symbol \sharp on input line 209.
LaTeX Font Info:    Redeclaring math symbol \smile on input line 210.
LaTeX Font Info:    Redeclaring math symbol \frown on input line 211.
LaTeX Font Info:    Redeclaring math accent \grave on input line 245.
LaTeX Font Info:    Redeclaring math accent \acute on input line 246.
LaTeX Font Info:    Redeclaring math accent \tilde on input line 247.
LaTeX Font Info:    Redeclaring math accent \ddot on input line 248.
LaTeX Font Info:    Redeclaring math accent \check on input line 249.
LaTeX Font Info:    Redeclaring math accent \breve on input line 250.
LaTeX Font Info:    Redeclaring math accent \bar on input line 251.
LaTeX Font Info:    Redeclaring math accent \dot on input line 252.
LaTeX Font Info:    Redeclaring math accent \hat on input line 254.
) (c:/texlive/2024/texmf-dist/tex/latex/multirow/multirow.sty
Package: multirow 2021/03/15 v2.8 Span multiple rows of a table
\multirow@colwidth=\skip52
\multirow@cntb=\count271
\multirow@dima=\skip53
\bigstrutjot=\dimen143
) (c:/texlive/2024/texmf-dist/tex/latex/algorithms/algorithm.sty
Package: algorithm 2009/08/24 v0.1 Document Style `algorithm' - floating
enviro
nment
(c:/texlive/2024/texmf-dist/tex/latex/float/float.sty
Package: float 2001/11/08 v1.3d Float enhancements (AL)
\c@float@type=\count272
\float@exts=\toks22
\float@box=\box53
\@float@everytoks=\toks23
\@floatcapt=\box54
) (c:/texlive/2024/texmf-dist/tex/latex/base/ifthen.sty
Package: ifthen 2024/03/16 v1.1e Standard LaTeX ifthen package (DPC)
)
\@float@every@algorithm=\toks24
```

```
\c@algorithm=\count273
) (c:/texlive/2024/texmf-dist/tex/latex/algorithmicx/algorithmicx.sty
Package: algorithmicx 2005/04/27 v1.2 Algorithmicx
Document Style algorithmicx 1.2 - a greatly improved `algorithmic' style
\c@ALG@line=\count274
\c@ALG@rem=\count275
\c@ALG@nested=\count276
\ALG@tlm=\skip54
\ALG@thistlm=\skip55
\c@ALG@Lnr=\count277
\c@ALG@blocknr=\count278
\c@ALG@storecount=\count279
\c@ALG@tmpcounter=\count280
\ALG@tmplength=\skip56
) (c:/texlive/2024/texmf-dist/tex/latex/merriweather/merriweather.sty
Package: merriweather 2022/09/20 (Bob Tennent) Supports
Merriweather(Sans) font
s for all LaTeX engines.
(c:/texlive/2024/texmf-dist/tex/generic/iftex/ifxetex.sty
Package: ifxetex 2019/10/25 v0.7 ifxetex legacy package. Use iftex
instead.
) (c:/texlive/2024/texmf-dist/tex/generic/iftex/ifluatex.sty
Package: ifluatex 2019/10/25 v1.5 ifluatex legacy package. Use iftex
instead.
) (c:/texlive/2024/texmf-dist/tex/latex/base/textcomp.sty
Package: textcomp 2024/04/24 v2.1b Standard LaTeX package
) (c:/texlive/2024/texmf-dist/tex/latex/xkeyval/xkeyval.sty
Package: xkeyval 2022/06/16 v2.9 package option processing (HA)
(c:/texlive/2024/texmf-dist/tex/generic/xkeyval/xkeyval.tex
(c:/texlive/2024/te
xmf-dist/tex/generic/xkeyval/xkvutils.tex
\XKV@toks=\toks25
\XKV@tempa@toks=\toks26
)
\XKV@depth=\count281
File: xkeyval.tex 2014/12/03 v2.7a key=value parser (HA)
)) (c:/texlive/2024/texmf-dist/tex/latex/base/fontenc.sty
Package: fontenc 2021/04/29 v2.0v Standard LaTeX package
) (c:/texlive/2024/texmf-dist/tex/latex/fontaxes/fontaxes.sty
Package: fontaxes 2020/07/21 v1.0e Font selection axes
LaTeX Info: Redefining \upshape on input line 29.
LaTeX Info: Redefining \itshape on input line 31.
LaTeX Info: Redefining \slshape on input line 33.
LaTeX Info: Redefining \swshape on input line 35.
LaTeX Info: Redefining \scshape on input line 37.
LaTeX Info: Redefining \sscshape on input line 39.
LaTeX Info: Redefining \ulcshape on input line 41.
LaTeX Info: Redefining \textsw on input line 47.
LaTeX Info: Redefining \textssc on input line 48.
LaTeX Info: Redefining \textulc on input line 49.
)) (c:/texlive/2024/texmf-dist/tex/latex/mathastext/mathastext.sty
Package: mathastext 2024/07/27 v1.4b Use the text font in math mode (JFB)

Package mathastext Info: Starting the math mode configuration.
```

```
\mst@exists@muskip=\muskip17
\mst@forall@muskip=\muskip18
\mst@prime@muskip=\muskip19
\mst@do@nonletters=\toks27
\mst@undo@nonletters=\toks28
\mst@do@easynonletters=\toks29
\mst@undo@easynonletters=\toks30
\symmtoperatorfont=\mathgroup8
\symmtletterfont=\mathgroup9
(   mathastext:   ) ! and ?
(   mathastext:   ) punctuation: , . : ; and \colon
LaTeX Info: Redefining \relbar on input line 1201.
LaTeX Info: Redefining \rightarrowfill on input line 1202.
LaTeX Info: Redefining \leftarrowfill on input line 1205.
(   mathastext:   ) + and =
LaTeX Info: Redefining \Relbar on input line 1298.
(   mathastext:   ) adding = ; and + to \nfss@catcodes
(   mathastext:   ) parentheses ( ) [ ] and slash /
(   mathastext:   ) alldelims: < > \backslash \setminus | \vert \mid \{
\}
LaTeX Font Info:    Redeclaring math symbol \setminus on input line 1364.
LaTeX Info: Redefining \models on input line 1383.
(   mathastext:   ) \# \mathdollar \% \&
(   mathastext:   ) \imath and \jmath
LaTeX Font Info:    Overwriting math alphabet `\Mathnormalbold' in
version `nor
mal'
(Font)                  T1/Merriwthr-OsF/b/it --> T1/Merriwthr-OsF/b/it
on inpu
t line 2863.
LaTeX Font Info:    Overwriting math alphabet `\Mathnormalbold' in
version `bol
d'
(Font)                  T1/Merriwthr-OsF/b/it --> T1/Merriwthr-OsF/b/it
on inpu
t line 2863.
LaTeX Font Info:    Overwriting symbol font `mtletterfont' in version
`normal'
(Font)                  T1/Merriwthr-OsF/m/it --> T1/Merriwthr-OsF/m/it
on inpu
t line 2863.
LaTeX Font Info:    Overwriting symbol font `mtletterfont' in version
`bold'
(Font)                  T1/Merriwthr-OsF/m/it --> T1/Merriwthr-OsF/b/it
on inpu
t line 2863.
LaTeX Font Info:    Overwriting symbol font `mtoperatorfont' in version
`normal
'
(Font)                  T1/Merriwthr-OsF/m/n --> T1/Merriwthr-OsF/m/n on
input
line 2863.
LaTeX Font Info:    Overwriting symbol font `mtoperatorfont' in version
`bold'
```

```
(Font)                  T1/Merriwthr-OsF/m/n --> T1/Merriwthr-OsF/b/n on
input
line 2863.
LaTeX Font Info:    Overwriting math alphabet `\Mathbf' in version
`normal'
(Font)                  T1/Merriwthr-OsF/b/n --> T1/Merriwthr-OsF/b/n on
input
line 2863.
LaTeX Font Info:    Overwriting math alphabet `\Mathbf' in version `bold'
(Font)                  T1/Merriwthr-OsF/b/n --> T1/Merriwthr-OsF/b/n on
input
line 2863.
LaTeX Font Info:    Overwriting math alphabet `\Mathit' in version
`normal'
(Font)                  T1/Merriwthr-OsF/m/it --> T1/Merriwthr-OsF/m/it
on inpu
t line 2863.
LaTeX Font Info:    Overwriting math alphabet `\Mathit' in version `bold'
(Font)                  T1/Merriwthr-OsF/m/it --> T1/Merriwthr-OsF/b/it
on inpu
t line 2863.
LaTeX Font Info:    Overwriting math alphabet `\Mathsf' in version
`normal'
(Font)                  T1/MerriwthrSans-OsF/m/n --> T1/MerriwthrSans-
OsF/m/n o
n input line 2863.
LaTeX Font Info:    Overwriting math alphabet `\Mathsf' in version `bold'
(Font)                  T1/MerriwthrSans-OsF/m/n --> T1/MerriwthrSans-
OsF/b/n o
n input line 2863.
LaTeX Font Info:    Overwriting math alphabet `\Mathtt' in version
`normal'
(Font)                  T1/lmtt/m/n --> T1/lmtt/m/n on input line 2863.
LaTeX Font Info:    Overwriting math alphabet `\Mathtt' in version `bold'
(Font)                  T1/lmtt/m/n --> T1/lmtt/b/n on input line 2863.
(   mathastext:   ) Latin letters in the `normal', resp. `bold',
(   mathastext:   ) math versions are now set up to use the fonts
(   mathastext:   ) T1/Merriwthr-OsF/m/it, resp. T1/Merriwthr-OsF/b/it.
(   mathastext:   ) Other characters (digits, ...) and \log-like names
will be
(   mathastext:   ) typeset with the n shape.
(   mathastext:   ) \hbar
(   mathastext:   ) minus as endash
(   mathastext:   ) The italic option is in effect.
(   mathastext:   ) \HUGE has been (re)-defined.
(   mathastext:   ) mathastext has declared larger sizes for subscripts.
(   mathastext:   ) To keep LaTeX defaults, use option
`defaultmathsizes'.

Package mathastext Info: Loading is complete.  You can now use
\Mathastext to
(mathastext)             modify the normal and bold math versions.  Use
it
```

```
(mathastext)             with optional argument or use \MTDeclareVersion
to
(mathastext)             declare additional math versions.
) (c:/texlive/2024/texmf-dist/tex/latex/relsize/relsize.sty
Package: relsize 2013/03/29 ver 4.1
) (c:/texlive/2024/texmf-dist/tex/latex/ragged2e/ragged2e.sty
Package: ragged2e 2023/06/22 v3.6 ragged2e Package
\CenteringLeftskip=\skip57
\RaggedLeftLeftskip=\skip58
\RaggedRightLeftskip=\skip59
\CenteringRightskip=\skip60
\RaggedLeftRightskip=\skip61
\RaggedRightRightskip=\skip62
\CenteringParfillskip=\skip63
\RaggedLeftParfillskip=\skip64
\RaggedRightParfillskip=\skip65
\JustifyingParfillskip=\skip66
\CenteringParindent=\skip67
\RaggedLeftParindent=\skip68
\RaggedRightParindent=\skip69
\JustifyingParindent=\skip70
) (c:/texlive/2024/texmf-dist/tex/latex/xcolor/xcolor.sty
Package: xcolor 2023/11/15 v3.01 LaTeX color extensions (UK)
(c:/texlive/2024/texmf-dist/tex/latex/graphics-cfg/color.cfg
File: color.cfg 2016/01/02 v1.6 sample color configuration
)
Package xcolor Info: Driver file: pdftex.def on input line 274.
(c:/texlive/2024/texmf-dist/tex/latex/graphics-def/pdftex.def
File: pdftex.def 2024/04/13 v1.2c Graphics/color driver for pdftex
) (c:/texlive/2024/texmf-dist/tex/latex/graphics/mathcolor.ltx)
Package xcolor Info: Model `cmy' substituted by `cmy0' on input line
1350.
Package xcolor Info: Model `hsb' substituted by `rgb' on input line 1354.
Package xcolor Info: Model `RGB' extended on input line 1366.
Package xcolor Info: Model `HTML' substituted by `rgb' on input line
1368.
Package xcolor Info: Model `Hsb' substituted by `hsb' on input line 1369.
Package xcolor Info: Model `tHsb' substituted by `hsb' on input line
1370.
Package xcolor Info: Model `HSB' substituted by `hsb' on input line 1371.
Package xcolor Info: Model `Gray' substituted by `gray' on input line
1372.
Package xcolor Info: Model `wave' substituted by `hsb' on input line
1373.
) (c:/texlive/2024/texmf-dist/tex/latex/colortbl/colortbl.sty
Package: colortbl 2024/07/06 v1.0i Color table columns (DPC)
(c:/texlive/2024/texmf-dist/tex/latex/tools/array.sty
Package: array 2024/06/14 v2.6d Tabular extension package (FMi)
\col@sep=\dimen144
\ar@mcellbox=\box55
\extrarowheight=\dimen145
\NC@list=\toks31
\extratabsurround=\skip71
\backup@length=\skip72
```

```
\ar@cellbox=\box56
)
\everycr=\toks32
\minrowclearance=\skip73
\rownum=\count282
) (c:/texlive/2024/texmf-dist/tex/latex/graphics/graphicx.sty
Package: graphicx 2021/09/16 v1.2d Enhanced LaTeX Graphics (DPC,SPQR)
(c:/texlive/2024/texmf-dist/tex/latex/graphics/graphics.sty
Package: graphics 2024/05/23 v1.4g Standard LaTeX Graphics (DPC,SPQR)
(c:/texlive/2024/texmf-dist/tex/latex/graphics/trig.sty
Package: trig 2023/12/02 v1.11 sin cos tan (DPC)
) (c:/texlive/2024/texmf-dist/tex/latex/graphics-cfg/graphics.cfg
File: graphics.cfg 2016/06/04 v1.11 sample graphics configuration
)
Package graphics Info: Driver file: pdftex.def on input line 106.
)
\Gin@req@height=\dimen146
\Gin@req@width=\dimen147
) (c:/texlive/2024/texmf-dist/tex/latex/xpatch/xpatch.sty
(c:/texlive/2024/texm
f-dist/tex/latex/l3kernel/expl3.sty
Package: expl3 2024-05-27 L3 programming layer (loader)
(c:/texlive/2024/texmf-dist/tex/latex/l3backend/l3backend-pdftex.def
File: l3backend-pdftex.def 2024-05-08 L3 backend support: PDF output
(pdfTeX)
\l__color_backend_stack_int=\count283
\l__pdf_internal_box=\box57
))
Package: xpatch 2020/03/25 v0.3a Extending etoolbox patching commands
(c:/texlive/2024/texmf-dist/tex/latex/l3packages/xparse/xparse.sty
Package: xparse 2024-05-08 L3 Experimental document command parser
)) (c:/texlive/2024/texmf-dist/tex/latex/environ/environ.sty
Package: environ 2014/05/04 v0.3 A new way to define environments
(c:/texlive/2024/texmf-dist/tex/latex/trimspaces/trimspaces.sty
Package: trimspaces 2009/09/17 v1.1 Trim spaces around a token list
)
\@envbody=\toks33
) (c:/texlive/2024/texmf-dist/tex/latex/lastpage/lastpage.sty
Package: lastpage 2024/07/07 v2.1c lastpage: 2.09 or 2e? (HMM)
(c:/texlive/2024/texmf-dist/tex/latex/lastpage/lastpage2e.sty
Package: lastpage2e 2024/07/07 v2.1c Decide which 2e lastpage version to
use (H
MM)
(c:/texlive/2024/texmf-dist/tex/latex/lastpage/lastpagemodern.sty
Package: lastpagemodern 2024-07-07 v2.1c Refers to last page's name (HMM;
JPG)
\c@lastpagecount=\count284
)
)) (c:/texlive/2024/texmf-dist/tex/latex/graphics/rotating.sty
Package: rotating 2016/08/11 v2.16d rotated objects in LaTeX
\c@r@tfl@t=\count285
\rotFPtop=\skip74
\rotFPbot=\skip75
\rot@float@box=\box58
```

```
\rot@mess@toks=\toks34
) (c:/texlive/2024/texmf-dist/tex/latex/graphics/lscape.sty
Package: lscape 2020/05/28 v3.02 Landscape Pages (DPC)
) (c:/texlive/2024/texmf-dist/tex/latex/tools/afterpage.sty
Package: afterpage 2023/07/04 v1.08 After-Page Package (DPC)
\AP@output=\toks35
\AP@partial=\box59
\AP@footins=\box60
) (c:/texlive/2024/texmf-dist/tex/latex/textpos/textpos.sty
Package: textpos 2022/07/23 v1.10.1
Package textpos Info: choosing support for LaTeX3 on input line 60.
\TP@textbox=\box61
\TP@holdbox=\box62
\TPHorizModule=\dimen148
\TPVertModule=\dimen149
\TP@margin=\dimen150
\TP@absmargin=\dimen151
Grid set 16 x 16 = 37.34424pt x 52.81541pt
\TPboxrulesize=\dimen152
\TP@ox=\dimen153
\TP@oy=\dimen154
\TP@tbargs=\toks36
TextBlockOrigin set to 0pt x 0pt
) (c:/texlive/2024/texmf-dist/tex/latex/url/url.sty
\Urlmuskip=\muskip20
Package: url 2013/09/16  ver 3.4  Verb mode for urls, etc.
) (c:/texlive/2024/texmf-dist/tex/latex/newfloat/newfloat.sty
Package: newfloat 2023/10/01 v1.2 Defining new floating environments (AR)
Package newfloat Info: `rotating' package detected.
) (c:/texlive/2024/texmf-dist/tex/latex/mdframed/mdframed.sty
Package: mdframed 2013/07/01 1.9b: mdframed
(c:/texlive/2024/texmf-dist/tex/latex/kvoptions/kvoptions.sty
Package: kvoptions 2022-06-15 v3.15 Key value format for package options
(HO)
(c:/texlive/2024/texmf-dist/tex/generic/ltxcmds/ltxcmds.sty
Package: ltxcmds 2023-12-04 v1.26 LaTeX kernel commands for general use
(HO)
) (c:/texlive/2024/texmf-dist/tex/latex/kvsetkeys/kvsetkeys.sty
Package: kvsetkeys 2022-10-05 v1.19 Key value parser (HO)
)) (c:/texlive/2024/texmf-dist/tex/latex/zref/zref-abspage.sty
Package: zref-abspage 2023-09-14 v2.35 Module abspage for zref (HO)
(c:/texlive/2024/texmf-dist/tex/latex/zref/zref-base.sty
Package: zref-base 2023-09-14 v2.35 Module base for zref (HO)
(c:/texlive/2024/texmf-dist/tex/generic/infwarerr/infwarerr.sty
Package: infwarerr 2019/12/03 v1.5 Providing info/warning/error messages
(HO)
) (c:/texlive/2024/texmf-dist/tex/generic/kvdefinekeys/kvdefinekeys.sty
Package: kvdefinekeys 2019-12-19 v1.6 Define keys (HO)
) (c:/texlive/2024/texmf-dist/tex/generic/pdftexcmds/pdftexcmds.sty
Package: pdftexcmds 2020-06-27 v0.33 Utility functions of pdfTeX for
LuaTeX (HO
)
Package pdftexcmds Info: \pdf@primitive is available.
Package pdftexcmds Info: \pdf@ifprimitive is available.
```

```
Package pdftexcmds Info: \pdfdraftmode found.
) (c:/texlive/2024/texmf-dist/tex/generic/etexcmds/etexcmds.sty
Package: etexcmds 2019/12/15 v1.7 Avoid name clashes with e-TeX commands
(HO)
) (c:/texlive/2024/texmf-dist/tex/latex/auxhook/auxhook.sty
Package: auxhook 2019-12-17 v1.6 Hooks for auxiliary files (HO)
)
Package zref Info: New property list: main on input line 767.
Package zref Info: New property: default on input line 768.
Package zref Info: New property: page on input line 769.
)
\c@abspage=\count286
Package zref Info: New property: abspage on input line 67.
) (c:/texlive/2024/texmf-dist/tex/latex/needspace/needspace.sty
Package: needspace 2010/09/12 v1.3d reserve vertical space
)
\mdf@templength=\skip76
\c@mdf@globalstyle@cnt=\count287
\mdf@skipabove@length=\skip77
\mdf@skipbelow@length=\skip78
\mdf@leftmargin@length=\skip79
\mdf@rightmargin@length=\skip80
\mdf@innerleftmargin@length=\skip81
\mdf@innerrightmargin@length=\skip82
\mdf@innertopmargin@length=\skip83
\mdf@innerbottommargin@length=\skip84
\mdf@splittopskip@length=\skip85
\mdf@splitbottomskip@length=\skip86
\mdf@outermargin@length=\skip87
\mdf@innermargin@length=\skip88
\mdf@linewidth@length=\skip89
\mdf@innerlinewidth@length=\skip90
\mdf@middlelinewidth@length=\skip91
\mdf@outerlinewidth@length=\skip92
\mdf@roundcorner@length=\skip93
\mdf@footenotedistance@length=\skip94
\mdf@userdefinedwidth@length=\skip95
\mdf@needspace@length=\skip96
\mdf@frametitleaboveskip@length=\skip97
\mdf@frametitlebelowskip@length=\skip98
\mdf@frametitlerulewidth@length=\skip99
\mdf@frametitleleftmargin@length=\skip100
\mdf@frametitlerightmargin@length=\skip101
\mdf@shadowsize@length=\skip102
\mdf@extratopheight@length=\skip103
\mdf@subtitleabovelinewidth@length=\skip104
\mdf@subtitlebelowlinewidth@length=\skip105
\mdf@subtitleaboveskip@length=\skip106
\mdf@subtitlebelowskip@length=\skip107
\mdf@subtitleinneraboveskip@length=\skip108
\mdf@subtitleinnerbelowskip@length=\skip109
\mdf@subsubtitleabovelinewidth@length=\skip110
\mdf@subsubtitlebelowlinewidth@length=\skip111
\mdf@subsubtitleaboveskip@length=\skip112
```

```
\mdf@subsubtitlebelowskip@length=\skip113
\mdf@subsubtitleinneraboveskip@length=\skip114
\mdf@subsubtitleinnerbelowskip@length=\skip115
(c:/texlive/2024/texmf-dist/tex/latex/mdframed/md-frame-0.mdf
File: md-frame-0.mdf 2013/07/01\ 1.9b: md-frame-0
)
\mdf@frametitlebox=\box63
\mdf@footnotebox=\box64
\mdf@splitbox@one=\box65
\mdf@splitbox@two=\box66
\mdf@splitbox@save=\box67
\mdfsplitboxwidth=\skip116
\mdfsplitboxtotalwidth=\skip117
\mdfsplitboxheight=\skip118
\mdfsplitboxdepth=\skip119
\mdfsplitboxtotalheight=\skip120
\mdfframetitleboxwidth=\skip121
\mdfframetitleboxtotalwidth=\skip122
\mdfframetitleboxheight=\skip123
\mdfframetitleboxdepth=\skip124
\mdfframetitleboxtotalheight=\skip125
\mdffootnoteboxwidth=\skip126
\mdffootnoteboxtotalwidth=\skip127
\mdffootnoteboxheight=\skip128
\mdffootnoteboxdepth=\skip129
\mdffootnoteboxtotalheight=\skip130
\mdftotallinewidth=\skip131
\mdfboundingboxwidth=\skip132
\mdfboundingboxtotalwidth=\skip133
\mdfboundingboxheight=\skip134
\mdfboundingboxdepth=\skip135
\mdfboundingboxtotalheight=\skip136
\mdf@freevspace@length=\skip137
\mdf@horizontalwidthofbox@length=\skip138
\mdf@verticalmarginwhole@length=\skip139
\mdf@horizontalspaceofbox=\skip140
\mdfsubtitleheight=\skip141
\mdfsubsubtitleheight=\skip142
\c@mdfcountframes=\count288

****** mdframed patching \endmdf@trivlist

****** -- success******

\mdf@envdepth=\count289
\c@mdf@env@i=\count290
\c@mdf@env@ii=\count291
\c@mdf@zref@counter=\count292
Package zref Info: New property: mdf@pagevalue on input line 895.
) (c:/texlive/2024/texmf-dist/tex/latex/titlesec/titlesec.sty
Package: titlesec 2023/10/27 v2.16 Sectioning titles
\ttl@box=\box68
\beforetitleunit=\skip143
\aftertitleunit=\skip144
```

```
\ttl@plus=\dimen155
\ttl@minus=\dimen156
\ttl@toksa=\toks37
\titlewidth=\dimen157
\titlewidthlast=\dimen158
\titlewidthfirst=\dimen159
) (c:/texlive/2024/texmf-dist/tex/latex/koma-script/scrextend.sty
Package: scrextend 2023/07/07 v3.41 KOMA-Script package (extend other
classes w
ith features of KOMA-Script classes)
(c:/texlive/2024/texmf-dist/tex/latex/koma-script/scrkbase.sty
Package: scrkbase 2023/07/07 v3.41 KOMA-Script package (KOMA-Script-
dependent b
asics and keyval usage)
(c:/texlive/2024/texmf-dist/tex/latex/koma-script/scrbase.sty
Package: scrbase 2023/07/07 v3.41 KOMA-Script package (KOMA-Script-
independent
basics and keyval usage)
(c:/texlive/2024/texmf-dist/tex/latex/koma-script/scrlfile.sty
Package: scrlfile 2023/07/07 v3.41 KOMA-Script package (file load hooks)
(c:/texlive/2024/texmf-dist/tex/latex/koma-script/scrlfile-hook.sty
Package: scrlfile-hook 2023/07/07 v3.41 KOMA-Script package (using LaTeX
hooks)

(c:/texlive/2024/texmf-dist/tex/latex/koma-script/scrlogo.sty
Package: scrlogo 2023/07/07 v3.41 KOMA-Script package (logo)
)))
Applying: [2021/05/01] Usage of raw or classic option list on input line
252.
Already applied: [0000/00/00] Usage of raw or classic option list on
input line
 368.
))
Package scrextend Info: unexpected definition of `\@makefnmark'.
(scrextend)             Trying to patch it on input line 1762.
Package scrextend Info: patch seems to be successfull on input line 1762.
)

LaTeX Font Warning: Font shape `T1/cmr/m/n' in size <7.5> not available
(Font)              size <7> substituted on input line 72.

(c:/texlive/2024/texmf-dist/tex/latex/tools/calc.sty
Package: calc 2023/07/08 v4.3 Infix arithmetic (KKT,FJ)
\calc@Acount=\count293
\calc@Bcount=\count294
\calc@Adimen=\dimen160
\calc@Bdimen=\dimen161
\calc@Askip=\skip145
\calc@Bskip=\skip146
LaTeX Info: Redefining \setlength on input line 80.
LaTeX Info: Redefining \addtolength on input line 81.
\calc@Ccount=\count295
\calc@Cskip=\skip147
) (c:/texlive/2024/texmf-dist/tex/latex/geometry/geometry.sty
```

```
Package: geometry 2020/01/02 v5.9 Page Geometry
(c:/texlive/2024/texmf-dist/tex/generic/iftex/ifvtex.sty
Package: ifvtex 2019/10/25 v1.7 ifvtex legacy package. Use iftex instead.
)
\Gm@cnth=\count296
\Gm@cntv=\count297
\c@Gm@tempcnt=\count298
\Gm@bindingoffset=\dimen162
\Gm@wd@mp=\dimen163
\Gm@odd@mp=\dimen164
\Gm@even@mp=\dimen165
\Gm@layoutwidth=\dimen166
\Gm@layoutheight=\dimen167
\Gm@layouthoffset=\dimen168
\Gm@layoutvoffset=\dimen169
\Gm@dimlist=\toks38
) (c:/texlive/2024/texmf-dist/tex/latex/preprint/authblk.sty
Package: authblk 2001/02/27 1.3 (PWD)
\affilsep=\skip148
\@affilsep=\skip149
\c@Maxaffil=\count299
\c@authors=\count300
\c@affil=\count301
) (c:/texlive/2024/texmf-dist/tex/latex/footmisc/footmisc.sty
Package: footmisc 2023/07/05 v6.0f a miscellany of footnote facilities
\FN@temptoken=\toks39
\footnotemargin=\dimen170
\@outputbox@depth=\dimen171
Package footmisc Info: Declaring symbol style bringhurst on input line
696.
Package footmisc Info: Declaring symbol style chicago on input line 704.
Package footmisc Info: Declaring symbol style wiley on input line 713.
Package footmisc Info: Declaring symbol style lamport-robust on input
line 724.

Package footmisc Info: Declaring symbol style lamport* on input line 744.
Package footmisc Info: Declaring symbol style lamport*-robust on input
line 765
.
) (c:/texlive/2024/texmf-dist/tex/latex/fancyhdr/fancyhdr.sty
Package: fancyhdr 2024/07/23 v4.3.1 Extensive control of page headers and
foote
rs
\f@nch@headwidth=\skip150
\f@nch@O@elh=\skip151
\f@nch@O@erh=\skip152
\f@nch@O@olh=\skip153
\f@nch@O@orh=\skip154
\f@nch@O@elf=\skip155
\f@nch@O@erf=\skip156
\f@nch@O@olf=\skip157
\f@nch@O@orf=\skip158
) (c:/texlive/2024/texmf-dist/tex/generic/alphalph/alphalph.sty
Package: alphalph 2019/12/09 v2.6 Convert numbers to letters (HO)
```

```
(c:/texlive/2024/texmf-dist/tex/generic/intcalc/intcalc.sty
Package: intcalc 2019/12/15 v1.3 Expandable calculations with integers
(HO)
))
\c@authorfn=\count302
(c:/texlive/2024/texmf-dist/tex/latex/abstract/abstract.sty
Package: abstract 2009/06/08 v1.2a configurable abstracts
\abstitleskip=\skip159
\absleftindent=\skip160
\absrightindent=\skip161
\absparindent=\skip162
\absparsep=\skip163
)
Package newfloat Info: New float `keypoints' with options
`placement=t!,name=kp
t' on input line 294.
\c@keypoints=\count303
Package newfloat Info: float type `keypoints'=8 on input line 294.
(c:/texlive/2024/texmf-dist/tex/latex/enumitem/enumitem.sty
Package: enumitem 2019/06/20 v3.9 Customized lists
\labelindent=\skip164
\enit@outerparindent=\dimen172
\enit@toks=\toks40
\enit@inbox=\box69
\enit@count@id=\count304
\enitdp@description=\count305
) (c:/texlive/2024/texmf-dist/tex/latex/quoting/quoting.sty
Package: quoting 2014/01/28 v0.1c Consolidated environment for displayed
text
\quo@toppartop=\skip165
) (c:/texlive/2024/texmf-dist/tex/latex/sttools/stfloats.sty
Package: stfloats 2017/03/27 v3.3 Improve float mechanism and
baselineskip sett
ings
\@dblbotnum=\count306
\c@dblbotnumber=\count307
) (c:/texlive/2024/texmf-dist/tex/latex/booktabs/booktabs.sty
Package: booktabs 2020/01/12 v1.61803398 Publication quality tables
\heavyrulewidth=\dimen173
\lightrulewidth=\dimen174
\cmidrulewidth=\dimen175
\belowrulesep=\dimen176
\belowbottomsep=\dimen177
\aboverulesep=\dimen178
\abovetopsep=\dimen179
\cmidrulesep=\dimen180
\cmidrulekern=\dimen181
\defaultaddspace=\dimen182
\@cmidla=\count308
\@cmidlb=\count309
\@aboverulesep=\dimen183
\@belowrulesep=\dimen184
\@thisruleclass=\count310
\@lastruleclass=\count311
```

```
\@thisrulewidth=\dimen185
) (c:/texlive/2024/texmf-dist/tex/latex/tools/tabularx.sty
Package: tabularx 2023/12/11 v2.12a `tabularx' package (DPC)
\TX@col@width=\dimen186
\TX@old@table=\dimen187
\TX@old@col=\dimen188
\TX@target=\dimen189
\TX@delta=\dimen190
\TX@cols=\count312
\TX@ftn=\toks41
)
\enitdp@tablenotes=\count313
(c:/texlive/2024/texmf-dist/tex/latex/caption/caption.sty
Package: caption 2023/08/05 v3.6o Customizing captions (AR)
(c:/texlive/2024/texmf-dist/tex/latex/caption/caption3.sty
Package: caption3 2023/07/31 v2.4d caption3 kernel (AR)
\caption@tempdima=\dimen191
\captionmargin=\dimen192
\caption@leftmargin=\dimen193
\caption@rightmargin=\dimen194
\caption@width=\dimen195
\caption@indent=\dimen196
\caption@parindent=\dimen197
\caption@hangindent=\dimen198
Package caption Info: Standard document class detected.
)
\c@caption@flags=\count314
\c@continuedfloat=\count315
Package caption Info: float package is loaded.
Package caption Info: rotating package is loaded.
Package caption Info: scrextend package is loaded.
\caption@addmargin@hsize=\dimen199
\caption@addmargin@linewidth=\dimen256
) (c:/texlive/2024/texmf-dist/tex/latex/natbib/natbib.sty
Package: natbib 2010/09/13 8.31b (PWD, AO)
\bibhang=\skip166
\bibsep=\skip167
LaTeX Info: Redefining \cite on input line 694.
\c@NAT@ctr=\count316
)) (c:/texlive/2024/texmf-dist/tex/latex/pgf/frontendlayer/tikz.sty
(c:/texlive
/2024/texmf-dist/tex/latex/pgf/basiclayer/pgf.sty (c:/texlive/2024/texmf-
dist/t
ex/latex/pgf/utilities/pgfrcs.sty (c:/texlive/2024/texmf-
dist/tex/generic/pgf/u
tilities/pgfutil-common.tex
\pgfutil@everybye=\toks42
\pgfutil@tempdima=\dimen257
\pgfutil@tempdimb=\dimen258
) (c:/texlive/2024/texmf-dist/tex/generic/pgf/utilities/pgfutil-latex.def
\pgfutil@abb=\box70
) (c:/texlive/2024/texmf-dist/tex/generic/pgf/utilities/pgfrcs.code.tex
(c:/tex
live/2024/texmf-dist/tex/generic/pgf/pgf.revision.tex)
```

```
Package: pgfrcs 2023-01-15 v3.1.10 (3.1.10)
))
Package: pgf 2023-01-15 v3.1.10 (3.1.10)
(c:/texlive/2024/texmf-dist/tex/latex/pgf/basiclayer/pgfcore.sty
(c:/texlive/20
24/texmf-dist/tex/latex/pgf/systemlayer/pgfsys.sty
(c:/texlive/2024/texmf-dist/
tex/generic/pgf/systemlayer/pgfsys.code.tex
Package: pgfsys 2023-01-15 v3.1.10 (3.1.10)
(c:/texlive/2024/texmf-dist/tex/generic/pgf/utilities/pgfkeys.code.tex
\pgfkeys@pathtoks=\toks43
\pgfkeys@temptoks=\toks44

(c:/texlive/2024/texmf-
dist/tex/generic/pgf/utilities/pgfkeyslibraryfiltered.co
de.tex
\pgfkeys@tmptoks=\toks45
))
\pgf@x=\dimen259
\pgf@y=\dimen260
\pgf@xa=\dimen261
\pgf@ya=\dimen262
\pgf@xb=\dimen263
\pgf@yb=\dimen264
\pgf@xc=\dimen265
\pgf@yc=\dimen266
\pgf@xd=\dimen267
\pgf@yd=\dimen268
\w@pgf@writea=\write3
\r@pgf@reada=\read2
\c@pgf@counta=\count317
\c@pgf@countb=\count318
\c@pgf@countc=\count319
\c@pgf@countd=\count320
\t@pgf@toka=\toks46
\t@pgf@tokb=\toks47
\t@pgf@tokc=\toks48
\pgf@sys@id@count=\count321
(c:/texlive/2024/texmf-dist/tex/generic/pgf/systemlayer/pgf.cfg
File: pgf.cfg 2023-01-15 v3.1.10 (3.1.10)
)
Driver file for pgf: pgfsys-pdftex.def
(c:/texlive/2024/texmf-dist/tex/generic/pgf/systemlayer/pgfsys-pdftex.def
File: pgfsys-pdftex.def 2023-01-15 v3.1.10 (3.1.10)
(c:/texlive/2024/texmf-dist/tex/generic/pgf/systemlayer/pgfsys-common-
pdf.def
File: pgfsys-common-pdf.def 2023-01-15 v3.1.10 (3.1.10)
)))
(c:/texlive/2024/texmf-
dist/tex/generic/pgf/systemlayer/pgfsyssoftpath.code.tex
File: pgfsyssoftpath.code.tex 2023-01-15 v3.1.10 (3.1.10)
\pgfsyssoftpath@smallbuffer@items=\count322
\pgfsyssoftpath@bigbuffer@items=\count323
)
```

```
(c:/texlive/2024/texmf-
dist/tex/generic/pgf/systemlayer/pgfsysprotocol.code.tex
File: pgfsysprotocol.code.tex 2023-01-15 v3.1.10 (3.1.10)
)) (c:/texlive/2024/texmf-
dist/tex/generic/pgf/basiclayer/pgfcore.code.tex
Package: pgfcore 2023-01-15 v3.1.10 (3.1.10)
(c:/texlive/2024/texmf-dist/tex/generic/pgf/math/pgfmath.code.tex
(c:/texlive/2
024/texmf-dist/tex/generic/pgf/math/pgfmathutil.code.tex)
(c:/texlive/2024/texm
f-dist/tex/generic/pgf/math/pgfmathparser.code.tex
\pgfmath@dimen=\dimen269
\pgfmath@count=\count324
\pgfmath@box=\box71
\pgfmath@toks=\toks49
\pgfmath@stack@operand=\toks50
\pgfmath@stack@operation=\toks51
) (c:/texlive/2024/texmf-
dist/tex/generic/pgf/math/pgfmathfunctions.code.tex)
(c:/texlive/2024/texmf-
dist/tex/generic/pgf/math/pgfmathfunctions.basic.code.te
x)
(c:/texlive/2024/texmf-
dist/tex/generic/pgf/math/pgfmathfunctions.trigonometric
.code.tex)
(c:/texlive/2024/texmf-
dist/tex/generic/pgf/math/pgfmathfunctions.random.code.t
ex)
(c:/texlive/2024/texmf-
dist/tex/generic/pgf/math/pgfmathfunctions.comparison.co
de.tex)
(c:/texlive/2024/texmf-
dist/tex/generic/pgf/math/pgfmathfunctions.base.code.tex
)
(c:/texlive/2024/texmf-
dist/tex/generic/pgf/math/pgfmathfunctions.round.code.te
x)
(c:/texlive/2024/texmf-
dist/tex/generic/pgf/math/pgfmathfunctions.misc.code.tex
)
(c:/texlive/2024/texmf-
dist/tex/generic/pgf/math/pgfmathfunctions.integerarithm
etics.code.tex) (c:/texlive/2024/texmf-
dist/tex/generic/pgf/math/pgfmathcalc.co
de.tex) (c:/texlive/2024/texmf-
dist/tex/generic/pgf/math/pgfmathfloat.code.tex
\c@pgfmathroundto@lastzeros=\count325
)) (c:/texlive/2024/texmf-dist/tex/generic/pgf/math/pgfint.code.tex)
(c:/texliv
e/2024/texmf-dist/tex/generic/pgf/basiclayer/pgfcorepoints.code.tex
File: pgfcorepoints.code.tex 2023-01-15 v3.1.10 (3.1.10)
\pgf@picminx=\dimen270
\pgf@picmaxx=\dimen271
\pgf@picminy=\dimen272
```

```
\pgf@picmaxy=\dimen273
\pgf@pathminx=\dimen274
\pgf@pathmaxx=\dimen275
\pgf@pathminy=\dimen276
\pgf@pathmaxy=\dimen277
\pgf@xx=\dimen278
\pgf@xy=\dimen279
\pgf@yx=\dimen280
\pgf@yy=\dimen281
\pgf@zx=\dimen282
\pgf@zy=\dimen283
)
(c:/texlive/2024/texmf-
dist/tex/generic/pgf/basiclayer/pgfcorepathconstruct.cod
e.tex
File: pgfcorepathconstruct.code.tex 2023-01-15 v3.1.10 (3.1.10)
\pgf@path@lastx=\dimen284
\pgf@path@lasty=\dimen285
)
(c:/texlive/2024/texmf-
dist/tex/generic/pgf/basiclayer/pgfcorepathusage.code.te
x
File: pgfcorepathusage.code.tex 2023-01-15 v3.1.10 (3.1.10)
\pgf@shorten@end@additional=\dimen286
\pgf@shorten@start@additional=\dimen287
) (c:/texlive/2024/texmf-
dist/tex/generic/pgf/basiclayer/pgfcorescopes.code.tex
File: pgfcorescopes.code.tex 2023-01-15 v3.1.10 (3.1.10)
\pgfpic=\box72
\pgf@hbox=\box73
\pgf@layerbox@main=\box74
\pgf@picture@serial@count=\count326
)
(c:/texlive/2024/texmf-
dist/tex/generic/pgf/basiclayer/pgfcoregraphicstate.code
.tex
File: pgfcoregraphicstate.code.tex 2023-01-15 v3.1.10 (3.1.10)
\pgflinewidth=\dimen288
)
(c:/texlive/2024/texmf-
dist/tex/generic/pgf/basiclayer/pgfcoretransformations.c
ode.tex
File: pgfcoretransformations.code.tex 2023-01-15 v3.1.10 (3.1.10)
\pgf@pt@x=\dimen289
\pgf@pt@y=\dimen290
\pgf@pt@temp=\dimen291
) (c:/texlive/2024/texmf-
dist/tex/generic/pgf/basiclayer/pgfcorequick.code.tex
File: pgfcorequick.code.tex 2023-01-15 v3.1.10 (3.1.10)
) (c:/texlive/2024/texmf-
dist/tex/generic/pgf/basiclayer/pgfcoreobjects.code.te
x
File: pgfcoreobjects.code.tex 2023-01-15 v3.1.10 (3.1.10)
)
```

```
(c:/texlive/2024/texmf-
dist/tex/generic/pgf/basiclayer/pgfcorepathprocessing.co
de.tex
File: pgfcorepathprocessing.code.tex 2023-01-15 v3.1.10 (3.1.10)
) (c:/texlive/2024/texmf-
dist/tex/generic/pgf/basiclayer/pgfcorearrows.code.tex
File: pgfcorearrows.code.tex 2023-01-15 v3.1.10 (3.1.10)
\pgfarrowsep=\dimen292
) (c:/texlive/2024/texmf-
dist/tex/generic/pgf/basiclayer/pgfcoreshade.code.tex
File: pgfcoreshade.code.tex 2023-01-15 v3.1.10 (3.1.10)
\pgf@max=\dimen293
\pgf@sys@shading@range@num=\count327
\pgf@shadingcount=\count328
) (c:/texlive/2024/texmf-
dist/tex/generic/pgf/basiclayer/pgfcoreimage.code.tex
File: pgfcoreimage.code.tex 2023-01-15 v3.1.10 (3.1.10)
)
(c:/texlive/2024/texmf-
dist/tex/generic/pgf/basiclayer/pgfcoreexternal.code.tex
File: pgfcoreexternal.code.tex 2023-01-15 v3.1.10 (3.1.10)
\pgfexternal@startupbox=\box75
) (c:/texlive/2024/texmf-
dist/tex/generic/pgf/basiclayer/pgfcorelayers.code.tex
File: pgfcorelayers.code.tex 2023-01-15 v3.1.10 (3.1.10)
)
(c:/texlive/2024/texmf-
dist/tex/generic/pgf/basiclayer/pgfcoretransparency.code
.tex
File: pgfcoretransparency.code.tex 2023-01-15 v3.1.10 (3.1.10)
)
(c:/texlive/2024/texmf-
dist/tex/generic/pgf/basiclayer/pgfcorepatterns.code.tex
File: pgfcorepatterns.code.tex 2023-01-15 v3.1.10 (3.1.10)
) (c:/texlive/2024/texmf-
dist/tex/generic/pgf/basiclayer/pgfcorerdf.code.tex
File: pgfcorerdf.code.tex 2023-01-15 v3.1.10 (3.1.10)
))) (c:/texlive/2024/texmf-
dist/tex/generic/pgf/modules/pgfmoduleshapes.code.te
x
File: pgfmoduleshapes.code.tex 2023-01-15 v3.1.10 (3.1.10)
\pgfnodeparttextbox=\box76
) (c:/texlive/2024/texmf-
dist/tex/generic/pgf/modules/pgfmoduleplot.code.tex
File: pgfmoduleplot.code.tex 2023-01-15 v3.1.10 (3.1.10)
)
(c:/texlive/2024/texmf-dist/tex/latex/pgf/compatibility/pgfcomp-version-
0-65.st
y
Package: pgfcomp-version-0-65 2023-01-15 v3.1.10 (3.1.10)
\pgf@nodesepstart=\dimen294
\pgf@nodesepend=\dimen295
)
```

```
(c:/texlive/2024/texmf-dist/tex/latex/pgf/compatibility/pgfcomp-version-
1-18.st
y
Package: pgfcomp-version-1-18 2023-01-15 v3.1.10 (3.1.10)
)) (c:/texlive/2024/texmf-dist/tex/latex/pgf/utilities/pgffor.sty
(c:/texlive/2
024/texmf-dist/tex/latex/pgf/utilities/pgfkeys.sty
(c:/texlive/2024/texmf-dist/
tex/generic/pgf/utilities/pgfkeys.code.tex)) (c:/texlive/2024/texmf-
dist/tex/la
tex/pgf/math/pgfmath.sty (c:/texlive/2024/texmf-
dist/tex/generic/pgf/math/pgfma
th.code.tex)) (c:/texlive/2024/texmf-
dist/tex/generic/pgf/utilities/pgffor.code
.tex
Package: pgffor 2023-01-15 v3.1.10 (3.1.10)
\pgffor@iter=\dimen296
\pgffor@skip=\dimen297
\pgffor@stack=\toks52
\pgffor@toks=\toks53
)) (c:/texlive/2024/texmf-
dist/tex/generic/pgf/frontendlayer/tikz/tikz.code.tex
Package: tikz 2023-01-15 v3.1.10 (3.1.10)

(c:/texlive/2024/texmf-
dist/tex/generic/pgf/libraries/pgflibraryplothandlers.co
de.tex
File: pgflibraryplothandlers.code.tex 2023-01-15 v3.1.10 (3.1.10)
\pgf@plot@mark@count=\count329
\pgfplotmarksize=\dimen298
)
\tikz@lastx=\dimen299
\tikz@lasty=\dimen300
\tikz@lastxsaved=\dimen301
\tikz@lastysaved=\dimen302
\tikz@lastmovetox=\dimen303
\tikz@lastmovetoy=\dimen304
\tikzleveldistance=\dimen305
\tikzsiblingdistance=\dimen306
\tikz@figbox=\box77
\tikz@figbox@bg=\box78
\tikz@tempbox=\box79
\tikz@tempbox@bg=\box80
\tikztreelevel=\count330
\tikznumberofchildren=\count331
\tikznumberofcurrentchild=\count332
\tikz@fig@count=\count333
(c:/texlive/2024/texmf-
dist/tex/generic/pgf/modules/pgfmodulematrix.code.tex
File: pgfmodulematrix.code.tex 2023-01-15 v3.1.10 (3.1.10)
\pgfmatrixcurrentrow=\count334
\pgfmatrixcurrentcolumn=\count335
\pgf@matrix@numberofcolumns=\count336
)
```

```
\tikz@expandcount=\count337

(c:/texlive/2024/texmf-
dist/tex/generic/pgf/frontendlayer/tikz/libraries/tikzli
brarytopaths.code.tex
File: tikzlibrarytopaths.code.tex 2023-01-15 v3.1.10 (3.1.10)
))) (c:/texlive/2024/texmf-dist/tex/latex/subfig/subfig.sty
Package: subfig 2005/06/28 ver: 1.3 subfig package
\c@KVtest=\count338
\sf@farskip=\skip168
\sf@captopadj=\dimen307
\sf@capskip=\skip169
\sf@nearskip=\skip170
\c@subfigure=\count339
\c@subfigure@save=\count340
\c@lofdepth=\count341
\c@subtable=\count342
\c@subtable@save=\count343
\c@lotdepth=\count344
\sf@top=\skip171
\sf@bottom=\skip172
) (c:/texlive/2024/texmf-dist/tex/latex/amsmath/amsmath.sty
Package: amsmath 2024/05/23 v2.17q AMS math features
\@mathmargin=\skip173
For additional information on amsmath, use the `?' option.
(c:/texlive/2024/texmf-dist/tex/latex/amsmath/amstext.sty
Package: amstext 2021/08/26 v2.01 AMS text
(c:/texlive/2024/texmf-dist/tex/latex/amsmath/amsgen.sty
File: amsgen.sty 1999/11/30 v2.0 generic functions
\@emptytoks=\toks54
\ex@=\dimen308
)) (c:/texlive/2024/texmf-dist/tex/latex/amsmath/amsbsy.sty
Package: amsbsy 1999/11/29 v1.2d Bold Symbols
\pmbraise@=\dimen309
) (c:/texlive/2024/texmf-dist/tex/latex/amsmath/amsopn.sty
Package: amsopn 2022/04/08 v2.04 operator names
)
\inf@bad=\count345
LaTeX Info: Redefining \frac on input line 233.
\uproot@=\count346
\leftroot@=\count347
LaTeX Info: Redefining \overline on input line 398.
LaTeX Info: Redefining \colon on input line 409.
\classnum@=\count348
\DOTSCASE@=\count349
LaTeX Info: Redefining \ldots on input line 495.
LaTeX Info: Redefining \dots on input line 498.
LaTeX Info: Redefining \cdots on input line 619.
\Mathstrutbox@=\box81
\strutbox@=\box82
LaTeX Info: Redefining \big on input line 721.
LaTeX Info: Redefining \Big on input line 722.
LaTeX Info: Redefining \bigg on input line 723.
LaTeX Info: Redefining \Bigg on input line 724.
```

```
\big@size=\dimen310
LaTeX Font Info:    Redeclaring font encoding OML on input line 742.
LaTeX Font Info:    Redeclaring font encoding OMS on input line 743.
\macc@depth=\count350
LaTeX Info: Redefining \bmod on input line 904.
LaTeX Info: Redefining \pmod on input line 909.
LaTeX Info: Redefining \smash on input line 939.
LaTeX Info: Redefining \relbar on input line 969.
LaTeX Info: Redefining \Relbar on input line 970.
\c@MaxMatrixCols=\count351
\dotsspace@=\muskip21
\c@parentequation=\count352
\dspbrk@lvl=\count353
\tag@help=\toks55
\row@=\count354
\column@=\count355
\maxfields@=\count356
\andhelp@=\toks56
\eqnshift@=\dimen311
\alignsep@=\dimen312
\tagshift@=\dimen313
\tagwidth@=\dimen314
\totwidth@=\dimen315
\lineht@=\dimen316
\@envbody=\toks57
\multlinegap=\skip174
\multlinetaggap=\skip175
\mathdisplay@stack=\toks58
LaTeX Info: Redefining \[ on input line 2953.
LaTeX Info: Redefining \] on input line 2954.
) (c:/texlive/2024/texmf-dist/tex/latex/orcidlink/orcidlink.sty
Package: orcidlink 2024/06/26 v1.1.0 Support ORCID's three different ID
formats
.
(c:/texlive/2024/texmf-dist/tex/latex/hyperref/hyperref.sty
Package: hyperref 2024-07-10 v7.01j Hypertext links for LaTeX
(c:/texlive/2024/texmf-dist/tex/generic/pdfescape/pdfescape.sty
Package: pdfescape 2019/12/09 v1.15 Implements pdfTeX's escape features
(HO)
) (c:/texlive/2024/texmf-dist/tex/latex/hycolor/hycolor.sty
Package: hycolor 2020-01-27 v1.10 Color options for hyperref/bookmark
(HO)
) (c:/texlive/2024/texmf-dist/tex/latex/hyperref/nameref.sty
Package: nameref 2023-11-26 v2.56 Cross-referencing by name of section
(c:/texlive/2024/texmf-dist/tex/latex/refcount/refcount.sty
Package: refcount 2019/12/15 v3.6 Data extraction from label references
(HO)
) (c:/texlive/2024/texmf-
dist/tex/generic/gettitlestring/gettitlestring.sty
Package: gettitlestring 2019/12/15 v1.6 Cleanup title references (HO)
)
\c@section@level=\count357
) (c:/texlive/2024/texmf-dist/tex/generic/stringenc/stringenc.sty
```

```
Package: stringenc 2019/11/29 v1.12 Convert strings between diff.
encodings (HO
)
)
\@linkdim=\dimen317
\Hy@linkcounter=\count358
\Hy@pagecounter=\count359
(c:/texlive/2024/texmf-dist/tex/latex/hyperref/pd1enc.def
File: pd1enc.def 2024-07-10 v7.01j Hyperref: PDFDocEncoding definition
(HO)
Now handling font encoding PD1 ...
... no UTF-8 mapping file for font encoding PD1
)
\Hy@SavedSpaceFactor=\count360
(c:/texlive/2024/texmf-dist/tex/latex/hyperref/puenc.def
File: puenc.def 2024-07-10 v7.01j Hyperref: PDF Unicode definition (HO)
Now handling font encoding PU ...
... no UTF-8 mapping file for font encoding PU
)
Package hyperref Info: Hyper figures OFF on input line 4157.
Package hyperref Info: Link nesting OFF on input line 4162.
Package hyperref Info: Hyper index ON on input line 4165.
Package hyperref Info: Plain pages OFF on input line 4172.
Package hyperref Info: Backreferencing OFF on input line 4177.
Package hyperref Info: Implicit mode ON; LaTeX internals redefined.
Package hyperref Info: Bookmarks ON on input line 4424.
\c@Hy@tempcnt=\count361
LaTeX Info: Redefining \url on input line 4763.
\XeTeXLinkMargin=\dimen318
(c:/texlive/2024/texmf-dist/tex/generic/bitset/bitset.sty
Package: bitset 2019/12/09 v1.3 Handle bit-vector datatype (HO)
(c:/texlive/2024/texmf-dist/tex/generic/bigintcalc/bigintcalc.sty
Package: bigintcalc 2019/12/15 v1.5 Expandable calculations on big
integers (HO
)
))
\Fld@menulength=\count362
\Field@Width=\dimen319
\Fld@charsize=\dimen320
Package hyperref Info: Hyper figures OFF on input line 6042.
Package hyperref Info: Link nesting OFF on input line 6047.
Package hyperref Info: Hyper index ON on input line 6050.
Package hyperref Info: backreferencing OFF on input line 6057.
Package hyperref Info: Link coloring OFF on input line 6062.
Package hyperref Info: Link coloring with OCG OFF on input line 6067.
Package hyperref Info: PDF/A mode OFF on input line 6072.
(c:/texlive/2024/texmf-dist/tex/latex/base/atbegshi-ltx.sty
Package: atbegshi-ltx 2021/01/10 v1.0c Emulation of the original atbegshi
package with kernel methods
)
\Hy@abspage=\count363
\c@Item=\count364
\c@Hfootnote=\count365
)
```

```
Package hyperref Info: Driver (autodetected): hpdftex.
(c:/texlive/2024/texmf-dist/tex/latex/hyperref/hpdftex.def
File: hpdftex.def 2024-07-10 v7.01j Hyperref driver for pdfTeX
(c:/texlive/2024/texmf-dist/tex/latex/base/atveryend-ltx.sty
Package: atveryend-ltx 2020/08/19 v1.0a Emulation of the original
atveryend pac
kage
with kernel methods
)
\HyAnn@Count=\count366
\Fld@listcount=\count367
\c@bookmark@seq@number=\count368
(c:/texlive/2024/texmf-dist/tex/latex/rerunfilecheck/rerunfilecheck.sty
Package: rerunfilecheck 2022-07-10 v1.10 Rerun checks for auxiliary files
(HO)
(c:/texlive/2024/texmf-dist/tex/generic/uniquecounter/uniquecounter.sty
Package: uniquecounter 2019/12/15 v1.4 Provide unlimited unique counter
(HO)
)
Package uniquecounter Info: New unique counter `rerunfilecheck' on input
line 2
85.
)
\Hy@SectionHShift=\skip176
)
(c:/texlive/2024/texmf-
dist/tex/generic/pgf/frontendlayer/tikz/libraries/tikzli
brarysvg.path.code.tex
File: tikzlibrarysvg.path.code.tex 2023-01-15 v3.1.10 (3.1.10)

(c:/texlive/2024/texmf-
dist/tex/generic/pgf/libraries/pgflibrarysvg.path.code.t
ex
File: pgflibrarysvg.path.code.tex 2023-01-15 v3.1.10 (3.1.10)
(c:/texlive/2024/texmf-
dist/tex/generic/pgf/modules/pgfmoduleparser.code.tex
File: pgfmoduleparser.code.tex 2023-01-15 v3.1.10 (3.1.10)
\pgfparserdef@arg@count=\count369
)
\pgf@lib@svg@last@x=\dimen321
\pgf@lib@svg@last@y=\dimen322
\pgf@lib@svg@last@c@x=\dimen323
\pgf@lib@svg@last@c@y=\dimen324
\pgf@lib@svg@count=\count370
\pgf@lib@svg@max@num=\count371
))
\@curXheight=\skip177
) (c:/texlive/2024/texmf-dist/tex/latex/svg/svg.sty
Package: svg 2020/11/26 v2.02k (include SVG pictures)
(c:/texlive/2024/texmf-dist/tex/latex/tools/shellesc.sty
Package: shellesc 2023/07/08 v1.0d unified shell escape interface for
LaTeX
Package shellesc Info: Restricted shell escape enabled on input line 77.
)
```

```
\c@svg@param@lastpage=\count372
\svg@box=\box83
\c@svg@param@currpage=\count373
) (c:/texlive/2024/texmf-dist/tex/latex/transparent/transparent.sty
Package: transparent 2022-10-27 v1.5 Transparency with color stacks
(c:/texlive/2024/texmf-dist/tex/latex/transparent/transparent-
nometadata.sty
Package: transparent-nometadata 2022-10-27 v1.5 Transparency via pdfTeX's
color
 stack (HO)
)) (c:/texlive/2024/texmf-dist/tex/generic/soul/soul.sty
Package: soul 2023-06-14 v3.1 Permit use of UTF-8 characters in soul (HO)
(c:/texlive/2024/texmf-dist/tex/generic/soul/soul-ori.sty
Package: soul-ori 2023-06-14 v3.1 letterspacing/underlining (mf)
\SOUL@word=\toks59
\SOUL@lasttoken=\toks60
\SOUL@syllable=\toks61
\SOUL@cmds=\toks62
\SOUL@buffer=\toks63
\SOUL@token=\toks64
\SOUL@syllgoal=\dimen325
\SOUL@syllwidth=\dimen326
\SOUL@charkern=\dimen327
\SOUL@hyphkern=\dimen328
\SOUL@dimen=\dimen329
\SOUL@dimeni=\dimen330
\SOUL@minus=\count374
\SOUL@comma=\count375
\SOUL@apo=\count376
\SOUL@grave=\count377
\SOUL@spaceskip=\skip178
\SOUL@ttwidth=\dimen331
\SOUL@uldp=\dimen332
\SOUL@ulht=\dimen333
)) (c:/texlive/2024/texmf-dist/tex/latex/siunitx/siunitx.sty
Package: siunitx 2024-06-24 v3.3.19 A comprehensive (SI) units package
\l__siunitx_number_uncert_offset_int=\count378
\l__siunitx_number_exponent_fixed_int=\count379
\l__siunitx_number_min_decimal_int=\count380
\l__siunitx_number_min_integer_int=\count381
\l__siunitx_number_round_precision_int=\count382
\l__siunitx_number_lower_threshold_int=\count383
\l__siunitx_number_upper_threshold_int=\count384
\l__siunitx_number_group_first_int=\count385
\l__siunitx_number_group_size_int=\count386
\l__siunitx_number_group_minimum_int=\count387
\l__siunitx_angle_tmp_dim=\dimen334
\l__siunitx_angle_marker_box=\box84
\l__siunitx_angle_unit_box=\box85
\l__siunitx_compound_count_int=\count388
(c:/texlive/2024/texmf-dist/tex/latex/translations/translations.sty
Package: translations 2022/02/05 v1.12 internationalization of LaTeX2e
packages
 (CN)
```

```
)
\l__siunitx_table_tmp_box=\box86
\l__siunitx_table_tmp_dim=\dimen335
\l__siunitx_table_column_width_dim=\dimen336
\l__siunitx_table_integer_box=\box87
\l__siunitx_table_decimal_box=\box88
\l__siunitx_table_uncert_box=\box89
\l__siunitx_table_before_box=\box90
\l__siunitx_table_after_box=\box91
\l__siunitx_table_before_dim=\dimen337
\l__siunitx_table_carry_dim=\dimen338
\l__siunitx_unit_tmp_int=\count389
\l__siunitx_unit_position_int=\count390
\l__siunitx_unit_total_int=\count391
) (c:/texlive/2024/texmf-dist/tex/latex/amsfonts/amssymb.sty
Package: amssymb 2013/01/14 v3.01 AMS font symbols
(c:/texlive/2024/texmf-dist/tex/latex/amsfonts/amsfonts.sty
Package: amsfonts 2013/01/14 v3.01 Basic AMSFonts support
\symAMSa=\mathgroup10
\symAMSb=\mathgroup11
LaTeX Font Info:    Redeclaring math symbol \hbar on input line 98.
LaTeX Info: Redefining \frak on input line 111.
)) (c:/texlive/2024/texmf-dist/tex/latex/lineno/lineno.sty
Package: lineno 2023/05/20 line numbers on paragraphs v5.3
\linenopenalty=\count392
\output=\toks65
\linenoprevgraf=\count393
\linenumbersep=\dimen339
\linenumberwidth=\dimen340
\c@linenumber=\count394
\c@pagewiselinenumber=\count395
\c@LN@truepage=\count396
\c@internallinenumber=\count397
\c@internallinenumbers=\count398
\quotelinenumbersep=\dimen341
\bframerule=\dimen342
\bframesep=\dimen343
\bframebox=\box92
\linenoamsmath@ams@eqpen=\count399
LaTeX Info: Redefining \\ on input line 3180.
)

! LaTeX Error: Option clash for package hyperref.

See the LaTeX manual or LaTeX Companion for explanation.
Type  H <return>  for immediate help.
 ...

l.76 \begin{document}

The package hyperref has already been loaded with options:
  []
There has now been an attempt to load it with options
  [colorlinks,allcolors=black,urlcolor=blue]
```

```
Adding the global options:
  ,colorlinks,allcolors=black,urlcolor=blue
to your \documentclass declaration may fix this.
Try typing  <return>  to proceed.

Package translations Info: No language package found. I am going to use
`englis
h' as default language. on input line 76.
LaTeX Font Info:    Trying to load font information for T1+Merriwthr-OsF
on inp
ut line 76.
(c:/texlive/2024/texmf-dist/tex/latex/merriweather/T1Merriwthr-OsF.fd
File: T1Merriwthr-OsF.fd 2020/08/30 (autoinst) Font definitions for
T1/Merriwth
r-OsF.
)
LaTeX Font Info:    Font shape `T1/Merriwthr-OsF/m/n' will be
(Font)              scaled to size 7.5pt on input line 76.
(./main.aux)
\openout1 = `main.aux'.

LaTeX Font Info:    Checking defaults for OML/cmm/m/it on input line 76.
LaTeX Font Info:    ... okay on input line 76.
LaTeX Font Info:    Checking defaults for OMS/cmsy/m/n on input line 76.
LaTeX Font Info:    ... okay on input line 76.
LaTeX Font Info:    Checking defaults for OT1/cmr/m/n on input line 76.
LaTeX Font Info:    ... okay on input line 76.
LaTeX Font Info:    Checking defaults for T1/cmr/m/n on input line 76.
LaTeX Font Info:    ... okay on input line 76.
LaTeX Font Info:    Checking defaults for TS1/cmr/m/n on input line 76.
LaTeX Font Info:    ... okay on input line 76.
LaTeX Font Info:    Checking defaults for OMX/cmex/m/n on input line 76.
LaTeX Font Info:    ... okay on input line 76.
LaTeX Font Info:    Checking defaults for U/cmr/m/n on input line 76.
LaTeX Font Info:    ... okay on input line 76.
LaTeX Font Info:    Checking defaults for PD1/pdf/m/n on input line 76.
LaTeX Font Info:    ... okay on input line 76.
LaTeX Font Info:    Checking defaults for PU/pdf/m/n on input line 76.
LaTeX Font Info:    ... okay on input line 76.
LaTeX Info: Redefining \microtypecontext on input line 76.
Package microtype Info: Applying patch `item' on input line 76.
Package microtype Info: Applying patch `toc' on input line 76.
Package microtype Info: Applying patch `eqnum' on input line 76.
Package microtype Info: Applying patch `footnote' on input line 76.
Package microtype Info: Applying patch `verbatim' on input line 76.
Package microtype Info: Generating PDF output.
Package microtype Info: Character protrusion enabled (level 2).
Package microtype Info: Using default protrusion set `alltext'.
Package microtype Info: Automatic font expansion enabled (level 2),
(microtype)             stretch: 20, shrink: 20, step: 1, non-selected.
Package microtype Info: Using default expansion set `alltext-nott'.
LaTeX Info: Redefining \showhyphens on input line 76.
Package microtype Info: No adjustment of tracking.
Package microtype Info: No adjustment of interword spacing.
```

```
Package microtype Info: No adjustment of character kerning.
Package microtype Info: Loading generic protrusion settings for font
family
(microtype)             `Merriwthr-OsF' (encoding: T1).
(microtype)             For optimal results, create family-specific
settings.
(microtype)             See the microtype manual for details.
LaTeX Font Info:    Redeclaring symbol font `operators' on input line 76.
LaTeX Font Info:    Encoding `OT1' has changed to `T1' for symbol font
(Font)              `operators' in the math version `normal' on input
line 76.
LaTeX Font Info:    Overwriting symbol font `operators' in version
`normal'
(Font)                  OT1/cmr/m/n --> T1/Merriwthr-OsF/m/up on input
line 76.

LaTeX Font Info:    Encoding `OT1' has changed to `T1' for symbol font
(Font)              `operators' in the math version `bold' on input line
76.
LaTeX Font Info:    Overwriting symbol font `operators' in version `bold'
(Font)                  OT1/cmr/bx/n --> T1/Merriwthr-OsF/m/up on input
line 76
.
LaTeX Font Info:    Overwriting symbol font `operators' in version `bold'
(Font)                  T1/Merriwthr-OsF/m/up --> T1/Merriwthr-OsF/b/up
on inpu
t line 76.
LaTeX Font Info:    Redeclaring math alphabet \mathbf on input line 76.
LaTeX Font Info:    Overwriting math alphabet `\mathbf' in version
`normal'
(Font)                  OT1/cmr/bx/n --> T1/Merriwthr-OsF/b/up on input
line 76
.
LaTeX Font Info:    Overwriting math alphabet `\mathbf' in version `bold'
(Font)                  OT1/cmr/bx/n --> T1/Merriwthr-OsF/b/up on input
line 76
.
LaTeX Font Info:    Redeclaring math alphabet \mathsf on input line 76.
LaTeX Font Info:    Overwriting math alphabet `\mathsf' in version
`normal'
(Font)                  OT1/cmss/m/n --> T1/MerriwthrSans-OsF/m/up on
input lin
e 76.
LaTeX Font Info:    Overwriting math alphabet `\mathsf' in version `bold'
(Font)                  OT1/cmss/bx/n --> T1/MerriwthrSans-OsF/m/up on
input li
ne 76.
LaTeX Font Info:    Redeclaring math alphabet \mathit on input line 76.
LaTeX Font Info:    Overwriting math alphabet `\mathit' in version
`normal'
(Font)                  OT1/cmr/m/it --> T1/Merriwthr-OsF/m/it on input
line 76
.
LaTeX Font Info:    Overwriting math alphabet `\mathit' in version `bold'
```

```
(Font)                  OT1/cmr/bx/it --> T1/Merriwthr-OsF/m/it on input
line 7
6.
LaTeX Font Info:    Redeclaring math alphabet \mathtt on input line 76.
LaTeX Font Info:    Overwriting math alphabet `\mathtt' in version
`normal'
(Font)                  OT1/cmtt/m/n --> T1/lmtt/m/up on input line 76.
LaTeX Font Info:    Overwriting math alphabet `\mathtt' in version `bold'
(Font)                  OT1/cmtt/m/n --> T1/lmtt/m/up on input line 76.
LaTeX Font Info:    Overwriting math alphabet `\mathsf' in version `bold'
(Font)                  T1/MerriwthrSans-OsF/m/up --> T1/MerriwthrSans-
OsF/b/up
 on input line 76.
LaTeX Font Info:    Overwriting math alphabet `\mathit' in version `bold'
(Font)                  T1/Merriwthr-OsF/m/it --> T1/Merriwthr-OsF/b/it
on inpu
t line 76.
\c@mv@tabular=\count400
\c@mv@boldtabular=\count401
(c:/texlive/2024/texmf-dist/tex/context/base/mkii/supp-pdf.mkii
[Loading MPS to PDF converter (version 2006.09.02).]
\scratchcounter=\count402
\scratchdimen=\dimen344
\scratchbox=\box93
\nofMPsegments=\count403
\nofMParguments=\count404
\everyMPshowfont=\toks66
\MPscratchCnt=\count405
\MPscratchDim=\dimen345
\MPnumerator=\count406
\makeMPintoPDFobject=\count407
\everyMPtoPDFconversion=\toks67
) (c:/texlive/2024/texmf-dist/tex/latex/epstopdf-pkg/epstopdf-base.sty
Package: epstopdf-base 2020-01-24 v2.11 Base part for package epstopdf
Package epstopdf-base Info: Redefining graphics rule for `.eps' on input
line 4
85.
(c:/texlive/2024/texmf-dist/tex/latex/latexconfig/epstopdf-sys.cfg
File: epstopdf-sys.cfg 2010/07/13 v1.3 Configuration of (r)epstopdf for
TeX Liv
e
))
Package newfloat Info: `float' package detected.
*geometry* driver: auto-detecting
*geometry* detected driver: pdftex
*geometry* verbose mode - [ preamble ] result:
* driver: pdftex
* paper: a4paper
* layout: <same size as paper>
* layoutoffset:(h,v)=(0.0pt,0.0pt)
* modes: includefoot twoside
* h-part:(L,W,R)=(54.64pt, 488.22787pt, 54.64pt)
* v-part:(T,H,B)=(66.0pt, 745.04684pt, 34.0pt)
* \paperwidth=597.50787pt
```

```
* \paperheight=845.04684pt
* \textwidth=488.22787pt
* \textheight=715.04684pt
* \oddsidemargin=-17.62999pt
* \evensidemargin=-17.62999pt
* \topmargin=-47.76999pt
* \headheight=17.5pt
* \headsep=24.0pt
* \topskip=10.0pt
* \footskip=30.0pt
* \marginparwidth=48.0pt
* \marginparsep=10.0pt
* \columnsep=18.0pt
* \skip\footins=22.0pt plus 2.0pt
* \hoffset=0.0pt
* \voffset=0.0pt
* \mag=1000
* \@twocolumntrue
* \@twosidetrue
* \@mparswitchtrue
* \@reversemarginfalse
* (1in=72.27pt=25.4mm, 1cm=28.453pt)

Package caption Info: Begin \AtBeginDocument code.
Package caption Info: subfig package v1.3 is loaded.
Package caption Info: hyperref package is loaded.
Package caption Info: End \AtBeginDocument code.
Package hyperref Info: Link coloring OFF on input line 76.
(./main.out) (./main.out)
\@outlinefile=\write4
\openout4 = `main.out'.


(c:/texlive/2024/texmf-dist/tex/latex/translations/translations-basic-
dictionar
y-english.trsl
File: translations-basic-dictionary-english.trsl (english translation
file `tra
nslations-basic-dictionary')
)
Package translations Info: loading dictionary `translations-basic-
dictionary' f
or `english'. on input line 76.
\@gscitedetails=\box94
\@gscitedetailsheight=\skip179
\@gsheadbox=\box95
\@gsheadboxheight=\skip180
LaTeX Font Info:    Font shape `T1/Merriwthr-OsF/b/n' will be
(Font)              scaled to size 6.5pt on input line 76.
LaTeX Font Info:    Calculating math sizes for size <7.5> on input line
76.

LaTeX Font Warning: Font shape `T1/Merriwthr-OsF/m/up' undefined
```

```
(Font)              using `T1/Merriwthr-OsF/m/n' instead on input line
76.

LaTeX Font Info:    Font shape `T1/Merriwthr-OsF/m/up' will be
(Font)              scaled to size 6.24973pt on input line 76.
LaTeX Font Info:    Font shape `T1/Merriwthr-OsF/m/up' will be
(Font)              scaled to size 5.24997pt on input line 76.
LaTeX Font Info:    Trying to load font information for U+eur on input
line 76.

(c:/texlive/2024/texmf-dist/tex/latex/amsfonts/ueur.fd
File: ueur.fd 2013/01/14 v3.01 Euler Roman
) (c:/texlive/2024/texmf-dist/tex/latex/microtype/mt-eur.cfg
File: mt-eur.cfg 2006/07/31 v1.1 microtype config. file: AMS Euler Roman
(RS)
)

LaTeX Font Warning: Font shape `OMS/cmsy/m/n' in size <7.5> not available
(Font)              size <7> substituted on input line 76.

LaTeX Font Info:    Trying to load font information for U+euf on input
line 76.

(c:/texlive/2024/texmf-dist/tex/latex/amsfonts/ueuf.fd
File: ueuf.fd 2013/01/14 v3.01 Euler Fraktur
) (c:/texlive/2024/texmf-dist/tex/latex/microtype/mt-euf.cfg
File: mt-euf.cfg 2006/07/03 v1.1 microtype config. file: AMS Euler
Fraktur (RS)

)
LaTeX Font Info:    Trying to load font information for U+eus on input
line 76.

(c:/texlive/2024/texmf-dist/tex/latex/amsfonts/ueus.fd
File: ueus.fd 2013/01/14 v3.01 Euler Script
) (c:/texlive/2024/texmf-dist/tex/latex/microtype/mt-eus.cfg
File: mt-eus.cfg 2006/07/28 v1.2 microtype config. file: AMS Euler Script
(RS)
)
LaTeX Font Info:    Trying to load font information for U+euex on input
line 76
.
(c:/texlive/2024/texmf-dist/tex/latex/amsfonts/ueuex.fd
File: ueuex.fd 2013/01/14 v3.01 Euler extra symbols
)

LaTeX Font Warning: Font shape `OML/cmm/m/it' in size <7.5> not available
(Font)              size <7> substituted on input line 76.

LaTeX Font Info:    Font shape `T1/Merriwthr-OsF/m/n' will be
(Font)              scaled to size 6.24973pt on input line 76.
LaTeX Font Info:    Font shape `T1/Merriwthr-OsF/m/n' will be
(Font)              scaled to size 5.24997pt on input line 76.
LaTeX Font Info:    Font shape `T1/Merriwthr-OsF/m/it' will be
```

```
(Font)              scaled to size 7.5pt on input line 76.
LaTeX Font Info:    Font shape `T1/Merriwthr-OsF/m/it' will be
(Font)              scaled to size 6.24973pt on input line 76.
LaTeX Font Info:    Font shape `T1/Merriwthr-OsF/m/it' will be
(Font)              scaled to size 5.24997pt on input line 76.
LaTeX Font Info:    Trying to load font information for U+msa on input
line 76.

(c:/texlive/2024/texmf-dist/tex/latex/amsfonts/umsa.fd
File: umsa.fd 2013/01/14 v3.01 AMS symbols A
) (c:/texlive/2024/texmf-dist/tex/latex/microtype/mt-msa.cfg
File: mt-msa.cfg 2006/02/04 v1.1 microtype config. file: AMS symbols (a)
(RS)
)
LaTeX Font Info:    Trying to load font information for U+msb on input
line 76.

(c:/texlive/2024/texmf-dist/tex/latex/amsfonts/umsb.fd
File: umsb.fd 2013/01/14 v3.01 AMS symbols B
) (c:/texlive/2024/texmf-dist/tex/latex/microtype/mt-msb.cfg
File: mt-msb.cfg 2005/06/01 v1.0 microtype config. file: AMS symbols (b)
(RS)
)
LaTeX Font Info:    Font shape `T1/Merriwthr-OsF/m/n' will be
(Font)              scaled to size 8.0pt on input line 76.
LaTeX Font Info:    Font shape `T1/Merriwthr-OsF/m/it' will be
(Font)              scaled to size 8.0pt on input line 76.
LaTeX Font Info:    Font shape `T1/Merriwthr-OsF/b/it' will be
(Font)              scaled to size 8.0pt on input line 76.
TextBlockOrigin set to 4pc+6.64pt x 4pc+6pt
<example-grid-100x100pt.png, id=128, 125.46875pt x 125.46875pt>
File: example-grid-100x100pt.png Graphic file (type png)
<use example-grid-100x100pt.png>
Package pdftex.def Info: example-grid-100x100pt.png  used on input line
92.
(pdftex.def)             Requested size: 42.00021pt x 42.0pt.

Overfull \hbox (54.64pt too wide) in paragraph at lines 92--92
[][]
 []

LaTeX Font Info:    Font shape `T1/Merriwthr-OsF/m/n' will be
(Font)              scaled to size 14.0pt on input line 92.
LaTeX Font Info:    Font shape `T1/Merriwthr-OsF/m/n' will be
(Font)              scaled to size 8.99997pt on input line 92.
LaTeX Font Info:    Calculating math sizes for size <14> on input line
92.
LaTeX Font Info:    Font shape `T1/Merriwthr-OsF/m/up' will be
(Font)              scaled to size 14.0pt on input line 92.
LaTeX Font Info:    Font shape `T1/Merriwthr-OsF/m/up' will be
(Font)              scaled to size 11.66617pt on input line 92.
LaTeX Font Info:    Font shape `T1/Merriwthr-OsF/m/up' will be
(Font)              scaled to size 9.79996pt on input line 92.
LaTeX Font Info:    Font shape `T1/Merriwthr-OsF/m/n' will be
```

```
(Font)              scaled to size 11.66617pt on input line 92.
LaTeX Font Info:    Font shape `T1/Merriwthr-OsF/m/n' will be
(Font)              scaled to size 9.79996pt on input line 92.
LaTeX Font Info:    Font shape `T1/Merriwthr-OsF/m/it' will be
(Font)              scaled to size 14.0pt on input line 92.
LaTeX Font Info:    Font shape `T1/Merriwthr-OsF/m/it' will be
(Font)              scaled to size 11.66617pt on input line 92.
LaTeX Font Info:    Font shape `T1/Merriwthr-OsF/m/it' will be
(Font)              scaled to size 9.79996pt on input line 92.
LaTeX Font Info:    Font shape `T1/Merriwthr-OsF/b/n' will be
(Font)              scaled to size 18.0pt on input line 92.
LaTeX Font Info:    Font shape `T1/Merriwthr-OsF/m/n' will be
(Font)              scaled to size 13.0pt on input line 92.
LaTeX Font Info:    Calculating math sizes for size <13> on input line
92.
LaTeX Font Info:    Font shape `T1/Merriwthr-OsF/m/up' will be
(Font)              scaled to size 13.0pt on input line 92.
LaTeX Font Info:    Font shape `T1/Merriwthr-OsF/m/up' will be
(Font)              scaled to size 10.83287pt on input line 92.
LaTeX Font Info:    Font shape `T1/Merriwthr-OsF/m/up' will be
(Font)              scaled to size 9.09996pt on input line 92.

LaTeX Font Warning: Font shape `OMS/cmsy/m/n' in size <13> not available
(Font)              size <12> substituted on input line 92.


LaTeX Font Warning: Font shape `OML/cmm/m/it' in size <13> not available
(Font)              size <12> substituted on input line 92.

LaTeX Font Info:    Font shape `T1/Merriwthr-OsF/m/n' will be
(Font)              scaled to size 10.83287pt on input line 92.
LaTeX Font Info:    Font shape `T1/Merriwthr-OsF/m/n' will be
(Font)              scaled to size 9.09996pt on input line 92.
LaTeX Font Info:    Font shape `T1/Merriwthr-OsF/m/it' will be
(Font)              scaled to size 13.0pt on input line 92.
LaTeX Font Info:    Font shape `T1/Merriwthr-OsF/m/it' will be
(Font)              scaled to size 10.83287pt on input line 92.
LaTeX Font Info:    Font shape `T1/Merriwthr-OsF/m/it' will be
(Font)              scaled to size 9.09996pt on input line 92.
LaTeX Font Info:    Trying to load font information for TS1+Merriwthr-OsF
on in
put line 92.
(c:/texlive/2024/texmf-dist/tex/latex/merriweather/TS1Merriwthr-OsF.fd
File: TS1Merriwthr-OsF.fd 2020/08/30 (autoinst) Font definitions for
TS1/Merriw
thr-OsF.
)
LaTeX Font Info:    Font shape `TS1/Merriwthr-OsF/m/n' will be
(Font)              scaled to size 10.83287pt on input line 92.
Package microtype Info: Loading generic protrusion settings for font
family
(microtype)             `Merriwthr-OsF' (encoding: TS1).
(microtype)             For optimal results, create family-specific
settings.
```

```
(microtype)             See the microtype manual for details.
LaTeX Font Info:    Font shape `T1/Merriwthr-OsF/m/n' will be
(Font)              scaled to size 9.0pt on input line 92.
LaTeX Font Info:    Font shape `T1/Merriwthr-OsF/m/up' will be
(Font)              scaled to size 9.0pt on input line 92.
LaTeX Font Info:    Font shape `T1/Merriwthr-OsF/m/up' will be
(Font)              scaled to size 7.0pt on input line 92.
LaTeX Font Info:    Font shape `T1/Merriwthr-OsF/m/up' will be
(Font)              scaled to size 5.0pt on input line 92.
LaTeX Font Info:    Font shape `T1/Merriwthr-OsF/m/n' will be
(Font)              scaled to size 7.0pt on input line 92.
LaTeX Font Info:    Font shape `T1/Merriwthr-OsF/m/n' will be
(Font)              scaled to size 5.0pt on input line 92.
LaTeX Font Info:    Font shape `T1/Merriwthr-OsF/m/it' will be
(Font)              scaled to size 9.0pt on input line 92.
LaTeX Font Info:    Font shape `T1/Merriwthr-OsF/m/it' will be
(Font)              scaled to size 7.0pt on input line 92.
LaTeX Font Info:    Font shape `T1/Merriwthr-OsF/m/it' will be
(Font)              scaled to size 5.0pt on input line 92.
LaTeX Font Info:    Font shape `T1/Merriwthr-OsF/m/n' will be
(Font)              scaled to size 6.5pt on input line 92.
LaTeX Font Info:    Calculating math sizes for size <6.5> on input line
92.
LaTeX Font Info:    Font shape `T1/Merriwthr-OsF/m/up' will be
(Font)              scaled to size 6.5pt on input line 92.
LaTeX Font Info:    Font shape `T1/Merriwthr-OsF/m/up' will be
(Font)              scaled to size 5.41643pt on input line 92.
LaTeX Font Info:    Font shape `T1/Merriwthr-OsF/m/up' will be
(Font)              scaled to size 4.54997pt on input line 92.

LaTeX Font Warning: Font shape `OMS/cmsy/m/n' in size <6.5> not available
(Font)              size <6> substituted on input line 92.


LaTeX Font Warning: Font shape `OMS/cmsy/m/n' in size <5.41643> not
available
(Font)              size <5> substituted on input line 92.


LaTeX Font Warning: Font shape `OMS/cmsy/m/n' in size <4.54997> not
available
(Font)              size <5> substituted on input line 92.


LaTeX Font Warning: Font shape `OML/cmm/m/it' in size <6.5> not available
(Font)              size <6> substituted on input line 92.


LaTeX Font Warning: Font shape `OML/cmm/m/it' in size <5.41643> not
available
(Font)              size <5> substituted on input line 92.
```

```
LaTeX Font Warning: Font shape `OML/cmm/m/it' in size <4.54997> not
available
(Font)              size <5> substituted on input line 92.

LaTeX Font Info:    Font shape `T1/Merriwthr-OsF/m/n' will be
(Font)              scaled to size 5.41643pt on input line 92.
LaTeX Font Info:    Font shape `T1/Merriwthr-OsF/m/n' will be
(Font)              scaled to size 4.54997pt on input line 92.
LaTeX Font Info:    Font shape `T1/Merriwthr-OsF/m/it' will be
(Font)              scaled to size 6.5pt on input line 92.
LaTeX Font Info:    Font shape `T1/Merriwthr-OsF/m/it' will be
(Font)              scaled to size 5.41643pt on input line 92.
LaTeX Font Info:    Font shape `T1/Merriwthr-OsF/m/it' will be
(Font)              scaled to size 4.54997pt on input line 92.
LaTeX Font Info:    Font shape `TS1/Merriwthr-OsF/m/n' will be
(Font)              scaled to size 5.41643pt on input line 92.

Overfull \hbox (54.64pt too wide) in paragraph at lines 92--92
[][][]
 []

LaTeX Font Info:    Font shape `T1/Merriwthr-OsF/b/n' will be
(Font)              scaled to size 10.0pt on input line 92.
LaTeX Font Info:    Font shape `T1/Merriwthr-OsF/b/n' will be
(Font)              scaled to size 8.0pt on input line 92.

Overfull \hbox (54.64pt too wide) in paragraph at lines 92--92
[][][]
 []


LaTeX Warning: Text page 1 contains only floats.


Overfull \vbox (13.43274pt too high) has occurred while \output is active
[]




LaTeX Warning: Text page 1 contains only floats.


Overfull \vbox (13.43274pt too high) has occurred while \output is active
[]



LaTeX Font Info:    Font shape `T1/Merriwthr-OsF/m/n' will be
(Font)              scaled to size 7.8pt on input line 92.
LaTeX Font Info:    Font shape `T1/Merriwthr-OsF/b/n' will be
(Font)              scaled to size 7.8pt on input line 92.
[1{c:/texlive/2024/texmf-
var/fonts/map/pdftex/updmap/pdftex.map}{c:/texlive/202
```

```
4/texmf-
dist/fonts/enc/dvips/merriweather/merriwthr_posqbl.enc}{c:/texlive/2024
/texmf-dist/fonts/enc/dvips/merriweather/merriwthr_owzwzj.enc}


 <c:/texlive/2024/texmf-dist/tex/latex/mwe/example-grid-100x100pt.png>]
Package mdframed Info: mdframed works in twoside mode on input line 95.
LaTeX Font Info:    Font shape `T1/Merriwthr-OsF/b/n' will be
(Font)              scaled to size 8.2pt on input line 95.
LaTeX Font Info:    Font shape `TS1/Merriwthr-OsF/m/n' will be
(Font)              scaled to size 7.5pt on input line 97.
Package mdframed Info: mdframed inside float
 mdframed uses option nobreak mdframed on input line 103.
Package mdframed Info: mdframed inside a box
 mdframed uses option nobreak mdframed on input line 103.
LaTeX Font Info:    Font shape `T1/Merriwthr-OsF/m/n' will be
(Font)              scaled to size 10.0pt on input line 105.
LaTeX Font Info:    Font shape `T1/Merriwthr-OsF/m/n' will be
(Font)              scaled to size 3.75pt on input line 105.
LaTeX Font Info:    Trying to load font information for T1+MerriwthrSans-
OsF on
 input line 105.
(c:/texlive/2024/texmf-dist/tex/latex/merriweather/T1MerriwthrSans-OsF.fd
File: T1MerriwthrSans-OsF.fd 2020/08/30 (autoinst) Font definitions for
T1/Merr
iwthrSans-OsF.
)
LaTeX Font Info:    Font shape `T1/MerriwthrSans-OsF/m/n' will be
(Font)              scaled to size 3.75pt on input line 105.
Package microtype Info: Loading generic protrusion settings for font
family
(microtype)             `MerriwthrSans-OsF' (encoding: T1).
(microtype)             For optimal results, create family-specific
settings.
(microtype)             See the microtype manual for details.
LaTeX Font Info:    Font shape `T1/Merriwthr-OsF/b/n' will be
(Font)              scaled to size 7.5pt on input line 106.

Package natbib Warning: Citation `omiye2024large' on page 2 undefined on
input
line 106.


Package natbib Warning: Citation `liu2021towards' on page 2 undefined on
input
line 106.


Package natbib Warning: Citation `comeau2013bioC' on page 2 undefined on
input
line 106.
```

Package natbib Warning: Citation `doan2014natural' on page 2 undefined on input
 line 106.

Package natbib Warning: Citation `cai2023integrating' on page 2 undefined on in
put line 106.

Package natbib Warning: Citation `chang2020benchmark' on page 2 undefined on in
put line 108.

Package natbib Warning: Citation `li2020real' on page 2 undefined on input line
 108.

Package natbib Warning: Citation `zheng2021pharmkg' on page 2 undefined on inpu
t line 108.

Package natbib Warning: Citation `wu2023medical' on page 2 undefined on input l
ine 108.

Package natbib Warning: Citation `zhu2020question' on page 2 undefined on input
 line 110.

Package natbib Warning: Citation `nentidis2023overview' on page 2 undefined on
input line 110.

Package natbib Warning: Citation `welbl2018constructing' on page 2 undefined on
 input line 110.

Package natbib Warning: Citation `abacha2019bridging' on page 2 undefined on in
put line 110.

Package natbib Warning: Citation `pal2022medmcqa' on page 2 undefined on input
line 110.

```
Package natbib Warning: Citation `Feng2024' on page 2 undefined on input line 1
10.


Package natbib Warning: Citation `lewis2020retrieval' on page 2 undefined on in
put line 112.


Package natbib Warning: Citation `jegal2023learning' on page 2 undefined on inp
ut line 112.


Package natbib Warning: Citation `izacardunsupervised' on page 2 undefined on i
nput line 112.


Package natbib Warning: Citation `asai2024self' on page 2 undefined on input li
ne 112.


Package natbib Warning: Citation `edge2024local' on page 2 undefined on input l
ine 112.


Package natbib Warning: Citation `yan2024corrective' on page 2 undefined on inp
ut line 112.


Package natbib Warning: Citation `sarthi2024raptor' on page 2 undefined on inpu
t line 112.


Package natbib Warning: Citation `tang2024medagents' on page 2 undefined on inp
ut line 112.


Underfull \hbox (badness 1360) in paragraph at lines 112--113
[]\T1/Merriwthr-OsF/m/n/7.5 (+20) Retrieval-Augmented Gen-er-a-tion (RAG) has e
merged as a
 []
```

```
Package natbib Warning: Citation `jaech2024openai' on page 2 undefined on
input
 line 114.


Package natbib Warning: Citation `guo2025deepseek' on page 2 undefined on
input
 line 114.



pdfTeX warning: pdflatex.exe (file ./application_3.pdf): PDF inclusion:
found P
DF version <1.7>, but at most version <1.5> allowed
<application_3.pdf, id=145, 586.10968pt x 218.7372pt>
File: application_3.pdf Graphic file (type pdf)
<use application_3.pdf>
Package pdftex.def Info: application_3.pdf  used on input line 118.
(pdftex.def)             Requested size: 488.22787pt x 182.20934pt.
LaTeX Font Info:    Font shape `T1/Merriwthr-OsF/m/n' will be
(Font)              scaled to size 6.0pt on input line 120.
LaTeX Font Info:    Font shape `T1/Merriwthr-OsF/b/n' will be
(Font)              scaled to size 6.0pt on input line 120.

Underfull \vbox (badness 3260) has occurred while \output is active []



LaTeX Font Info:    Font shape `T1/Merriwthr-OsF/m/it' will be
(Font)              scaled to size 7.8pt on input line 128.
[2{c:/texlive/2024/texmf-
dist/fonts/enc/dvips/merriweather/merriwthr_ags7qn.enc
}]
LaTeX Font Info:    Font shape `T1/Merriwthr-OsF/b/n' will be
(Font)              scaled to size 8.5pt on input line 130.

Underfull \vbox (badness 10000) has occurred while \output is active []



LaTeX Font Info:    Font shape `T1/Merriwthr-OsF/m/n' will be
(Font)              scaled to size 8.5pt on input line 130.

Package natbib Warning: Citation `openai_gpt-4o-mini_2024' on page 3
undefined
on input line 131.



pdfTeX warning: pdflatex.exe (file ./dataset_construction_2.pdf): PDF
inclusion
: found PDF version <1.7>, but at most version <1.5> allowed
<dataset_construction_2.pdf, id=157, 883.38025pt x 447.59218pt>
```

```
File: dataset_construction_2.pdf Graphic file (type pdf)
<use dataset_construction_2.pdf>
Package pdftex.def Info: dataset_construction_2.pdf  used on input line
138.
(pdftex.def)             Requested size: 488.22787pt x 247.37164pt.
LaTeX Font Info:    Font shape `T1/Merriwthr-OsF/m/n' will be
(Font)              scaled to size 6.25008pt on input line 138.
LaTeX Font Info:    Font shape `T1/Merriwthr-OsF/b/n' will be
(Font)              scaled to size 6.25008pt on input line 138.

Overfull \hbox (3.525pt too wide) in paragraph at lines 138--138
 [][]
 []

<rag_KG.pdf, id=158, 1300.86pt x 578.16pt>
File: rag_KG.pdf Graphic file (type pdf)
<use rag_KG.pdf>
Package pdftex.def Info: rag_KG.pdf  used on input line 143.
(pdftex.def)             Requested size: 239.23433pt x 106.32274pt.
<rag_dataset.pdf, id=159, 1092.72241pt x 656.93428pt>
File: rag_dataset.pdf Graphic file (type pdf)
<use rag_dataset.pdf>
Package pdftex.def Info: rag_dataset.pdf  used on input line 148.
(pdftex.def)             Requested size: 239.23433pt x 143.82431pt.
LaTeX Font Info:    Font shape `T1/Merriwthr-OsF/b/sl' in size <7.5> not
availa
ble
(Font)              Font shape `T1/Merriwthr-OsF/b/it' tried instead on
input l
ine 157.
LaTeX Font Info:    Font shape `T1/Merriwthr-OsF/b/it' will be
(Font)              scaled to size 7.5pt on input line 157.

Package natbib Warning: Citation `marker_repo' on page 3 undefined on
input lin
e 167.



[3 <./application_3.pdf>]
Underfull \hbox (badness 3396) in paragraph at lines 170--171
\T1/Merriwthr-OsF/m/n/7.5 (+20) We lever-age the GPT-4o mini to ex-tract
biomed
ical-related
 []


Underfull \hbox (badness 1152) in paragraph at lines 170--171
\T1/Merriwthr-OsF/m/n/7.5 (+20) from re-search pa-pers to con-struct an
entity-
level knowl-edge
 []
```

```
Underfull \hbox (badness 4254) in paragraph at lines 170--171
\T1/Merriwthr-OsF/m/n/7.5 (+20) graph. By iden-ti-fy-ing re-cur-ring
biomed-i-c
al terms, re-lated
 []


Underfull \hbox (badness 4543) in paragraph at lines 170--171
\T1/Merriwthr-OsF/m/n/7.5 (+20) can be ac-cessed at
[]https://github.com/yichun
10/BioCDQA.git[].
 []


Underfull \hbox (badness 1418) in paragraph at lines 170--171
\T1/Merriwthr-OsF/m/n/7.5 (+20) of the nodes and re-la-tion-ships are
pro-vided
 Fig-ure []2b[]. The
 []


Underfull \hbox (badness 10000) in paragraph at lines 170--171

 []


Underfull \vbox (badness 3407) has occurred while \output is active []



Underfull \vbox (badness 10000) has occurred while \output is active []


[4 <./dataset_construction_2.pdf> <./rag_KG.pdf> <./rag_dataset.pdf>]
LaTeX Font Info:    Font shape `T1/Merriwthr-OsF/m/up' will be
(Font)              scaled to size 7.5pt on input line 175.

Package natbib Warning: Citation `nlm_mesh_2023' on page 5 undefined on
input l
ine 182.


Package natbib Warning: Citation `uniprot2023uniprot' on page 5 undefined
on in
put line 182.


Package natbib Warning: Citation `wang2020minilm' on page 5 undefined on
input
line 182.
```

```
Underfull \hbox (badness 10000) in paragraph at lines 184--185
\T1/Merriwthr-OsF/b/n/7.5 (+20) Constructing Entity-Level Knowl-edge
Graphs\T1/
Merriwthr-OsF/m/up/7.5 (+20) . Once the
 []


Underfull \hbox (badness 1122) in paragraph at lines 184--185
\T1/Merriwthr-OsF/m/up/7.5 (+20) di-rected edge be-tween them. The pro-
cess be-
gins with node
 []


Underfull \hbox (badness 1596) in paragraph at lines 184--185
\T1/Merriwthr-OsF/m/up/7.5 (+20) re-la-tion-ship chains be-tween en-ti-
ties, fa
-cil-i-tat-ing flex-i-ble and
 []


Underfull \hbox (badness 10000) in paragraph at lines 184--185

 []




Underfull \hbox (badness 3746) in paragraph at lines 206--207
\T1/Merriwthr-OsF/m/up/7.5 (+20) Based on BioS-trataKG, we in-tro-duce a
biomed
-i-cal question-
 []


Underfull \vbox (badness 10000) has occurred while \output is active []



[5]
Underfull \hbox (badness 1199) in paragraph at lines 222--224
\T1/Merriwthr-OsF/m/up/7.5 (+20) find-ings and con-clu-sions pre-sented
in the
lit-er-a-ture but also
 []


Underfull \hbox (badness 1057) in paragraph at lines 222--224
\T1/Merriwthr-OsF/m/up/7.5 (+20) as-sist in de-sign-ing sub-se-quent re-
search
plans. By an-a-lyz-ing
 []
```

```
Underfull \hbox (badness 1057) in paragraph at lines 222--224
\T1/Merriwthr-OsF/m/up/7.5 (+20) Over-all, knowl-edge dis-cov-ery ques-
tions pl
ay a piv-otal role in
 []


Underfull \hbox (badness 10000) in paragraph at lines 222--224

 []


Underfull \hbox (badness 10000) in paragraph at lines 250--251

 []

[6]

! Package svg Error: File `ourrag_4.svg' is missing.

See the svg package documentation for explanation.
Type  H <return>  for immediate help.
 ...

l.268 \includesvg[width=1\linewidth]{ourrag_4.svg}

There's no file `ourrag_4.svg'
neither in the current directory nor any other searched
path given by \svgpath or \graphicspath.
No additional path was given.


Package natbib Warning: Citation `liu2024deepseek' on page 7 undefined on
input
 line 277.


Package natbib Warning: Citation `izacardunsupervised' on page 7
undefined on i
nput line 281.


Package natbib Warning: Citation `guo2025deepseek' on page 7 undefined on
input
 line 310.
```

```
[7]
LaTeX Font Info:    Font shape `T1/Merriwthr-OsF/b/n' will be
(Font)              scaled to size 7.0pt on input line 354.



Package natbib Warning: Citation `hurst2024gpt' on page 8 undefined on
input li
ne 487.


Underfull \hbox (badness 10000) in paragraph at lines 513--513
|\T1/Merriwthr-OsF/b/n/8.5 (+20) Analysis of the Re-call for Multi-Level
and Mu
lti-
 []



[8]
Underfull \vbox (badness 10000) has occurred while \output is active []




Underfull \hbox (badness 8113) in paragraph at lines 520--521
[]\T1/Merriwthr-OsF/m/up/7.5 (+20) In sum-mary, the in-te-gra-tion of
multi-lev
el and multi-
 []

! Undefined control sequence.
l.554 ...36.80 & 31.28 & 40.04 & 40.04 & \makecell
                                                  {Only Question-based}
\\
The control sequence at the end of the top line
of your error message was never \def'ed. If you have
misspelled it (e.g., `\hobx'), type `I' and the correct
spelling (e.g., `I\hbox'). Otherwise just continue,
and I'll forget about whatever was undefined.

! Undefined control sequence.
l.555 ...34.79 & 27.63 & 39.62 & 44.06 & \makecell
                                                  {+ Virtual Answer-
based} \\
The control sequence at the end of the top line
of your error message was never \def'ed. If you have
misspelled it (e.g., `\hobx'), type `I' and the correct
spelling (e.g., `I\hbox'). Otherwise just continue,
and I'll forget about whatever was undefined.

! Undefined control sequence.
l.556 ... 4.10 & 42.46 & 42.46 & 66.10 & \makecell
                                                  {+ Keyword-based} \\
```

```
The control sequence at the end of the top line
of your error message was never \def'ed. If you have
misspelled it (e.g., `\hobx'), type `I' and the correct
spelling (e.g., `I\hbox'). Otherwise just continue,
and I'll forget about whatever was undefined.



[9]

Package natbib Warning: Citation `rajpal2014mining' on page 10 undefined
on inp
ut line 610.


Package natbib Warning: Citation `mohs2017drug' on page 10 undefined on
input l
ine 610.




Package natbib Warning: Citation `maher2014clinical' on page 10 undefined
on in
put line 619.


Package natbib Warning: Citation `neuvonen2006drug' on page 10 undefined
on inp
ut line 619.



[10]

pdfTeX warning: pdflatex.exe (file ./Scientific Questions_5.pdf): PDF
inclusion
: found PDF version <1.7>, but at most version <1.5> allowed
<Scientific Questions_5.pdf, id=448, 1046.95131pt x 529.01637pt>
File: Scientific Questions_5.pdf Graphic file (type pdf)
<use Scientific Questions_5.pdf>
Package pdftex.def Info: Scientific Questions_5.pdf  used on input line
635.
(pdftex.def)             Requested size: 439.4021pt x 222.02391pt.


pdfTeX warning: pdflatex.exe (file ./Drug_Interaction_5.pdf): PDF
inclusion: fo
und PDF version <1.7>, but at most version <1.5> allowed
<Drug_Interaction_5.pdf, id=449, 1052.01025pt x 515.04419pt>
File: Drug_Interaction_5.pdf Graphic file (type pdf)
<use Drug_Interaction_5.pdf>
Package pdftex.def Info: Drug_Interaction_5.pdf  used on input line 642.
(pdftex.def)             Requested size: 439.4021pt x 215.12253pt.
```

```
Underfull \hbox (badness 1184) in paragraph at lines 649--650
\T1/Merriwthr-OsF/m/up/7.5 (+20) To ad-vance biomed-i-cal knowl-edge ex-
trac-ti
on and ap-pli-ca-tion,
 []




Underfull \hbox (badness 10000) in paragraph at lines 667--668
[]\T1/Merriwthr-OsF/m/up/7.5 (+20) Computational Work-flow DOI:
 []


Package natbib Warning: Citation `Feng_2025' on page 11 undefined on
input line
 672.


Underfull \hbox (badness 3364) in paragraph at lines 678--680
\T1/Merriwthr-OsF/m/up/7.5 (+20) Augmented Gen-er-a-tion; IP-RAR: In-te-
grated
and Pro-gres-sive
 []

No file main.bbl.

Package natbib Warning: There were undefined citations.



[11] [12 <./Scientific Questions_5.pdf> <./Drug_Interaction_5.pdf>]
enddocument/afterlastpage: lastpage setting LastPage.
(./main.aux)
 ***********
LaTeX2e <2024-06-01> patch level 2
L3 programming layer <2020/03/25>
 ***********

LaTeX Font Warning: Size substitutions with differences
(Font)              up to 1.0pt have occurred.


LaTeX Font Warning: Some font shapes were not available, defaults
substituted.

Package rerunfilecheck Info: File `main.out' has not changed.
(rerunfilecheck)             Checksum:
9B92BC7EEC1A8EEDB0DF25E7AC3E0D9E;6884.
 )
Here is how much of TeX's memory you used:
 37517 strings out of 473583
 752344 string characters out of 5732343
```

```
 2004908 words of memory out of 5000000
 59012 multiletter control sequences out of 15000+600000
 1928495 words of font info for 625 fonts, out of 8000000 for 9000
 1141 hyphenation exceptions out of 8191
 123i,12n,131p,1905b,1069s stack positions out of
10000i,1000n,20000p,200000b,200000s

pdfTeX warning (dest): name{https://github.com/yichun10/BioCDQA.git} has
been r
eferenced but does not exist, replaced by a fixed one

<c:/texlive/2024/texmf-dist/fonts/type1/sorkin/merriweather/Merriwthr-
Bold.pfb>
<c:/texlive/2024/texmf-dist/fonts/type1/sorkin/merriweather/Merriwthr-
BoldItali
c.pfb><c:/texlive/2024/texmf-
dist/fonts/type1/sorkin/merriweather/Merriwthr-Ita
lic.pfb><c:/texlive/2024/texmf-
dist/fonts/type1/sorkin/merriweather/Merriwthr-R
egular.pfb><c:/texlive/2024/texmf-
dist/fonts/type1/sorkin/merriweather/Merriwth
rSans-Regular.pfb><c:/texlive/2024/texmf-
dist/fonts/type1/public/amsfonts/cm/cm
sy7.pfb>
Output written on main.pdf (12 pages, 2350706 bytes).
PDF statistics:
 552 PDF objects out of 1000 (max. 8388607)
 321 compressed objects within 4 object streams
 63 named destinations out of 1000 (max. 500000)
 217376 words of extra memory for PDF output out of 221844 (max.
10000000)
```

Manuscript

Click here to access/download;Manuscript;Knowledge Mining in Biomedical Literature.pdf

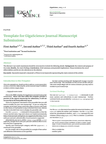

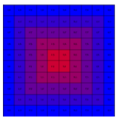

Journal of X, 2025, 1–14

Manuscript in Preparation
Research

RESEARCH

A Retrieval-Augmented Knowledge Mining Method with Deep Thinking LLMs for Biomedical Research and Clinical Support

Yichun Feng[1,2,3], Jiawei Wang[4], Ruikun He[5], Lu Zhou[3,*] and Yixue Li[2,3,6,7,8*]

[1]School of Advanced Interdisciplinary Sciences, University of Chinese Academy of Sciences, 100049 Beijing, China and [2]Key Laboratory of Systems Health Science of Zhejiang Province, School of Life Science, Hangzhou Institute for Advanced Study, University of Chinese Academy of Sciences, 310024 Hangzhou, China and [3]Guangzhou National Laboratory, No. 9 XingDaoHuanBei Road, Guangzhou International Bio Island, 510005 Guangzhou, China and [4]Department of EEIS, University of Science and Technology of China, 230026 Hefei, China and [5]BYHEALTH Institute of Nutrition & Health, 510663 Guangzhou, China and [6]GZMU-GIBH Joint School of Life Sciences, The Guangdong-Hong Kong-Macau Joint Laboratory for Cell Fate Regulation and Diseases, 511436 Guangzhou, China and [7]School of Life Sciences and Biotechnology, Shanghai Jiao Tong University, 200240 Shanghai, China and [8]Shanghai Institute of Nutrition and Health, Chinese Academy of Sciences Shanghai, 200030 Shanghai, China

[*]Correspondence address. Yixue Li, Guangzhou National Laboratory, No. 9 XingDaoHuanBei Road, Guangzhou International Bio Island, 510005 Guangzhou, China. E-mail: li_yixue@gzlab.ac.cn; Lu Zhou, Guangzhou National Laboratory, No. 9 XingDaoHuanBei Road, Guangzhou International Bio Island, 510005 Guangzhou, China. E-mail: zhou_lu@gzlab.ac.cn

Abstract

Background. Knowledge graphs and large language models (LLMs) are key tools for biomedical knowledge integration and reasoning, facilitating structured organization of scientific articles and discovery of complex semantic relationships. However, current methods face challenges: knowledge graph construction is limited by complex terminology, data heterogeneity, and rapid knowledge evolution, while LLMs show limitations in retrieval and reasoning, making it difficult to uncover cross-document associations and reasoning pathways.
Results. We propose a pipeline that uses LLMs to construct a biomedical knowledge graph (BioStrataKG) from large-scale articles and builds a cross-document question-answering dataset (BioCDQA) to evaluate latent knowledge retrieval and multi-hop reasoning. We then introduce Integrated and Progressive Retrieval-Augmented Reasoning (IP-RAR) to enhance retrieval accuracy and knowledge reasoning. IP-RAR maximizes information recall through Integrated Reasoning-based Retrieval and refines knowledge via Progressive Reasoning-based Generation, using self-reflection to achieve deep thinking and precise contextual understanding. Experiments show that IP-RAR improves document retrieval F1 score by 20% and answer generation accuracy by 25% over existing methods.
Conclusions. The IP-RAR helps doctors efficiently integrate treatment evidence to inform the development of personalized medication plans and enables researchers to analyze advancements and research gaps, accelerating the hypothesis generation phase of scientific discovery and decision-making.

Key words: Large Language Model, Knowledge Graph, Knowledge Mining, Retrieval-Augmented Generation, Deep Thinking

Compiled on: August 21, 2025.
Draft manuscript prepared by the author.

Key Points

- We construct the Biomedical Stratified Knowledge Graph (BioStrataKG) from large-scale research articles, which combines an entity-level graph with a document-level graph to support rich biomedical relationship discovery.
- We introduce BioCDQA — a biomedical cross-document question answering dataset built upon BioStrataKG — to address the limitations of existing QA datasets.
- We propose the IP-RAR framework, which combines Integrated Reasoning-based Retrieval with Progressive Reasoning-based Generation, enhanced by a self-reflection mechanism and LLMs with deep-thinking capabilities, to improve the accuracy and contextual relevance of answer generation.
- Experimental results show that IP-RAR consistently outperforms existing methods across all evaluation metrics, achieving state-of-the-art performance.
- The proposed framework supports a variety of downstream biomedical applications, including analysis for drug synergy and antagonism, generating hypotheses for drug repurposing, providing evidence for precision medicine, and assisting in scientific knowledge discovery, ultimately assisting clinical decision-making and accelerating biomedical research.

Introduction

The advancement of large language models (LLMs) has significantly accelerated progress in natural language processing (NLP), particularly in complex tasks like question answering (QA), with notable breakthroughs in specialized fields such as biomedical science [1]. However, despite these advancements, the application of LLMs for knowledge mining in specialized domains like biomedical science remains limited, where rigorous precision and robust evidence validation are essential for extracting meaningful insights [2]. Biomedical articles is vast, containing massive amounts of information [3], much of which remains underutilized. This inefficiency hinders scientific discovery and limits our ability to address complex biomedical challenges that require deep connections and advanced reasoning [4]. The wealth of knowledge within biomedical research, if effectively harnessed, could drive breakthroughs in critical areas such as clinical medicine, pharmacology, and molecular biology [5].

In biomedical knowledge mining, the construction of knowledge graphs is essential for enhancing information integration and reasoning capabilities. However, this process presents several challenges. First, the biomedical domain has a highly complex terminology system, with numerous synonyms, polysemous terms, and hierarchical classifications, making the precise extraction of entities and relationships particularly difficult [6]. Second, biomedical knowledge is fragmented and heterogeneous, spanning research papers, clinical reports, and databases, posing a fundamental challenge in unifying and linking these diverse data sources effectively [7]. Additionally, the rapid evolution of biomedical research constantly introduces new findings, requiring knowledge graphs to be dynamically expandable to maintain long-term relevance [8]. Despite these challenges, high-quality knowledge graphs can explicitly capture the intricate relationships among genes, proteins, diseases, and drugs, providing structured support for cross-document reasoning and deep knowledge extraction [9].

Building a high-quality biomedical QA dataset that effectively leverages knowledge graphs is crucial for evaluating and enhancing models' abilities in cross-document reasoning and knowledge integration. While existing datasets such as MASH-QA [10], which addresses multi-span questions across long documents, BioASQ [11], which focuses on biomedical semantic indexing and QA, MEDHOP [12], which targets multi-hop reasoning across multiple paragraphs, MedicationQA [13], which enhances understanding of medication-related queries, MedMCQA [14], which provides large-scale multiple-choice QA for medical examinations, and PcQA [15], which facilitates structured knowledge graph question answering, offer valuable benchmarks, they often fail to capture the deep, interconnected knowledge hidden within multiple sources.

Retrieval-Augmented Generation (RAG) has emerged as a promising approach for biomedical knowledge mining by integrating external knowledge into the response generation process [16]. However, its effectiveness relies on refining the retrieval mechanism to focus on high-quality, contextually relevant content, ensuring more accurate and reliable knowledge extraction [17]. Against this backdrop, advanced retrieval models like Contriever [18] have provided a powerful foundation for RAG systems by using unsupervised contrastive learning to learn high-quality text representations without relying on labeled data. SELF-RAG [19] dynamically adjusts the retrieval process by evaluating the quality of the retrieved content, thereby enhancing the accuracy of long-form answers in complex domains like biomedical research. Other innovative approaches include GraphRAG [20], which structures retrieved passages into graphs to capture relationships between them, improving coherence and relevance. CRAG [21] further refines the process by using a lightweight evaluator to assess retrieved passages and triggering new, web-based corrective searches for irrelevant documents, thereby actively improving the quality of the knowledge source before generation. Additionally, RAPTOR [22] organizes retrieval results into a tree structure, recursively summarizing data to facilitate more effective reasoning. Further extending this paradigm, agent-based frameworks like MedAgents[23] structure LLMs into a collaborative group of experts that can collectively reason and utilize tools to solve complex medical problems.

In addition to extracting high-quality information from external knowledge bases using RAG, LLMs have recently made breakthrough progress in their inherent reasoning capabilities. For instance, OpenAI's GPT-o1 leverages reinforcement learning and a chain-of-thought mechanism to demonstrate reasoning on complex tasks in mathematics, programming, and science—achieving performance comparable to that of PhD-level experts [24]. Meanwhile, DeepSeek has released its reasoning-oriented model, DeepSeek-R1 [25], which performs on par with GPT-o1 in logical reasoning, mathematical computation, and code generation, yet its training cost is only a small fraction of that of GPT-o1. These breakthroughs not only expand the functionalities of large models but also provide stronger intrinsic support for complex domains such as biomedical knowledge mining, helping to yield more accurate answers in cross-document and multi-layer relational reasoning tasks.

In this paper, we present a comprehensive framework for biomedical knowledge mining, as illustrated in Figure 1. This framework processes diverse biomedical knowledge sources, such as research papers and user queries, through a systematic pipeline that involves constructing knowledge graphs and generating precise responses to user queries. Within this pipeline, LLMs collaborate with retrieval-augmented reasoning techniques to extract, organize, and integrate domain-specific knowledge. First, an entity-level knowledge graph and a document-level knowledge

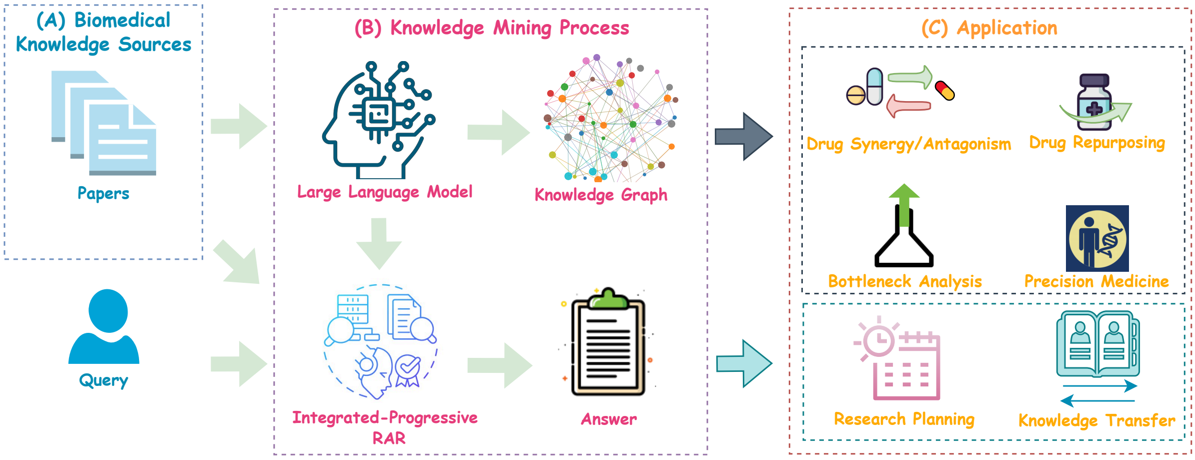

Figure 1. Overview of the proposed framework for biomedical knowledge mining. (A) A Biomedical Knowledge Source, consisting of a large corpus of research papers, provides the foundational knowledge for the system. A user's query serves as the direct input to trigger the question-answering process. (B) A knowledge mining pipeline leverages a LLM to build a knowledge graph from the papers and utilizes the IP-RAR approach to generate a precise answer. (C) The outputs from the pipeline enable diverse applications: the generated Knowledge Graph supports analytical tasks such as generating hypotheses for drug repurposing and analysis for drug synergy/antagonism, while the precise Answers from the QA process facilitate applications such as supporting research planning and facilitating knowledge transfer.

graph are constructed from large-scale biomedical research papers and merged into the Biomedical Stratified Knowledge Graph (BioStrataKG). The entity-level graph captures relationships between biomedical entities such as genes, proteins, diseases, and drugs, while the document-level graph represents connections between research papers based on shared methods, datasets, and research directions, as well as citation and reference relationships. Together, these knowledge graphs uncover complex biomedical relationships and reveal latent patterns, thereby supporting advanced knowledge discovery. Since existing datasets do not meet the requirements for cross-document reasoning and knowledge mining, we develop a new biomedical cross-document question answering dataset, called BioCDQA, based on the BioStrataKG to support these tasks. Second, we propose the Integrated and Progressive Retrieval-Augmented Reasoning (IP-RAR) framework, which synergizes Integrated Reasoning-based Retrieval with Progressive Reasoning-based Generation to seamlessly integrate relevant knowledge, enabling more precise and contextually relevant reasoning. The framework first maximizes the recall of pertinent information from large-scale biomedical articles through Integrated Reasoning-based Retrieval, ensuring comprehensive coverage of relevant data. Subsequently, the progressive reasoning-based generation mechanism refines and enhances the extracted knowledge while leveraging a self-reflection mechanism to continuously optimize the accuracy and contextual relevance of the answers. Ultimately, LLMs with deep thinking capability further refine the reasoning process to derive a high-quality answer. The IP-RAR framework enables precise reasoning and knowledge integration, providing effective technical support for a wide range of downstream biomedical research applications, including analysis to support drug synergy/antagonism studies, generating hypotheses for drug repurposing, and informing precision medicine strategies. This framework helps doctors quickly identify and integrate relevant treatment evidence from vast biomedical articles, enabling more precise personalized medication plans. It also allows researchers to systematically analyze cutting-edge advancements and potential research gaps, accelerating research strategy formulation and decision-making. These advancements in knowledge mining pave the way for more efficient and accurate solutions in contemporary biomedical research.

Materials and Methods

Construction of the BioStrataKG

This section introduces the construction method of the BioStrataKG, proposing a document-entity dual-layer representation fusion architecture based on LLMs, which effectively captures complex relationship networks among biomedical entities and establishes a cross-document knowledge association system based on this network. As shown in Figure 2a, the construction process of BioStrataKG begins with large-scale biomedical articles, utilizing GPT-4o mini [26] for fine-grained knowledge extraction, including entity-relationship triple extraction and the structured representation of semantic information such as research methods and fields in the article. Subsequently, cross-document association networks are established through entity co-occurrence analysis, achieving a hierarchical expansion of the knowledge graph from micro-level entity relationships to macro-level document associations. The entity types and relationship types within the knowledge graph are detailed in Figure 2b.

Data Collection and Processing

Paper Collection. The papers included in our dataset are all sourced from the open-access database PubMed, which allows anyone to download and access biomedical and life sciences article via PMID. We have downloaded over 8,000,000 papers, extracting their titles, abstracts, publication years, keywords, and citation relationships.
Paper selection. We filtered papers based on keywords related to lung cancer, breast cancer, and colorectal cancer, focusing on studies involving single-cell analysis, pharmacology, and clinical trials. We further narrowed our selection to papers published within the last decade and those with more than 10 citations. In total, we selected 15,585 papers to build the knowledge graph for generating the bioCDQA dataset. Some of the basic statistics of the papers involved in our dataset are detailed in Supporting Information Fig. S2.
PDF to Markdown Conversion. While we initially attempted to use XML files, we found that for a portion of the open-access literature, the XML versions contained only metadata and abstracts, with the full text being either missing or poorly formatted. As a key objective of our study is full-text knowledge mining, the PDF format was essential for obtaining the most complete information. Therefore, we adopted a hybrid approach, retaining available metadata and abstracts while processing the complete full text from the corre-

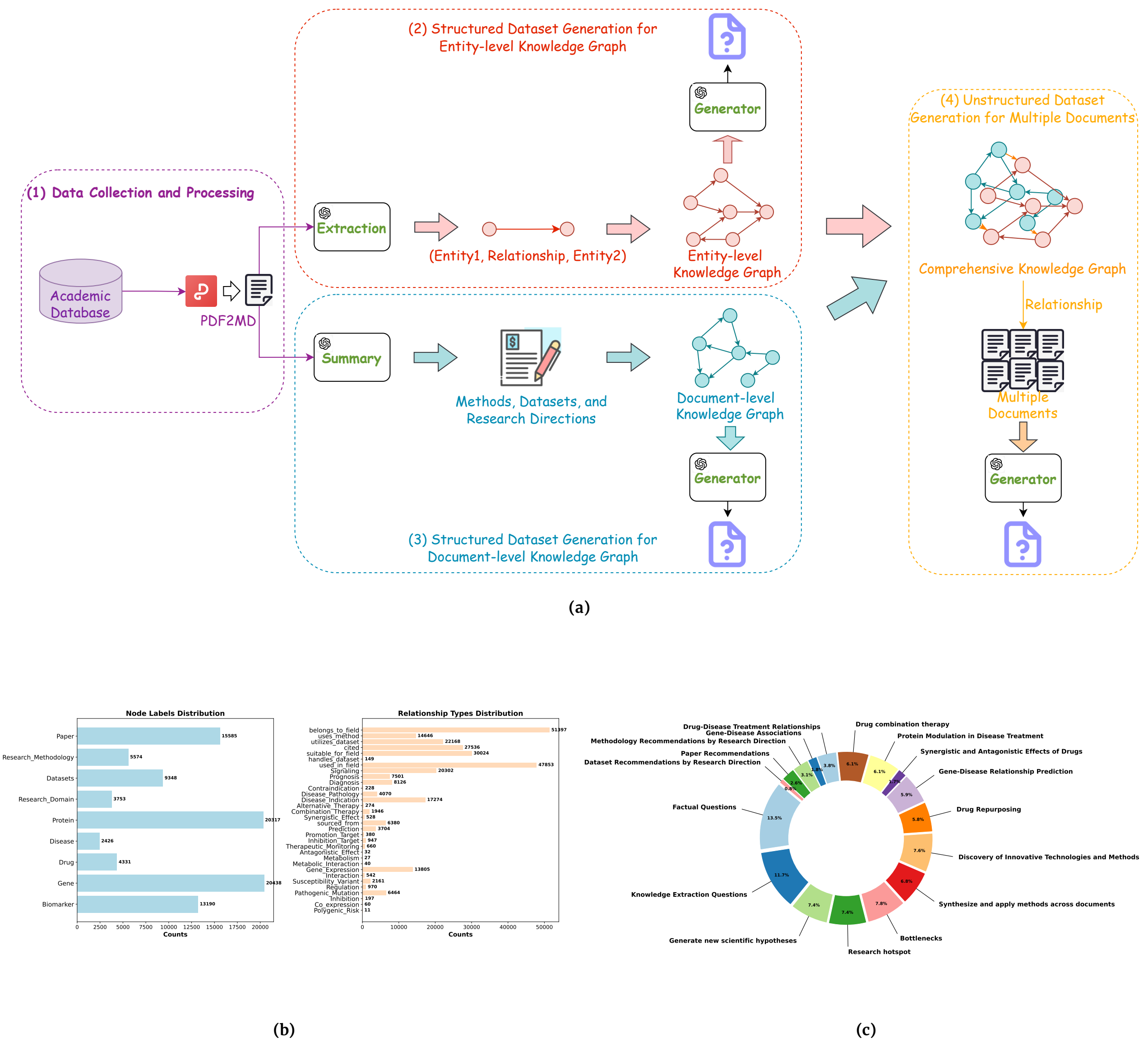

Figure 2. Construction Pipeline and Statistical Analysis of the Dataset. (a) BioStrataKG and BioCDQA Construction Workflow Diagram. (1) Data Collection and Processing: The process begins by converting research papers from PDF to markdown (MD) format to facilitate content extraction. (2) Structured Dataset Generation for Entity-level Knowledge Graph: An LLM is used to extract entities and relationships (Entity1, Relationship, Entity2), which are then standardized to construct an entity-level knowledge graph. This graph supports downstream tasks such as generating hypotheses for drug repurposing, drug interaction analysis for comorbid conditions , and gene-disease associations. (3) Structured Dataset Generation for Document-level Knowledge Graph: Summarization is performed using an LLM to extract key aspects such as methods, datasets, and research directions. The resulting document-level knowledge graph facilitates tasks such as research strategic planning and research paper recommendations. (4) Unstructured Dataset Generation for Multiple Documents: Integration of the entity-level and document-level knowledge graphs produces a comprehensive knowledge graph. This integrated graph enables connections across multiple documents and supports downstream tasks such as content-based factual questioning, knowledge transfer, trend analysis, and hotspot detection. (b) Statistics of node labels and relationship types in BioStrataKG. (c) Statistics of question categories in BioCDQA.

sponding PDFs to ensure comprehensive data coverage. Since the papers downloaded from the database are in PDF format, we convert them to Markdown format using the marker toolkit [27].

Knowledge Graph Construction

We leverage the GPT-4o mini to extract biomedical-related triplet information (e.g., genes, proteins, diseases, drugs, etc.) from research papers to construct an entity-level knowledge graph. Simultaneously, we structurally summarize the research methods, datasets used, and research domains of the papers to build a document-level knowledge graph. On this basis, each paper serves as a node within the document-level knowledge graph. By identifying recurring biomedical terms, related datasets, research methods across multiple papers, and analyzing citation and reference relationships, we establish inter-document linkages and achieve comprehensive knowledge integration. This cross-document information connection not only facilitates the construction of high-quality cross-document question-answering datasets but also provides richer contexts and deeper opportunities for knowledge discovery in biomedical knowledge mining tasks. Additionally, we have open-sourced this knowledge graph, which can be accessed at https://github.com/yichun10/BioCDQA.git. Beyond supporting our dataset, the knowledge graph can serve as a valuable resource for researchers, aiding in knowledge discovery, identifying trends, and exploring relationships between biomedical entities and research methods. The knowledge graph contains

94,962 nodes and 290,403 relationships. Detailed descriptions of the nodes and relationships are provided Figure 2b. The knowledge graph has an average degree of 6.11, and its node degree distribution is provided in Supporting Information Fig. S3.

Entity-Level Knowledge Graph

Triplet Extraction Based on LLMs. In the KG, a triplet is the fundamental unit of information used to represent entities and their relationships. Specifically, a triplet is defined as (e_1, r, e_2), where e_1 and e_2 are entities, representing nodes in the knowledge graph, and r is a relation, representing the connection between the entities. We utilize the GPT-4o mini to extract triplets from the abstracts of papers. The entity types (ET) we extract are Gene, Protein, Drug, and Disease. The relation types (RT) we define and Triplet Extraction Prompt are detailed in Supporting Information Fig. S4.

Entity Normalization in Triplet Extraction. Entity normalization is essential for ensuring consistent and standardized representation of extracted entities in knowledge graph construction. To address this, we adopt a two-stage approach that combines domain-specific databases with advanced language models. First, we reference the MeSH database [28] for diseases and drugs, and the UniProt database [29] for genes and proteins. Using the all-MiniLM-L6-v2 [30] embedding model, we retrieve the top 5 candidate terms from these databases by generating dense vector representations of the extracted entities, ensuring computational efficiency and high recall. Next, GPT-4o mini evaluates the semantic and contextual alignment between the extracted entity and the retrieved candidates to identify the most appropriate standardized term. This approach resolves ambiguities, such as homonyms or abbreviations, and ensures that entities are contextually accurate and semantically consistent.

Constructing Entity-Level Knowledge Graphs. Once the standardized triplets (e_1, r, e_2) are obtained, the next step is to construct the entity-level knowledge graph, where entities e_1 and e_2 are represented as nodes and the relation r forms the directed edge between them. The process begins with node creation, where each unique entity from the standardized triplets is instantiated as a node in the graph and categorized based on its type (e.g., Gene, Protein, Drug, Disease) to ensure consistency and facilitate downstream analysis. Relations are then represented as directed edges, labeled with their relation types, connecting the corresponding nodes and formalizing the interactions between entities. In practice, every relationship in the knowledge graph includes a key attribute: the Evidence Source, which is the ID of the original paper supporting the relationship. This attribute is fundamental for ensuring that every relationship in the graph is traceable to its origin. The graph supports multiple labeled edges between the same nodes, capturing diverse relationships such as a gene being associated with multiple diseases, and manages complexity by representing each distinct relationship as a separate edge. During the construction process, rigorous checks are applied to detect and handle duplicate nodes and edges, ensuring that new relationships are accurately linked to existing nodes, thereby preventing redundant entries. The completed KG is stored in the Neo4j graph database, where Neo4j's Cypher query language enables efficient and intuitive exploration of the graph. Users can retrieve complex relationship networks, such as querying all drugs related to a specific disease or identifying potential relationship chains between entities, facilitating flexible and powerful knowledge discovery.

Document-Level Knowledge Graph

Paper Information Extraction Based on LLMs. We utilize the GPT-4o mini to extract the fundamental research methods of each paper, the datasets used, and the respective research domains. These extracted elements, along with the paper titles, are considered as nodes. Each node and its relationship constitute a document-level triplet (e_1, r, e_2), facilitating subsequent knowledge graph construction. The prompt for extraction of paper information is presented in Supporting Information Fig. S5.

Normalization. To achieve standardization and consistency of entity names in document-level knowledge graphs, we propose a standardization workflow based on vector matching and LLM-assisted decision-making. The workflow processes each entity name in the triples sequentially, ensuring that all potential duplicate entities are incorporated into subsequent matching and standardization steps. For each entity name, the existing vector database (e.g., ChromaDB) is traversed to calculate the cosine similarity with other entities. If a record with a similarity score greater than 0.5 is found, the two entity names are considered to refer to the same concept (e.g., a method or dataset), and the current entity name is merged with the existing record. Conversely, if no matching entity name is found, the current entity name is inserted into the vector database for use in future standardization steps. Building on the initial steps, GPT-4o mini further optimizes merged entity names by identifying the most semantically relevant and contextually accurate choices, ensuring standardization, precision, and robustness in the final output.

Constructing Document-Level Knowledge Graphs from Triplets. After normalizing document-level triplets (e_1, r, e_2), the document-level knowledge graph is constructed in a manner similar to the entity-level KG. Each unique entity, including papers, research methods, datasets, and research domains, is instantiated as a node in the graph. Directed edges are created between these nodes to represent the relationships, ensuring an efficient and comprehensive structure for knowledge discovery.

Construction of the Biomedical Cross-Document Question Answering Dataset

Based on BioStrataKG, we introduce a biomedical question-answering dataset—BioCDQA, designed to support cross-document reasoning and biomedical knowledge mining. The construction process of the dataset is illustrated in Figure 2a. The dataset integrates data from both unstructured text and structured knowledge graphs. By extracting information from text, we obtain rich contextual data, while the knowledge graph provides precise relationships between entities such as genes, diseases, drugs, and proteins. These data sources collectively ensure the diversity and practicality of the dataset. This dataset consists of tuples containing the following elements: question, question type, answer, source papers for the answer, and source sentences for the answer. Each tuple includes a natural language question, whose answer is composed of one or more sentences extracted from the source papers. These answers may originate from a single paper or multiple papers and can consist of multiple sentences, either from a single span or dispersed across different sections of various source papers.

The dataset contains a total of 1,183 question-answer pairs, covering 68,428 papers and providing over 1.85 million document chunks available for retrieval. To create this large-scale retrieval corpus, we employed a text chunking strategy. We utilized a Recursive Character Text Splitter to segment the full text of the papers, setting the chunk size to 500 characters with a 100-character overlap between consecutive chunks. This method was chosen for its effectiveness in attempting to preserve sentence integrity when splitting long texts, thereby maximally maintaining local contextual coherence and reducing information loss from improper segmentation. The dataset and the corresponding set of retrievable papers are available at https://github.com/yichun10/BioCDQA.git. The distribution of question types is shown in Figure 2c. The definitions and characteristics of each type are elaborated in the subsequent sections.

Dataset Generation from Unstructured Data

We employ the BioStrataKG to establish relationships among multiple research papers. Randomly selecting 1 to 5 interconnected articles, we input the full text of the chosen papers into GPT-4o mini. This system then formulates pertinent questions from the following perspectives and generates responses based on both the questions and the text.

Factual Questions. In the domain of biology, factual questions typically revolve around specific biological facts, data, or phenomena. Examples include inquiries about the sequence of a particular gene, the function of a specific protein, epidemiological data of a disease, or the detailed steps of a biological process. These questions can be answered through a thorough examination of scientific literature, experimental data, or validated biological databases, ensuring responses are grounded in objective, verifiable information rather than subjective opinions.

Knowledge Extraction Questions. The primary objective of knowledge extraction questions is to identify and extract specific categories of information from a given text, typically presented in a list format. In the biomedical domain, such questions may involve extracting names of drugs, genes, diseases, or symptoms. For instance, when asked, "What are the drugs used to treat lung cancer?", the system must extract relevant drug names from the text and present them in a list, typically identifying three to five commonly used drugs. The focus of these questions is on precise extraction rather than interpretation or reasoning. The goal is to quickly distill key entity information from large volumes of unstructured text, enabling researchers to efficiently retrieve the necessary biomedical data and accelerate literature analysis.

Knowledge Discovery Questions. Knowledge discovery questions represent a key feature of our dataset, aiming to extract deeper insights and foster innovative scientific inquiry. These questions not only facilitate the generation of new hypotheses from the findings and conclusions presented in the literature but also assist in designing subsequent research plans. By analyzing existing literature, these questions help identify hidden themes and emerging research hotspots, providing valuable insights into current trends. Additionally, they empower researchers to analyze publication trends, assess keyword relevance, and evaluate citation metrics, uncovering critical areas of interest and guiding potential future directions in biomedical research. Moreover, knowledge discovery questions allow for the identification of bottlenecks within specific fields, based on comprehensive reviews, which highlights pressing challenges that need addressing. Through the synthesis of information across multiple documents, these questions support the transfer of methodologies and concepts to other research areas, fostering interdisciplinary innovation. Overall, knowledge discovery questions play a pivotal role in advancing the scientific dialogue by tracking the evolution of knowledge, revealing critical nodes in research development, and ultimately guiding future investigations in the biomedical domain.

Dataset Generation from Structured Data

We generate various Cypher query statements based on BioStrataKG, which features two distinct granularity-based knowledge graphs (entity-level and document-level), to extract relevant subgraphs and relationship chains. The Entity-Level Knowledge Graph provides a comprehensive framework for exploring complex biomedical interactions, such as those between diseases, genes, drugs, and proteins. This supports key research areas, including drug repurposing, gene-disease prediction, and treatment optimization. Meanwhile, the Document-Level Knowledge Graph is designed to enhance research efficiency by offering tailored recommendations aligned with specific research directions. These queries retrieve nodes and relationships pertinent to specific research topics, enabling the extraction of focused subgraphs and relationship chains. By leveraging these, GPT-4o mini formulates targeted questions and generates corresponding answers, improving question precision and dataset quality.

Drug Repurposing. Triplet relationships between diseases, genes, and drugs are established to uncover new applications for existing drugs in treating previously unrelated diseases. By leveraging data on drug effects and identifying hidden connections, innovative treatment pathways are discovered.

Gene-Disease Relationship Prediction. Known associations between genes and diseases are analyzed to predict new gene-disease relationships. Patterns and similarities within the data highlight potential genetic markers involved in disease development, aiding early diagnosis and targeted therapies.

Synergistic and Antagonistic Effects of Drugs. Drug interactions are examined with a focus on synergistic (enhancing) or antagonistic (counteracting) effects. For patients with comorbid conditions, harmful interactions are identified, and safer alternatives are suggested, improving patient safety and optimizing treatment effectiveness.

Protein Modulation in Disease Treatment. The inhibitory or promotive effects of proteins on disease management are analyzed, providing insights into protein-drug interactions and supporting the design of effective therapeutic strategies.

Drug combination therapy. Drug combination therapies are analyzed to optimize dosages and interactions for treating complex conditions, ensuring maximum therapeutic benefits with minimal side effects.

Drug-Disease Treatment Relationships. Relationships between specific drugs and the diseases they treat are explored to gain insights into effective treatment options and potential new therapeutic applications for existing drugs.

Gene-Disease Associations. Comprehensive information on gene-disease associations reveals genetic predispositions, enabling the development of genetic tests for early detection and personalized treatments based on individual genetic profiles.

Dataset Recommendations by Research Direction. Metadata and literature are analyzed to identify and recommend datasets that align with specific research goals, providing high-quality, curated resources tailored to researchers' needs.

Methodology Recommendations by Research Direction. Suitable research methodologies are recommended based on the identified direction, equipping researchers with effective approaches ranging from traditional techniques to emerging methods.

Paper Recommendations by Research Direction. Relevant papers are suggested based on research direction, including seminal works, recent publications, and highly cited studies, enabling researchers to build on existing knowledge and stay updated on advancements in their field.

Dataset Inspection with LLM

Leveraging both unstructured and structured data, we utilize the capabilities of GPT-4o mini to generate a substantial corpus of over 5,000 question-answer pairs. To verify the reliability of the generated answers, we then employ GPT-4o. Given the original source articles, we design prompts for GPT-4o to identify the specific passage within the article that supports each QA pair. Dataset Inspection Prompt are detailed in Supporting Information Fig. S6.

Manual Inspection and Selection

To ensure the absolute reliability of the BioCDQA dataset, we implemented an exceptionally rigorous manual curation and selection protocol. This process was independently conducted by two authors with biomedical backgrounds, who filtered an initial pool of over 5,000 machine-generated QA pairs down to the final 1,183 high-quality entries. The reviewers evaluated each pair against a comprehensive set of criteria, including: Factual Correctness and Faithfulness, to ensure every assertion in the answer was clearly supported by and traceable to the source documents and free of

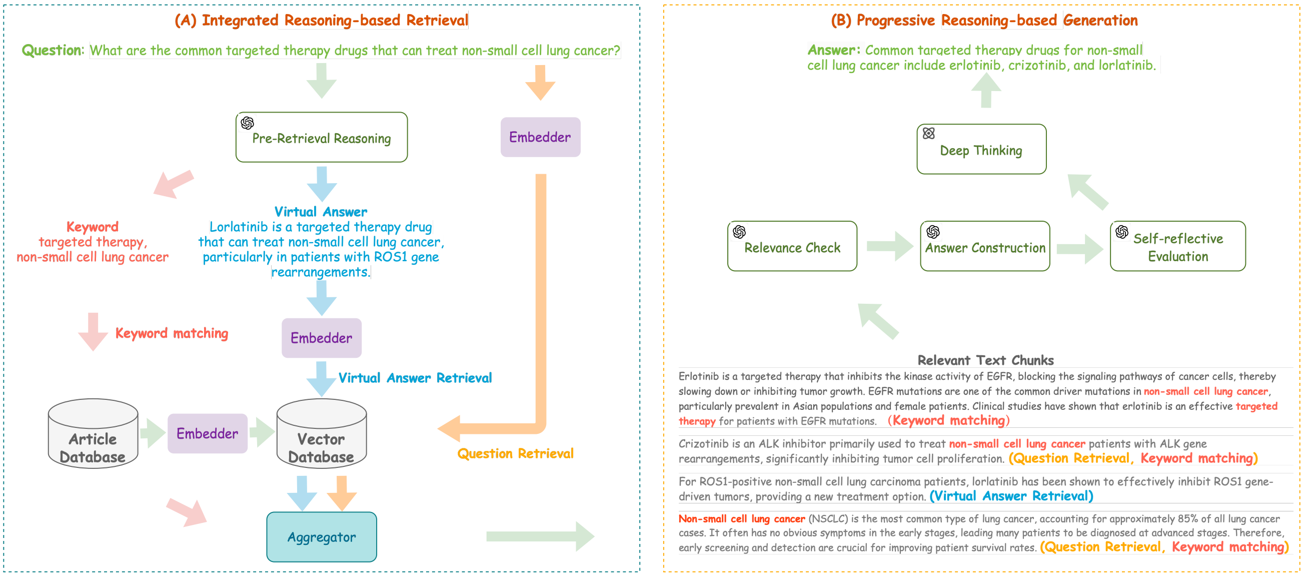

Figure 3. Framework of IP-RAR. (A) Integrated Reasoning-based Retrieval: Performs pre-retrieval reasoning, extracting keywords and generating a virtual answer. Then, a multi-level, multi-granularity retrieval strategy is used to retrieve relevant text chunks, which are ranked based on relevance. (B) Progressive Reasoning-based Generation: This stage begins with the Relevant Text Chunks retrieved from the process in (A). The labels next to each chunk (e.g., (Keyword matching), (Question Retrieval), (Virtual Answer Retrieval)) indicate which retrieval strategy sourced that piece of evidence.
Then, filters out irrelevant text chunks through explanations or self-reflection, then leverages DeepSeek-R1 for deep-thinking-based reasoning on the valid text chunks, generating a precise final response.

hallucinations; Completeness, to verify that no critical information was omitted; Relevance and Conciseness, to confirm the answer was focused and without redundant details; and the overall Question Quality (i.e., clarity and non-ambiguity). For the final selection, only those QA pairs that received consensus approval from both reviewers were retained. This high-quality dataset facilitates deep knowledge mining across a complex space of 68,428 research papers by providing contextually relevant questions linked to precise biomedical concepts for advanced reasoning.

Integrated and Progressive Retrieval-Augmented Reasoning Framework

In this study, we propose the IP-RAR framework, specifically designed for deep-thinking-based knowledge mining and question answering in large-scale biomedical articles. The overall framework is illustrated in Figure 3.

Integrated Reasoning-based Retrieval

Pre-Retrieval Reasoning. The first step employs the DeepSeek-V3 [31] to perform pre-retrieval reasoning by extracting key terms to identify relevant biomedical entities and generating synonyms to enhance matching flexibility. This approach overcomes the limitations of existing methods that struggle to precisely locate answers in complex queries, enabling more accurate targeting of specific biomedical entities. Furthermore, based on the pre-retrieval reasoning process, the system generates a virtual answer as a hypothesis to refine retrieval precision, improving the identification of relevant information. This improves the system's ability to link related paragraphs and retrieve contextually consistent information, with the prompt for pre-retrieval reasoning detailed in Supporting Information Fig. S7.

Multi-Level and Multi-Granularity Retrieval Strategy. To enhance recall and maximize the retrieval of relevant knowledge from millions of text chunks, we employ a retrieval strategy that integrates multi-level and multi-granularity approaches. The multi-level aspect combines abstract-based and full-text-based retrieval to capture relevant content across varying depths of detail. The multi-granularity aspect involves question-based, keyword-based, and virtual answer-based retrieval, ensuring a comprehensive exploration of the corpus. Using the Contriever-MS MARCO model [18], we first retrieve the top 10 abstracts based on the similarity between the question and text chunks, followed by another top 10 abstracts based on the similarity between the virtual answer and text chunks. At the full-text level, we retrieve the top 10 chunks for the question and an additional top 10 for the virtual answer using the same model. Additionally, we apply keyword-based matching techniques, expanding retrieval by including synonyms to increase flexibility. This multi-level and multi-granularity retrieval strategy effectively captures relevant content from different perspectives and depths, significantly improving the alignment of retrieved knowledge with the question's intent from a vast corpus.

Aggregator. The aggregator framework employs a weighted normalization function to rank retrieved chunks effectively. This function integrates three key factors: similarity score, method diversity, and intra-document repetition, ensuring contextually relevant and diverse rankings. Each factor is weighted according to its relevance, producing a normalized score S_i for each chunk, allowing for comparison across all retrieved chunks. The score for each chunk i is calculated as:

$$S_i = w_S \times \frac{S_{\text{sim},i}}{S_{\text{sim},max}} + w_M \times \frac{M_i}{M max} + w_R \times \frac{R_i}{R max} \qquad (1)$$

where $S_{\text{sim},i}$ represents the similarity score of chunk i, M_i indicates the number of distinct retrieval methods that identified chunk i, and R_i reflects the count of retrieved chunks from the same document as chunk i. The terms $S_{\text{sim,max}}$, $M\text{max}$, and $R\text{max}$ are the maximum values of these metrics across all chunks, used for normalization. The weights w_S, w_M, and w_R control the importance of each factor, where w_S emphasizes the relevance of similarity scores, w_M highlights chunks retrieved by multiple methods, and w_R prioritizes chunks with higher intra-document retrieval coverage. The resulting normalized score S_i provides a robust ranking metric that integrates query relevance, retrieval diversity, and intra-document coverage, providing a comprehensive and balanced ranking metric. In our experiments, these weights are set to $w_S = 5$, $w_M = 3$, and

$w_R = 1$.

Progressive Reasoning-based Generation

The Progressive Reasoning-based Generation process in our proposed IP-RAR framework is designed to ensure that only the most relevant evidence is employed in generating the final response. This process unfolds in four distinct phases:

Relevance Check. In this phase, all candidate text chunks are ranked in descending order based on their retrieval scores. The ranked chunks are then sequentially presented to the DeepSeek-V3, which evaluates whether each chunk sufficiently answers the posed question. This process continues until 5 relevant chunks are identified. If fewer than 5 relevant chunks are available, only those identified as relevant are used; if no relevant chunks are identified, the top 5 highest-scoring chunks are selected regardless of their assessed relevance.

Answer Construction. After selecting the candidate text chunks, a simple prompt is constructed to allow the DeepSeek-V3 to generate an initial response to the question. The prompt incorporates the context of the question and the selected chunks to facilitate a coherent and informed answer.

Self-Reflective Evaluation. Following the generation of the response, a self-reflection evaluation is performed to systematically assess the degree to which each text chunk supports the generated response. The DeepSeek-V3 is prompted to evaluate the relevance of each chunk with respect to the question and the proposed answer, assigning a support score to each. Scores are given on a scale from 0 to 100, where 100 represents maximum relevance, and 0 indicates that the chunk is either entirely irrelevant or contradicts the proposed answer. Intermediate scores reflect varying levels of support based on the chunk's contribution to the response.

Deep Thinking. Based on the support scores assigned during the self-reflective evaluation, the system prioritizes the text chunks corresponding to the most relevant answers and utilizes DeepSeek-R1 [25] to perform deep-thinking-based reasoning for the final answer generation. The DeepSeek-R1 is prompted to integrate these highly relevant text chunks, producing a cohesive and precise final response to ensure both contextual consistency and comprehensive accuracy.

Results

Results of Various Approaches on the BioCDQA Dataset

For the BioCDQA dataset, we design a set of evaluation metrics. Document retrieval performance is assessed using Mean Precision, Mean Recall, and Mean F-measure to evaluate retrieval accuracy and completeness. Answer accuracy evaluation relies on a GPT-4-based five-point scoring system, ensuring a comprehensive assessment of the semantic consistency and precision of generated answers. This approach is particularly suitable for summary-type question-answering tasks, where traditional metrics struggle to provide stable quality measurements. A detailed introduction to the evaluation metrics can be found in the Supporting Information section 1.1.

As shown in Table 1, we evaluated IP-RAR against two distinct categories of methods: non-RAG and RAG-based. The non-RAG methods—Chain of Thought (CoT), Few-Shot, and MedAgents, all utilizing the DeepSeek-V3 model—rely solely on the LLM's internal, parametric knowledge. The results clearly demonstrate the limitations of this approach for our evidence-based, cross-document task; without the support of external data, all non-RAG methods achieve significantly lower answer scores. Among them, MedAgents performs best. This is likely because its multi-agent collaborative framework can more effectively extract the latent medical knowledge within the base LLM. However, its performance is still fundamentally capped by the lack of retrieved, context-specific evidence.

In contrast, RAG-based methods show a marked improvement. In the document retrieval stage, IP-RAR achieves the highest F1-score, demonstrating a superior balance between Precision and Recall. This high-fidelity retrieval is crucial for downstream performance. For instance, while SELF-RAG attains the highest recall, its extremely low precision indicates that it retrieves a large volume of irrelevant noise, which significantly hinders its answer generation performance. Similarly, CRAG's modest retrieval quality leads to a lower answer score. This highlights the importance of high-quality retrieval, which directly contributes to IP-RAR's state-of-the-art answer score of 76.41%. The overall results confirm that a RAG-based approach is essential for this task and that within this paradigm, IP-RAR's sophisticated architecture comprehensively outperforms all tested baseline systems.

In addition to retrieval and generation accuracy, we analyzed the inference time and GPU memory usage of the IP-RAR framework to evaluate its practical feasibility, conducting a direct comparison against the SELF-RAG baseline. The analysis was conducted on a single NVIDIA A100 GPU against our retrieval corpus of over 1.85 million document chunks. For a typical query, IP-RAR required an average of 26.7 seconds for a complete response, with a peak GPU memory usage of only 3GB. In contrast, SELF-RAG was slower, with an average time of 32.4 seconds, and required a substantially larger peak memory of 78GB. These results demonstrate that IP-RAR is not only faster but also orders of magnitude more resource-efficient. This comprehensive efficiency, especially when compared to the hours a human expert might need for manual synthesis, proves that the performance advantages of IP-RAR do not come at an impractical computational cost.

Performance of IP-RAR on Different Datasets

For the BioASQ and MASH-QA datasets, we adhere to the evaluation criteria established by their respective datasets.

BioASQ dataset

In our evaluation on the BioASQ dataset, IP-RAR is benchmarked against several top-performing systems from the challenge, which serve as robust baselines. The first baseline, dmiip3, combines BM25 and GPT-3.5 for initial retrieval, employs a cross-encoder re-ranker based on biomedical pretrained models like PubMedBERT, and finally generates answers through GPT-3.5. Another strong baseline, bioinfo-0, adopts a multi-stage pipeline: it begins with traditional BM25 retrieval, then enhances results using Transformer-based neural re-rankers such as PubMedBERT. For the BioASQ dataset, Table 2(a) demonstrates the superior document retrieval performance of IP-RAR. With higher precision and F1-score, IP-RAR effectively filters out irrelevant documents, while its leading performance in MAP and GMAP highlights its strength in ranking relevant documents. In contrast, although SELF-RAG achieves a higher recall, IP-RAR's precision-focused trade-off results in better overall retrieval quality. Methods like bioinfo-0 and dmiip3 improve recall but perform worse in precision and ranking, often retrieving excessive irrelevant content.

It is noteworthy that both IP-RAR and SELF-RAG employ similar workflows: an ideal answer is first generated through their respective systems, followed by refinement using DeepSeek-V3 to extract the final response tailored to the specific question type. For these two systems, all evaluations are conducted on the extracted answers to ensure consistency and comparability. Other methods, such as dmiip3 and BioASQ Baseline, are evaluated based on their original outputs to maintain fairness and a consistent basis for comparison. Table 2(b) presents a comparison of exact answer results across IP-RAR and other methods. For Yes/No questions, IP-RAR achieves an F1 score and accuracy close to 96%, second only to IISR-2. In Factoid questions, it ranks first with a Strict Accuracy of 68.36%

System	Document Retrieval			Answer
	Prec. (%)	Rec. (%)	F1 (%)	GPT-4 Eval (%)
**Non-RAG Methods**				
CoT	/	/	/	21.83
Few-Shot	/	/	/	15.62
MedAgents	/	/	/	38.89
**RAG-based Methods**				
Contriever+Llama 3 8B	24.53	19.40	21.67	25.86
CRAG	12.18	18.49	14.69	29.27
SELF-RAG	7.12	**31.28**	11.59	41.16
**IP-RAR (ours)**	**47.18**	27.76	**34.96**	**76.41**

Table 1. Comparison of IP-RAR against non-RAG and RAG-based methods on the BioCDQA dataset. The best results are highlighted in bold.

System	P (%)	R (%)	F1 (%)	MAP (%)	GMAP (%)
A&Q4	10.27	58.16	17.46	44.04	2.15
dmiip3	11.33	61.27	19.12	44.62	2.40
bioinfo-0	21.18	60.47	31.37	45.90	2.67
SELF-RAG	49.82	**74.53**	59.72	89.33	22.15
**IP-RAR**	**85.87**	57.95	**69.20**	**95.12**	**35.66**

(a)

System	Yes/No		Factoid		List		
	F1	Acc.	Str. Acc.	MRR	Prec.	Rec.	F1
Baseline	60.00	46.67	9.09	11.36	11.85	27.84	16.13
dmiip3	85.71	87.30	31.82	39.92	28.51	24.64	22.32
UR-gpt4	94.74	95.64	54.55	56.82	37.42	43.69	38.28
IISR-2	**100.00**	**100.00**	54.55	59.09	50.99	35.77	39.80
SELF-RAG	85.14	82.55	30.61	28.31	51.10	22.38	29.19
**IP-RAR**	95.91	95.34	**68.36**	**67.34**	**88.78**	**54.53**	**63.04**

(b)

Table 2. Comparison of document retrieval and exact answer results between IP-RAR and commonly used methods on the BioASQ dataset. (a) Document retrieval performance comparison. (b) Exact answer generation performance comparison. The best results are highlighted in bold.

and an MRR of 67.34%. For List questions, IP-RAR outperforms all systems with the highest Precision, Recall, and F1, demonstrating superior retrieval completeness.

In summary, IP-RAR outperforms other methods on the BioASQ dataset in both document retrieval and exact answer tasks, demonstrating superior precision, recall, ranking, and comprehensiveness.

MASH-QA dataset

Table 3 highlights IP-RAR's superior performance on the MASH-QA dataset. For sentence retrieval, it achieves the highest F1 score (64.44%) with a strong balance of Precision and Recall, outperforming SELF-RAG, which suffers from low Precision (27.46%) despite its high Recall.

In answer prediction, IP-RAR's Exact Match (47.29%) more than doubles that of MultiCo, demonstrating superior accuracy. Notably, since both IP-RAR and SELF-RAG rely on LLMs to generate answers, the EM metric was evaluated using a GPT-4o-based [32] scoring system, which assigns a score of 1 only when the predicted answer and the gold standard convey the exact same meaning without any extraneous sentences. Any discrepancy in meaning or the inclusion of irrelevant sentences results in a score of 0. This strict scoring criterion highlights IP-RAR's ability to produce concise and semantically accurate answers.

Compared to baseline models like BERT, RoBERTa, and XLNet, which exhibit low EM scores (below 10%), IP-RAR excels in aligning retrieved sentences with precise answers.

Model name	Sentence			Answer
	P	R	F1	EM
TANDA	56.48	16.42	25.44	8.95
BERT	56.18	16.25	25.21	8.89
RoBERTa	57.70	19.06	28.65	9.40
XLNet	56.05	19.73	29.19	9.09
MultiCo	58.16	55.90	57.00	22.05
SELF-RAG	27.46	**82.84**	41.26	12.96
**IP-RAR(ours)**	**60.95**	68.35	**64.44**	**47.29**

Table 3. Comparison of preliminary sentence retrieval results between IP-RAR and commonly used methods on the MASH-QA dataset. The best results are highlighted in bold.

Analysis of the Recall for Multi-Level and Multi-Granularity Retrieval Strategy

Table 4 presents the recall (%) results of the Multi-Level and Multi-Granularity Retrieval Strategy and its ablation variants on the BioCDQA dataset. From the multi-level perspective, combining abstract-level and full-text-level retrieval ensures a more comprehensive capture of relevant information, covering knowledge sources from concise to detailed. Multi-level retrieval improves re-

Method	Hierarchical Retrieval			Multi-Granularity Retrieval	
	Abstract	Full-Text	Multi-Level	Value	Strategy Composition
Question-based	36.80	31.28	40.04	40.04	Only Question-based
Virtual Answer-based	34.79	27.63	39.62	44.06	+ Virtual Answer-based
Keyword-based	4.10	42.46	42.46	66.10	+ Keyword-based

Table 4. Recall (%) for Multi-Level and Multi-Granularity Retrieval Strategy.

call by 8.76% and 11.99% for Question-based Retrieval and Virtual Answer-based Retrieval, respectively, demonstrating that single-level retrieval alone is insufficient to achieve full coverage. By integrating results across levels, multi-level retrieval significantly enhances performance. However, for Keyword-based Retrieval, multi-level integration shows limited improvement, as keyword-based methods struggle to retrieve meaningful information at the abstract level. This indicates that keywords are less effective in abstracts, but their strong performance at the full-text level compensates for this limitation.

From the multi-granularity perspective, the strategy combines question-based, virtual answer-based, and keyword-based retrieval to leverage different representations of relevance. The ablation results demonstrate that each granularity contributes effectively to the final outcome. Question-based retrieval achieves a recall of 40.04%, while virtual answer-based retrieval further improves it to 44.06%. Keyword-based retrieval, particularly with synonym expansion, significantly boosts recall to 66.10%. This highlights that keyword-based retrieval complements the other granularities by capturing additional variations in linguistic expression. Importantly, each granularity contributes meaningfully, and their combined strengths play a crucial role in achieving the highest recall.

In summary, the integration of multi-level and multi-granularity strategies ensures the retrieval process captures relevant content comprehensively and effectively. The synergy between these dimensions is critical for achieving high recall, especially in large-scale datasets like BioCDQA, where maximizing coverage is essential for downstream applications.

Impact of IP-RAR Components on Performance

The ablation study presented in Table 5 provides insights into the contributions of various components in the IP-RAR framework under the DeepSeek-V3. By isolating individual components, the analysis demonstrates how each affects the overall system's ability to generate accurate and contextually appropriate answers.

In the ablation study, *w/o* Retrieval examines the system's performance when bypassing document retrieval entirely, relying solely on the DeepSeek-V3 to generate answers. In this configuration, the Precision, Recall, and F1 score metrics are not applicable, as no documents are retrieved. This setup leads to a significant performance drop, with a GPT-4 evaluation score of only 37.12%. These results underscore the vital role of the retrieval process in grounding the model's answers with relevant context. Without retrieval, the system lacks access to supporting information, resulting in vague or less accurate answers.

For *w/o* Integrated Reasoning-based Retrieval, the system bypasses the multi-level and multi-granularity retrieval strategy, directly retrieving a flat set of the top 50 text chunks using Contriever-MS MARCO and ranking the top 5 chunks during the progressive reasoning-based generation process. In this configuration, the Precision is 27.95%, Recall is 18.04%, and F1 score is 21.29%. This results in a GPT-4 evaluation score of 50.18%, reflecting the importance of the multi-level and multi-granularity retrieval strategy in refining the retrieval of highly relevant text chunks. While this alternative method retrieves some relevant documents, the overall recall remains too low to support comprehensive and accurate answer generation.

The *w/o* Progressive Reasoning-based Generation ablation removes the Progressive Reasoning-based Generation module and generates answers directly from the content retrieved by the Integrated reasoning-based Retrieval module. In this setup, the Precision drops to 16.13%, Recall increases to 32.81%, and F1 score decreases to 21.62%. Without the Progressive Reasoning-based Generation process, the system generates answers based on the top 5 chunks identified by the recall process, resulting in a GPT-4 evaluation score of 52.36%. Experimental results show that retrieving too much irrelevant information either introduces large amounts of invalid knowledge that disrupts the generation process or causes the context to exceed the token limit of the LLM, leading to the removal of potentially crucial information. The complete IP-RAR framework, with all components integrated, achieves the highest performance, with a GPT-4 evaluation score of 76.41%. Specifically, the Precision is 47.18%, Recall is 27.76%, and F1 score is 34.96%. This result underscores the importance of each component in ensuring the system's ability to deliver precise and contextually grounded answers. Removing any component results in a significant performance degradation, affirming that retrieval, integrated reasoning-based retrieval, and progressive reasoning-based generation are all essential for achieving optimal results in biomedical question answering.

Formulating Scientific Questions and Planning Research

Formulating scientific questions and designing research plans are critical steps in driving innovation and breakthroughs in biomedical research. A well-defined and challenging scientific question not only determines the direction of the study but also influences data collection, selection of experimental methods, and the interpretability of research outcomes. A well-structured research plan, in turn, optimizes resource allocation and enhances both feasibility and impact. By efficiently leveraging existing articles to identify research gaps and propose novel scientific hypotheses, researchers can significantly improve research efficiency. These hypotheses not only provide a solid theoretical foundation for subsequent experiments or clinical studies but also accelerate the understanding of disease mechanisms and the development of novel therapeutic strategies. Systematic analysis and integration of articles enable researchers to uncover unknown aspects of disease biology or limitations in current therapeutic approaches, thereby improving the rationality of hypotheses and the relevance of experimental designs. This process is crucial for drug development and the optimization of precision medicine strategies, ultimately expediting scientific discovery [33, 34].

Our framework facilitates the efficient extraction of key insights from existing article, generating scientific hypotheses and designing research plans. Figure 4a illustrates the systematic process of retrieval, integration, and reasoning, which ultimately generates evidence-based scientific questions to guide subsequent experimental designs. For instance, miR-375 may play distinct roles in the progression of different colorectal cancer subtypes, such as adenocarcinoma, squamous cell carcinoma, and small cell lung cancer. Its target gene, ITPKB, may be directly regulated by miR-375 and play a crucial role in cancer development. Based on this hypothesis, researchers can conduct targeted in vitro experiments,

System	Document Retrieval			Answer
	Prec. (%)	Rec. (%)	F1 (%)	GPT-4 Eval (%)
IP-RAR(ours)	47.18	27.76	34.96	76.41
w/o Progressive Reasoning-based Generation	16.13	32.81	21.62	52.36
w/o Integrated Reasoning-based Retrieval	27.95	18.04	21.29	50.18
w/o Retrieval	/	/	/	37.12

Table 5. Ablation study of the IP-RAR framework

such as luciferase reporter assays and Western blot analysis, to validate the regulation of ITPKB by miR-375. Additionally, in vivo studies using mouse models can effectively evaluate the impact of miR-375 on tumor growth and metastasis across different cancer subtypes. Further, large-scale high-throughput analyses of miR-375 expression patterns and its target genes in colorectal cancer patient cohorts, combined with correlations to clinical outcomes, can provide actionable research insights. This structured approach not only guides experimental design but also optimizes research workflows, enhancing both rigor and efficiency. By leveraging this literature-driven research methodology, researchers can accelerate scientific discoveries and improve the planning and execution of biomedical studies.

Drug Interaction Research in Clinical Decision Support

Research on drug interactions plays a crucial role in clinical decision support, ensuring treatment safety and efficacy, particularly in the context of polypharmacy and personalized medicine [35]. Many patients require multiple medications to manage comorbidities, making a deep understanding of drug interactions essential for clinicians to identify potential risks, such as enhanced side effects, altered drug metabolism, or reduced therapeutic effectiveness. Leveraging our approach, clinicians can more efficiently uncover synergistic effects that enhance therapeutic outcomes or identify antagonistic interactions that compromise efficacy [36]. This process not only aids in preventing adverse reactions but also supports the development of safer and more effective treatment plans, especially for elderly patients and those with multiple comorbid conditions.

For patients with coexisting lymphoma and colorectal cancer, robust clinical decision support is essential for optimizing treatment plans and reducing the risks associated with drug interactions. Figure 4b illustrates the system's retrieval and reasoning process, ultimately generating evidence-based answers. IP-RAR first performs pre-retrieval reasoning using a large language model, extracting keywords from the query and generating an initial response. It then retrieves relevant medical articles from databases to extract supporting evidence, including the facts that Cisplatin may induce resistance in lymphoma and that it has an antagonistic interaction with Cetuximab. The system then applies self-reflection to determine which text chunks are relevant to the question, followed by deep thinking to infer drug interactions. Finally, it recommends avoiding Cisplatin and suggests Carboplatin as an alternative therapy. This evidence-based reasoning approach assists clinicians in developing safer and more effective treatment plans, thereby enhancing patient safety and improving outcomes for individuals with complex, multi-disease conditions.

Discussion

To advance biomedical knowledge extraction and application, this paper proposes a comprehensive framework that integrates knowledge graphs with LLMs. By constructing the BioStrataKG from large-scale biomedical articles, the framework systematically uncovers multi-layered relationships among biomedical entities, such as genes, proteins, diseases, and drugs, as well as interactions between research papers in terms of methodologies, datasets, research directions, and citation relationships. To support cross-document reasoning and biomedical knowledge mining, we develop the BioCDQA dataset based on BioStrataKG, addressing the limitations of existing biomedical QA datasets in handling cross-document and high-level reasoning tasks. Furthermore, we introduce the IP-RAR framework, which combines Integrated Reasoning-based Retrieval with Progressive Reasoning-based Generation, enabling LLMs to efficiently retrieve, synthesize, and utilize multi-source evidence.

Experimental results demonstrate that IP-RAR significantly outperforms previous approaches in both retrieval efficiency and answer accuracy. In clinical applications, the framework assists physicians in rapidly identifying and synthesizing critical information from vast biomedical articles, facilitating the development of more precise personalized treatment plans. In research settings, it enables systematic analysis of cutting-edge advancements and identification of potential research gaps, accelerating research strategy formulation and decision-making. IP-RAR holds potential in various biomedical domains, including assisting in drug synergy/antagonism analysis, generating hypotheses for drug repurposing, and providing evidence synthesis for precision medicine, providing essential technical support for advancing biomedical research and clinical practice.

Despite its promising results, IP-RAR still faces challenges in handling highly complex multimodal data, dynamically evolving scientific knowledge, and human-AI interaction. Future work will explore multimodal information integration and agent-based interactions to further enhance the robustness and adaptability of the framework, ensuring more comprehensive support for biomedical knowledge mining and clinical applications. By continuously improving the dynamic maintenance of knowledge graphs and refining reasoning mechanisms, the proposed framework is expected to establish a more efficient and precise paradigm for biomedical knowledge discovery, fostering rapid advancements in scientific research and clinical decision-making.

Availability of Source Code and Requirements

Project name: BioCDQA

- Project homepage: https://github.com/yichun10/BioCDQA.git.
- Operating system(s): Linux (Ubuntu)
- Programming language: Shell Script (Bash) with Python 3.10.13
- Other requirements: Python 3.10.13 with GPU/CPU support, neo4j 5.13.0 (please see more requirements on Github repository).
- Licenses: MIT license
- Research Resource Identifier (#RRID): SCR_027068
- Computational Workflow DOI: https://doi.org/10.48546/WORKFLOWHUB.WORKFLOW.1744.1

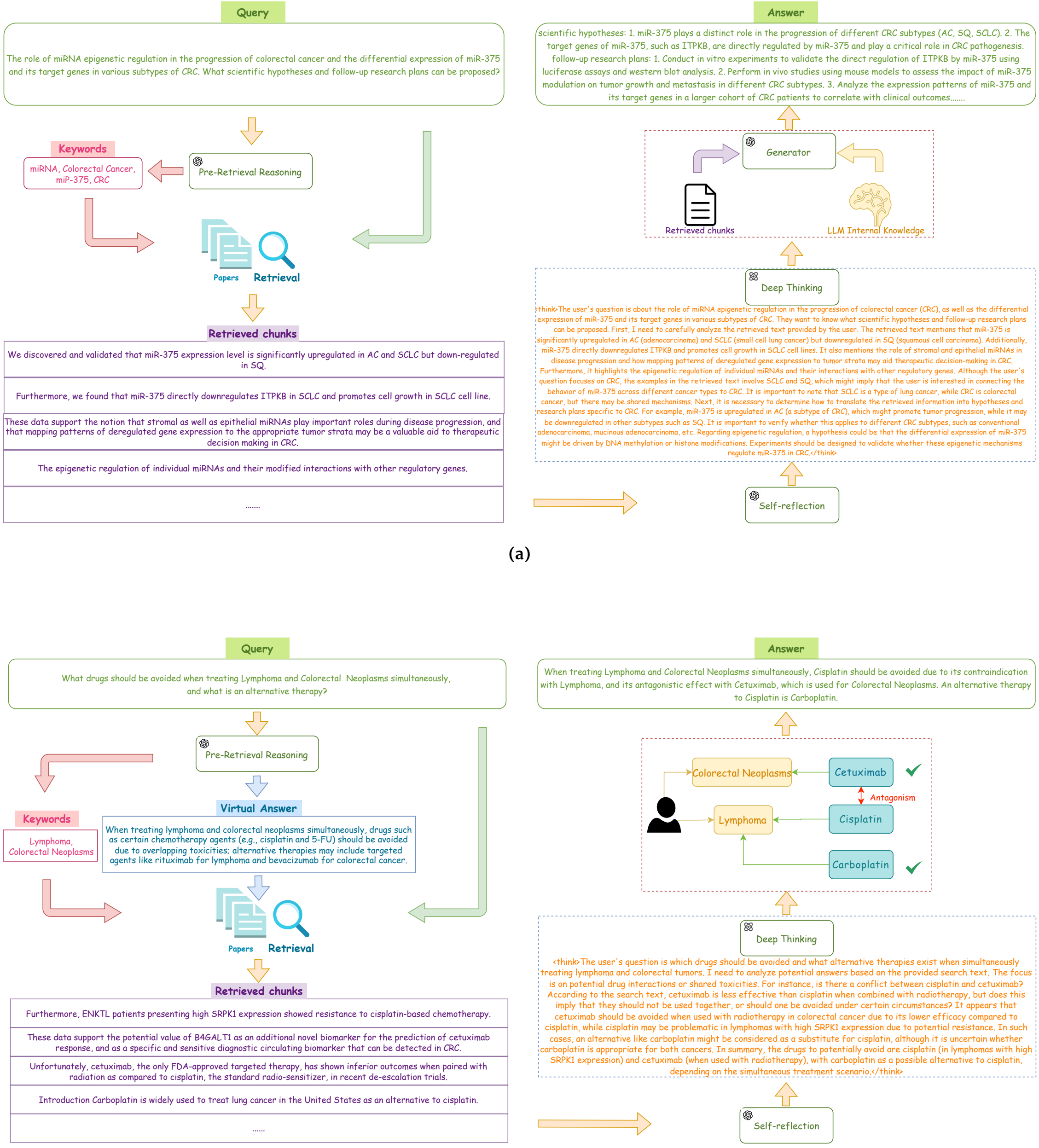

(b)

Figure 4. Examples of Applications in Biomedical Research and Clinical Decision Support. (a) An Example of Formulating Scientific Questions and Planning Research. (b) An Example of Drug Interaction Research for Clinical Decision Support.

Data Availability

The codes and datasets are openly available in the GigaScience repository, GigaDB [37].

Supplementary material

Supplementary material is available at Supplementary material.pdf.

Abbreviations

KG: knowledge graph; LLM: large language model; NLP: natural language processing; QA: question answering; RAG: Retrieval-Augmented Generation; IP-RAR: Integrated and Progressive Retrieval-Augmented Reasoning; GPT: generative pre-trained transformer.

Competing Interests

No competing interest is declared.

Funding

This work was supported in part by funds from the National Key R&D Program (No. 2022YFF1202101, 2023YFF1204701); the CAS Research Fund (No. XDB38050200); the Self-supporting Program of Guangzhou National Laboratory (No. SRPG22007); the Startup Program of Guangzhou National Laboratory (No. YW-YFYJ0101).

Authors' Contributions

Y.F. and L.Z. performed conceptualization and investigation; Y.F. performed data curation, formal analysis, methodology, and original draft writing; Y.L. and L.Z. performed resources and supervision; Y.F. , J.W., L.Z., R.H., and Y.L. reviewed and edited the manuscript.

References

1. Omiye JA, Gui H, Rezaei SJ, Zou J, Daneshjou R. Large language models in medicine: the potentials and pitfalls: a narrative review. Annals of internal medicine 2024;177(2):210–220.
2. Liu T, Zheng X, Chang B, Sui Z. Towards faithfulness in open domain table-to-text generation from an entity-centric view. In: Proceedings of the AAAI Conference on Artificial Intelligence, vol. 35; 2021. p. 13415–13423.
3. Comeau DC, Islamaj Doğan R, Ciccarese P, Cohen KB, Krallinger M, Leitner F, et al. BioC: a minimalist approach to interoperability for biomedical text processing. Database (Oxford) 2013;2013:bat064.
4. Doan S, Conway M, Phuong TM, Ohno-Machado L. Natural language processing in biomedicine: a unified system architecture overview. Clinical bioinformatics 2014;p. 275–294.
5. Cai L, Li J, Lv H, Liu W, Niu H, Wang Z. Integrating domain knowledge for biomedical text analysis into deep learning: A survey. Journal of Biomedical Informatics 2023;143:104418.
6. Chang D, Balažević I, Allen C, Chawla D, Brandt C, Taylor RA. Benchmark and best practices for biomedical knowledge graph embeddings. In: Proceedings of the conference. Association for Computational Linguistics. Meeting, vol. 2020; 2020. p. 167.
7. Li L, Wang P, Yan J, Wang Y, Li S, Jiang J, et al. Real-world data medical knowledge graph: construction and applications. Artificial intelligence in medicine 2020;103:101817.
8. Zheng S, Rao J, Song Y, Zhang J, Xiao X, Fang EF, et al. PharmKG: a dedicated knowledge graph benchmark for bomedical data mining. Briefings in bioinformatics 2021;22(4):bbaa344.
9. Wu X, Duan J, Pan Y, Li M. Medical knowledge graph: Data sources, construction, reasoning, and applications. Big Data Mining and Analytics 2023;6(2):201–217.
10. Zhu M, Ahuja A, Juan DC, Wei W, Reddy CK. Question answering with long multiple-span answers. In: Findings of the Association for Computational Linguistics: EMNLP 2020; 2020. p. 3840–3849.
11. Nentidis A, Katsimpras G, Krithara A, Lima López S, Farré-Maduell E, Gasco L, et al. Overview of bioasq 2023: The eleventh bioasq challenge on large-scale biomedical semantic indexing and question answering. In: International Conference of the Cross-Language Evaluation Forum for European Languages Springer; 2023. p. 227–250.
12. Welbl J, Stenetorp P, Riedel S. Constructing datasets for multi-hop reading comprehension across documents. Transactions of the Association for Computational Linguistics 2018;6:287–302.
13. Abacha AB, Mrabet Y, Sharp M, Goodwin TR, Shooshan SE, Demner-Fushman D. Bridging the gap between consumers' medication questions and trusted answers. In: MEDINFO 2019: Health and Wellbeing e-Networks for All IOS Press; 2019.p. 25–29.
14. Pal A, Umapathi LK, Sankarasubbu M. Medmcqa: A large-scale multi-subject multi-choice dataset for medical domain question answering. In: Conference on health, inference, and learning PMLR; 2022. p. 248–260.
15. Feng Y, Zhou L, Ma C, Zheng Y, He R, Li Y. Knowledge Graph-based Thought: a knowledge graph enhanced LLMs framework for pan-cancer question answering. GigaScience 2024;Accepted for publication, to appear.
16. Lewis P, Perez E, Piktus A, Petroni F, Karpukhin V, Goyal N, et al. Retrieval-augmented generation for knowledge-intensive nlp tasks. Advances in Neural Information Processing Systems 2020;33:9459–9474.
17. Jegal Y, Choi J, Lee J, Park KS, Lee S, Yoon J. Learning a Patent-Informed Biomedical Knowledge Graph Reveals Technological Potential of Drug Repositioning Candidates. arXiv preprint arXiv:230903227 2023;.
18. Izacard G, Caron M, Hosseini L, Riedel S, Bojanowski P, Joulin A, et al. Unsupervised Dense Information Retrieval with Contrastive Learning. Transactions on Machine Learning Research 2022;.
19. Asai A, Wu Z, Wang Y, Sil A, Hajishirzi H. Self-rag: Learning to retrieve, generate, and critique through self-reflection. ICLR 2024;.
20. Edge D, Trinh H, Cheng N, Bradley J, Chao A, Mody A, et al. From local to global: A graph rag approach to query-focused summarization. arXiv preprint arXiv:240416130 2024;.
21. Yan SQ, Gu JC, Zhu Y, Ling ZH. Corrective retrieval augmented generation. arXiv preprint arXiv:240115884 2024;.
22. Sarthi P, Abdullah S, Tuli A, Khanna S, Goldie A, Manning CD. Raptor: Recursive abstractive processing for tree-organized retrieval. In: The Twelfth International Conference on Learning Representations; 2024. .
23. Tang X, Zou A, Zhang Z, Li Z, Zhao Y, Zhang X, et al. MedAgents: Large Language Models as Collaborators for Zero-shot Medical Reasoning. In: Findings of the Association for Computational Linguistics ACL 2024; 2024. p. 599–621.
24. Jaech A, Kalai A, Lerer A, Richardson A, El-Kishky A, Low A, et al. Openai o1 system card. arXiv preprint arXiv:241216720 2024;.
25. Guo D, Yang D, Zhang H, Song J, Zhang R, Xu R, et al. Deepseek-r1: Incentivizing reasoning capability in llms via reinforcement learning. arXiv preprint arXiv:250112948 2025;.
26. OpenAI, GPT-4o mini: advancing cost-efficient intelligence; 2024. https://openai.com/index/gpt-4o-mini-advancing-cost-efficient-intelligence/.

27. Paruchuri V, Marker;. https://github.com/VikParuchuri/marker.
28. National Library of Medicine, Medical Subject Headings (MeSH); 2023. https://www.nlm.nih.gov/mesh/meshhome.html.
29. UniProt: the universal protein knowledgebase in 2023. Nucleic acids research 2023;51(D1):D523–D531.
30. Wang W, Wei F, Dong L, Bao H, Yang N, Zhou M. Minilm: Deep self-attention distillation for task-agnostic compression of pre-trained transformers. Advances in Neural Information Processing Systems 2020;33:5776–5788.
31. Liu A, Feng B, Xue B, Wang B, Wu B, Lu C, et al. Deepseek-v3 technical report. arXiv preprint arXiv:241219437 2024;.
32. Hurst A, Lerer A, Goucher AP, Perelman A, Ramesh A, Clark A, et al. Gpt-4o system card. arXiv preprint arXiv:241021276 2024;.
33. Rajpal DK, Qu XA, Freudenberg JM, Kumar VD. Mining emerging biomedical literature for understanding disease associations in drug discovery. Biomedical Literature Mining 2014;p. 171–206.
34. Mohs RC, Greig NH. Drug discovery and development: Role of basic biological research. Alzheimer's & Dementia: Translational Research & Clinical Interventions 2017;3(4):651–657.
35. Maher RL, Hanlon J, Hajjar ER. Clinical consequences of polypharmacy in elderly. Expert opinion on drug safety 2014;13(1):57–65.
36. Neuvonen PJ, Niemi M, Backman JT. Drug interactions with lipid-lowering drugs: mechanisms and clinical relevance. Clinical Pharmacology & Therapeutics 2006;80(6):565–581.
37. Feng Y, Wang J, He R, Zhou L, Li Y, Supporting data for "A Retrieval-Augmented Knowledge Mining Method with Deep Thinking LLMs for Biomedical Research and Clinical Support". GigaScience Database; 2025. https://doi.org/10.5524/102756.

Click here to access/download
Supplementary Material
Supporting Information.pdf

Click here to access/download
Supplementary Material
ourrag_4.svg
