# Supplementary material for: A retrieval-augmented knowledge mining method with deep thinking LLMs for biomedical research and clinical support
Source: Gigascience. 2025 Sep 17;14:giaf109. doi: 10.1093/gigascience/giaf109 (PMC12448786; doi:10.1093/gigascience/giaf109)
Supplement: giaf109_Authors_Response_To_Reviewer_Comments_Original_Submission [file giaf109_authors_response_to_reviewer_comments_original_submission.pdf]

Author's Response To Reviewer Comments

Revision Notes for GIGA-D-25-00234

The authors would like to thank editor and reviewers for their constructive comments and suggestions on our submission. We have addressed the comments and modified the manuscript accordingly. In this revised version, changes to our manuscript within the document have been highlighted by using blue colored text. Please refer to the file "Revision_highlight_giga_234.pdf" in the supplementary materials. Point-by-point responses to the editor and reviewers are listed below.

Response to EIC:
#Q1:The introductory assertions (e.g., supporting drug discovery) require supporting results or concrete examples.

Response: Thanks for your suggestion. We have performed a systematic revision of the language throughout the entire manuscript. In multiple sections, including the Abstract, Key Points, Introduction (lines 126 - lines 130), Figure Captions, and Discussion (lines 881 - lines 884), we have replaced overly broad verbs and nouns with more precise phrasing. For example, "drug repurposing" has been revised to "generating hypotheses for drug repurposing," and "accelerating scientific discovery" has been refined to "accelerating the hypothesis generation phase of scientific discovery." Through these changes, we have clearly defined the tool's role as a powerful hypothesis generation and decision support tool, rather than an end-to-end discovery platform.

#Q2:New tools must obtain RRIDs (via bio.tools/SciCrunch), and workflows require DOI registration on workflowhub.eu

Response: Thanks for your suggestion. We fully agree on the importance of these standard registrations for improving the discoverability and reproducibility of our work. We have already included our registered RRID (SCR_027068) in the "Availability of Source Code and Requirements" section of the manuscript. Furthermore, we have successfully registered our computational workflow on WorkflowHub and add the citable DOI (https://doi.org/10.48546/WORKFLOWHUB.WORKFLOW.1744.1) to this section as well (lines 909- lines 910).

Response to Reviewer #1:
#Q1: Paper collection method is not clear. PubMed has PubMed Central Open Access Subset FTP Bulk Download, which offers XML files in two distinct groups: commercial use and non-commercial use only. The paper collection needs to be clarified further why the PDFs were downloaded instead of XML files. Converting PDFs to Markdown format can introduce noises and the choice of PDFs is less appropriate as there were no usage of Figures or Tables. Also, how the PDFs were converted to markdown is unclear.

Response: Thanks for your suggestion.
(1)We initially considered using the XML files available from open-access repositories. However, we discovered that for a significant portion of the literature, the XML files contained only metadata and abstracts, with the full text being either missing or improperly formatted. As a key objective of our study is to perform full-text knowledge mining, relying solely on XMLs would have been insufficient. The PDF format was therefore essential to ensure we obtained the most complete information, including the detailed discourse within the body of the papers. To leverage the best of both, we adopted a hybrid approach: we preserved the structured metadata and abstracts from the XML files and integrated them with the complete full-text content extracted from the corresponding PDFs (lines 171 - lines 179).
(2)For the conversion, we utilized the marker toolkit, which is cited in our manuscript (references [25, 26]). We specifically chose marker because it excels at not only extracting text but also preserving the logical structure of the document (e.g., headings, lists, and paragraphs). We ensure that the revised Methods section clearly states the tool used(lines 179 - lines 181).

#Q2: No comparison of LLMs in constructing the KG and QA. Authors just used GPT4o-mini without

comparing the performance of this model with other models to support this model selection for both the knowledge graph construction and QA dataset generation. This is important to ensure the quality of the knowledge graph, BioStrataKG, and the dataset, BioCDQA.

Response: Thanks for your suggestion. We ensured quality through a multi-faceted approach. Our model selection was based on three key considerations. First, for practical reasons, GPT-4o mini offered the best balance of performance and cost-effectiveness for our large-scale task. Second, there was a strong technical fit: our "triplet extraction" is a highly structured task with predefined entity and relation types, which aligns perfectly with the strong instruction-following and contextual understanding capabilities of the GPT-4 series models. Third, to quantitatively validate this choice, we conducted a targeted evaluation on a 20-paper sample and found that GPT-4o mini achieved an extraction accuracy of 88.5%. This high accuracy is further supported by our process and product validation measures. Our construction process has built-in quality controls, such as a two-stage entity normalization process that requires alignment with authoritative databases (MeSH and UniProt) and secondary LLM confirmation, which inherently prevents a large number of errors. Finally, we conducted a functional validation via our downstream task. The successful creation of the BioCDQA dataset, which itself underwent rigorous manual curation (lines 458 - lines 475) (filtering from 5,000+ to 1,183 pairs), would have been infeasible if the underlying knowledge graph—the final product—were of poor quality.

#Q3: Limited novelty in the Progressive Reasoning-based Generation It is very similar to Self-Refine: Iterative Refinement with Self-Feedback (NeurIPS 2023). Especially the self-reflective evaluation is just another way of using self-refine.

Response: Thanks for your suggestion. While both approaches leverage a "self-reflection" loop, they are designed for fundamentally different purposes, which is where our novelty lies. Self-Refine's purpose is to iteratively improve a generated output using the model's internal knowledge—a task akin to "writing and editing." In contrast, our Progressive Reasoning-based Generation module was designed for the distinct purpose of selecting and distilling reliable evidence from a noisy pool of externally retrieved, multi-document sources, a task more akin to "intelligence analysis and verification." Therefore, our novelty is in adapting this reflective mechanism to solve a unique RAG challenge: preparing high-quality external evidence for complex downstream reasoning.

#Q4:Experiments should include other prompting methods such as Chain of Thought, In-context Learning (Few Shot), and medical agent based prompting methods such as MedAgent (MedAgents: Large Language Models as Collaborators for Zero-shot Medical Reasoning, ACL2024 Findings). This would ensure that IP-RAR is not only outperforming RAG approaches, but also other baseline methods.

Response: Thanks for your suggestion. Following your suggestion, we have added a crucial set of baseline experiments designed to directly contrast our RAG-based approach with non-RAG methods. Specifically, we evaluated the performance of several leading prompting strategies that rely solely on the model's internal, parametric knowledge, without the use of any retriever (Table 1) (lines 588 - lines 614). These include Chain of Thought , Few-Shot prompting, and MedAgents, for which we have now added a brief introduction in the Introduction section (lines 69 - lines 72). The purpose of this comparison is to clearly demonstrate that for our specific cross-document question-answering task, which requires precise, multi-source evidence, the Retrieval-Augmented Generation (RAG) paradigm is indispensable. By comparing the performance of our complete IP-RAR system against these non-RAG baselines, we can quantitatively show the significant performance gains afforded by external knowledge retrieval. This, in combination with our comparisons against other RAG methods (like CRAG and SELF-RAG), serves to prove that IP-RAR not only excels among RAG approaches but is also fundamentally superior to baseline methods that do not leverage an external knowledge corpus.

#Q5:How the Protein, Gene, and Biomarkers are ensured that they do not have redundant entities?

Response: Thanks for your suggestion. We describe our solution in the "Entity Normalization in Triplet Extraction" section. This normalization process is specifically designed to address the issue of redundancy: Phase 1 (Database-based Candidate Retrieval): For each entity extracted from the text , we use the all-

MiniLM-L6-v2 embedding model to retrieve the top 5 most similar standardized terms from authoritative databases (UniProt/MeSH). Phase 2 (LLM-based Contextual Adjudication): Subsequently, GPT-4o mini utilizes the context from the original text to select the most semantically appropriate term from these 5 candidates to serve as the final, standardized entity. This process effectively handles synonyms and abbreviations, thereby mapping different expressions to the same standard entity and preventing redundancy.

#Q6:The relationship has disease indication, but there is no node type for the indication such as symptom. Does this mean symptom is included in the disease node type?

Response: Thanks for your suggestion. In the current version of BioStrataKG, in order to simplify the ontology of the knowledge graph, we do consider "symptoms" as part of the concept of "disease" and classify them uniformly as "Disease" nodes.

#Q7: Including inference time for each method would support that IP-RAR's performance gain is not too costly.

Response: Thanks for your suggestion. To demonstrate that the performance advantages of our IP-RAR framework do not come at an excessive computational cost (lines 615 - lines 629), we analyzed its inference time and GPU memory usage in a direct comparison against a strong baseline, SELF-RAG, with all tests performed on a single NVIDIA A100 GPU. For a typical query, IP-RAR has an average inference time of 26.7 seconds and a peak GPU memory usage of only 3GB. In contrast, SELF-RAG takes 32.4 seconds and requires a peak of 78GB of GPU memory. Therefore, IP-RAR achieves its state-of-the-art accuracy while also being faster and vastly more computationally efficient than the baseline method, demonstrating the advanced and practical nature of its architecture.

Response to Reviewer #2:
#Q1:The paper asserts capabilities (e.g., drug repurposing) but lacks concrete examples or experimental validation. I would also suggest the introduction of keywords that help in understanding the scope and depth of the tool's application.

Response: Thanks for your suggestion. We have performed a systematic revision of the language throughout the entire manuscript. In multiple sections, including the Abstract, Key Points, Introduction (lines 126 - lines 130), Figure Captions, and Discussion (lines 881 - lines 884), we have replaced overly broad verbs and nouns with more precise phrasing. For example, "drug repurposing" has been revised to "generating hypotheses for drug repurposing," and "accelerating scientific discovery" has been refined to "accelerating the hypothesis generation phase of scientific discovery." Through these changes, we have clearly defined the tool's role as a powerful hypothesis generation and decision support tool, rather than an end-to-end discovery platform.

#Q2:Missing fundamental metrics (node degree distribution, edge cardinality) and graph measures (e.g., centrality)

Response: Thanks for your suggestion. We completely agree that introducing classic graph-theoretic metrics provides a deeper understanding of the knowledge graph's structural properties. As you pointed out, we had already provided initial statistics on the distribution of node and relationship types in Figure 2(b). Building on this, in our revised manuscript, we have supplemented this with the average degree and the node degree distribution (lines 205 - lines 207). We believe this enhanced analysis more comprehensively characterizes the topology and scale of BioStrataKG, highlighting the most influential nodes and the overall connectivity, thereby giving readers a clearer understanding of its structural features.

#Q3:No clarification on distinguishing genes/proteins (both use UniProt IDs) or mapping relationships to reference ontologies (e.g., RO)

Response: Thanks for your suggestion. Differentiating between genes and proteins from text is a classic challenge in biomedical natural language processing. Our method addresses this through a two-step

process. First, for each potential entity, the LLM analyzes its surrounding sentence context. If the text contains explicit identifiers, such as 'gene X' or 'expression of Y,' the model assigns the Genetype. In other cases, particularly when the text pertains to function, structure, or binding, the model assigns the Proteintype. Once the entity type is determined, the normalization process is performed strictly within that scope: entities marked as Gene are disambiguated against gene entries in UniProt, while those marked as Protein are disambiguated against protein entries. We fully agree that the RO is the recognized reference ontology for standardizing relationships in the biomedical domain. In the current version of our work, we defined a custom set of relationship types. This set was designed for pragmatic reasons, with the primary goal of effectively supporting our core downstream task: building the BioCDQA dataset. This approach facilitated more rapid development and allowed us to precisely capture the information required for our question-answering task.

#Q4:The large use of LLMs in the pipeline raises the concern of hallucinations. The authors should address how they mitigate this risk, especially since hallucinations can affect the accuracy of the entire pipeline, from query to final output. In particular, how are hallucinations handled at each stage of the process, from academic database query to knowledge graph & Q/A dataset generation?

Response: Thanks for your suggestion. We fully agree that mitigating the risk of hallucination in Large Language Models is critical for ensuring the accuracy of our entire pipeline. To this end, we have designed specific mitigation strategies for each key stage of our process:
(1)In the Knowledge Graph Construction Stage: We employ a strict entity normalization process. This process requires every extracted entity to be mapped to a corresponding official entry in authoritative databases (e.g., MeSH and UniProt). This fundamentally constrains the model from fabricating entities, ensuring the authenticity of the nodes in our knowledge graph.
(2)In the Dataset Construction Stage: We conducted rigorous manual verification for all machine-generated question-answer pairs. This process involved domain experts tracing back to the source literature to check the accuracy and faithfulness of each answer, thereby guaranteeing that every answer in the BioCDQA dataset is grounded in evidence.
(3)In the Question-Answering Stage: We have implemented a multi-layered hallucination suppression framework. First, the core principle of Retrieval-Augmented Generation (RAG) is to ground the LLM's response using retrieved text from real literature. Second, the Progressive Reasoning and Self-reflection mechanism within our framework further filters out irrelevant text chunks that could introduce noise, preventing the LLM from reasoning based on unrelated information. Finally, this evidence-based requirement for answers significantly suppresses any ungrounded generation.

#Q5:The paper mentions that the QA dataset is generated from a knowledge graph using an LLM-based procedure, and the EM metrics and the GPT scoring system are used to evaluate the performance of another LLM. There seems to be a risk of a circular evaluation loop, where the same technology is used to assess itself.

Response: Thanks for your suggestion. We acknowledge the potential bias in using an LLM to evaluate the output of another LLM. However, given the practical constraints of large-scale human annotation, employing a more capable, advanced LLM (like GPT-4) as an evaluator is a widely accepted and pragmatic solution in the current NLP landscape, especially for complex generation tasks with non-deterministic answers that require deep semantic understanding. To further mitigate bias, we intentionally used a heterogeneous model setup: our question-answering generation relies primarily on the DeepSeek series, while performance evaluation is conducted using OpenAI's GPT-4 series. This design effectively circumvents self-preference bias, preventing a model from receiving an unfairly high score due to stylistic similarities with the evaluator. Furthermore, to guarantee the absolute reliability of our benchmark dataset, we implemented an exceptionally rigorous manual curation protocol during its construction (lines 458 - lines 475). From an initial pool of over 5,000 machine-generated QA pairs, we filtered down to 1,183 high-quality entries. This process was independently conducted by two authors with biomedical backgrounds, who evaluated each pair against a comprehensive set of criteria. These criteria included factual correctness and faithfulness (ensuring every assertion in the answer is clearly supported by and traceable to the source literature, and is free of hallucinations), completeness (no omission of critical information), relevance and conciseness(the answer is focused and without redundant details), and the quality of the question itself (clear and unambiguous). For each QA pair, reviewers meticulously compared the question, the

generated answer, and the source text, and only those that met all standards were provisionally accepted. Following the independent reviews, the results were compared, and only QA pairs with consensus approval from both reviewers were retained for the final dataset.

#Q6:It is unclear the reason for which the authors have chosen Neo4j for their knowledge graph when the relationships are modeled using triples rather than properties. The properties of nodes and edges are not discussed in the paper, though the supplementary materials mention extracting node properties from texts. It would be helpful to explain how these properties are derived, and how issues like data duplication are prevented, especially when the same entity may be linked to different attributes across various texts. Additionally, the authors mention extracting properties from nodes but do not discuss any similar extraction for edges. Since relationships are central to the knowledge graph, it would be essential to include property extraction for edges to enhance the graph's semantic richness.

Response: Thanks for your suggestion. Our choice of Neo4j is based on its mature ecosystem, a powerful visualization interface, and the intuitive Cypher query language. Although our core data model is based on triplets, Cypher is more convenient for performing native graph operations such as multi-hop queries and pathfinding, which is highly valuable for exploratory analysis. In fact, every relationship (edge) in our knowledge graph includes a crucial property: the Evidence Source, which is the ID of the original paper that supports the relationship. This attribute is fundamental to ensuring that every relationship in our knowledge graph is traceable. We will clarify and emphasize this point in our revised manuscript (lines 246 - lines 250). To ensure the consistency of our knowledge graph, we designed a clear de-duplication strategy. For Nodes, we prevent node duplication through a rigorous Entity Normalization process, which maps different textual mentions to a single, canonical entity ID. For Relationships, we allow multiple edges of different types to exist between two nodes, but we de-duplicate any identical (node1, relation, node2) triplets to ensure their uniqueness.

#Q7:The authors do not clarify which vector database they are using. Is it ChromaDB? Moreover, the chunking strategy is not explained. How are large texts split into manageable pieces for processing?The use of the Max factor in the similarity formula implies that every chunk is compared with all other chunks. This approach is computationally expensive, especially as the number of articles increases. The authors should discuss the scalability of their method and propose potential optimizations for larger datasets.

Response: Thanks for your suggestion. Our retrieval pipeline does not utilize a dedicated vector database like ChromaDB; instead, its core is based on the Contriever model. The recommended implementation for Contriever involves pairing it with an efficient vector indexing library. In our implementation, we follow this best practice: the vector embeddings generated by Contriever are stored in an IVF+HNSW index built using the FAISS library. This indexing structure enables Approximate Nearest Neighbor (ANN) search, which significantly reduces the computational complexity of retrieval from linear time, O(N) (where N is the total number of text chunks), to logarithmic time, O(log N). This means that even as the number of articles in our corpus grows from tens of thousands to millions or more, the retrieval time will only increase very slowly. Therefore, our method is computationally efficient and fully capable of scaling to much larger datasets without requiring architectural changes to the core retrieval component. For the full text of the papers, we employed the Recursive Character Text Splitter strategy (lines 321 - lines 329). We set the chunk size to 500 characters with a 100-character overlap between consecutive chunks. This strategy was chosen because it effectively attempts to keep sentences whole when splitting large documents, thereby maximally preserving local context and reducing information loss from improper segmentation. We add these crucial technical details to the Methods section of our revised manuscript to improve its clarity.

#Q8:The related work section does not adequately introduce Contriever and CRAG, two methods that appear relevant to the problem. The authors should provide more detailed comparisons to these systems. Additionally, bioinfo-0 and dmiip3 are mentioned but not introduced.

Response: Thanks for your suggestion. We have expanded the descriptions of Contriever (lines 54 - lines 57) and CRAG (lines 63 - lines 67) in the Introduction section, detailing their core mechanisms. Regarding bioinfo-o and dmiip3, we have now added a brief introduction in the Results section (lines 634 - lines 643) to establish a clear benchmark for our performance comparison and to provide the necessary

background information for the reader.

#Q9:Citation [1] is not pertinent and should be revised.

Response: Thanks for your suggestion. We have replaced this citation with a review article that is more directly relevant to our work on the applications of LLMs in the biomedical domain.

#Q10:Figure 1: It is not clear why a query would be placed in the biological data sources box. A query should typically be used to extract or search data, not act as data itself. Moreover, there is a lack of clarity on the transition between steps b and c in the figure. Figure 3b is introduced without proper context. It should be clearly explained in the text as an example of the relevant concept, as it seems to play a key role in the argument but is not sufficiently introduced.

Response: Thanks for your suggestion. To resolve the logical issue of placing a "Query" within "Knowledge Sources," we have restructured the figure (Figure 1) to show the "Knowledge Source" (i.e., papers) separately from the "Query," which now acts as a direct input to the processing pipeline. Furthermore, to clarify the transition from the process to its applications, we have replaced the single generic arrow with more specific arrows, explicitly linking the "Knowledge Graph" and the "Answer" outputs to their respective downstream applications in part (C). To improve the figure's clarity, we will revise the caption for Figure 3 in our revised manuscript (the caption for Figure 3). The updated caption will provide a more detailed explanation of the 'Relevant Text Chunks' section in Figure 3(B), making it clear that these are evidence fragments retrieved from the source literature by our various retrieval strategies in part (A) (e.g., 'Question Retrieval,' 'Keyword matching,' and 'Virtual Answer Retrieval'), each marked with its source.

#Q11:In Figure 4, the interaction with the user is unclear. The figure suggests that the system involves expert interaction, but this is never elaborated on in the text.

Response: Thanks for your suggestion. Figure 4 illustrates an automated workflow designed to provide decision support for domain experts. The system automatically generates a complete, evidence-based analytical report, upon which an expert can base their subsequent decisions. The key point of potential confusion lies with the human icon in Figure 4(b). We wish to clarify that this icon represents a "patient" in a clinical case study (specifically, a patient with colorectal neoplasms and lymphoma), and not a "user" who is interacting with our system. We will clarify this in the figure caption of our revised manuscript to eliminate any ambiguity.

#Q12:The authors use @prefix ex: as a namespace in the turtle representation of the graph. This is unclear to me.

Response: Thanks for your suggestion. We completely agree that using as a namespace was unclear. This was indeed a placeholder in our data export scripts. Following your suggestion, we have now corrected our data files. We have updated this placeholder to a unique and persistent namespace that points to our project's GitHub repository (e.g., https://github.com/yichun10/BioCDQA/schema#). Furthermore, we have added a detailed SCHEMA.md file to the GitHub repository (https://github.com/yichun10/BioCDQA/blob/main/SCHEMA.md) to clearly define and describe all the nodes, relationships, and properties in our knowledge graph.

#Q13:Additionally, providing an online endpoint for Neo4j rather than just a dump would improve the system's accessibility for future research.

Response: Thanks for your suggestion. Regarding the suggestion to provide an online Neo4j endpoint for live queries, we agree this is a valuable idea for enhancing data accessibility. However, considering the long-term maintenance costs and resources required, we have prioritized providing the data in two more stable and reproducible formats at this stage. We offer: The complete Neo4j database dump file, which allows other researchers to perfectly replicate our graph environment; An open RDF-formatted version of the graph, which has been uploaded to the GigaDB FTP server in accordance with the journal's data sharing standards. We believe these two options will fully support the reuse and exploration of our data by the

research community.

#Q14:In Figure 2, is the Academic Database PubMed?

Response: Thanks for your suggestion. Yes, the papers used in our study were primarily sourced from PubMed. This is described in the "Paper Collection" section of our manuscript.

Editorial Manager®

Cookie Preference Center

We use cookies which are necessary to make our site work. We may also use additional cookies to analyze, improve and personalize our content and your digital experience. For more information, see our Cookie Policy.

You may choose not to allow some types of cookies. However, blocking some types may impact your experience of our site and the services we are able to offer. See the different category headings below to find out more or change your settings.

Allow all

Manage Consent Preferences

Strictly Necessary Cookies

Always active

These cookies are necessary for the website to function and cannot be switched off in our systems. They are usually only set in response to actions made by you which amount to a request for services, such as setting your privacy preferences, logging in or filling in forms. You can set your browser to block or alert you about these cookies, but some parts of the site will not then work.

Functional Cookies

These cookies enable the website to provide enhanced functionality and personalisation. They may be set by us or by third party providers whose services we

have added to our pages. If you do not allow these cookies then some or all of these services may not function properly.

Performance Cookies

These cookies allow us to count visits and traffic sources so we can measure and improve the performance of our site. They help us to know which pages are the most and least popular and see how visitors move around the site.

Targeting Cookies

These cookies may be set through our site by our advertising partners. They may be used by those companies to build a profile of your interests and show you relevant adverts on other sites. If you do not allow these cookies, you will experience less targeted advertising.

Cookie List

Clear

Apply Cancel

Consent Leg.Interest

Confirm my choices

Powered by onetrust
