# Supplementary material for: A retrieval-augmented knowledge mining method with deep thinking LLMs for biomedical research and clinical support
Source: Gigascience. 2025 Sep 17;14:giaf109. doi: 10.1093/gigascience/giaf109 (PMC12448786; doi:10.1093/gigascience/giaf109)
Supplement: giaf109_Authors_Response_To_Reviewer_Comments_Revision_1 [file giaf109_authors_response_to_reviewer_comments_revision_1.pdf]

# Author's Response To Reviewer Comments

Revision Notes for GIGA-D-25-00234R1

The authors would like to thank editor and reviewers for their constructive comments and suggestions on our submission. We have addressed the comments. Point-by-point responses to the editor and reviewers are listed below.

Response to Reviewer #1:
#Q1: Comparison of LLMs in constructing the KG and QA.
Authors provided extraction accuracy of GPT4o-mini on 20 paper samples. This is too little and does not justify how they just used GPT4o-mini without demonstrating the performance of this model over other models to support this model selection for both the knowledge graph construction and QA dataset generation. This is important to ensure the quality of the knowledge graph, BioStrataKG, and the dataset, BioCDQA.

# Response: Thanks for your suggestion. We fully understand the reviewer's concern regarding the rigor of our model selection and will hereby elaborate on our comprehensive considerations. Our choice of GPT-4o mini was a pragmatic and strategic decision. In the initial phase of the project, we found through random sampling that its performance on our task was satisfactory. More importantly, our "triple extraction" is a highly customized and structured task with specific entity and relation types. While many fine-tuned models demonstrate superior performance on certain specific tasks, their generalization capabilities are often compromised as a result. This is because their extraction schemas are rigidly embedded within their training data, making it difficult for them to flexibly adapt to our novel and unique extraction rules without costly retraining. In contrast, GPT-4o mini's strong instruction-following and zero-shot learning capabilities allow it to directly understand and execute the complex rules we define in the prompt, achieving an optimal balance between performance, cost, and efficiency for processing our large-scale corpus of over 15,000 full-text papers. Finally, we did not rely solely on the model itself for quality assurance; instead, we designed a rigorous, multi-stage quality control process. The core of this process is our two-stage entity normalization method, which mandates that every entity extracted by the LLM must be successfully mapped to an official entry in an authoritative database; otherwise, it is discarded. This procedure not only significantly suppresses the potential for model hallucination through validation against an external gold standard but also ensures the accuracy of the knowledge graph nodes. The successful construction and rigorous manual review of our downstream BioCDQA dataset retroactively demonstrate the high reliability of the upstream knowledge graph, thereby validating the correctness of our technical approach of selecting GPT-4o mini supplemented with strict quality control.

#Q2: No manual validation on subgraph to ensure the quality of the LLM generated KG.
Unlike the QA dataset, KG did not go through manual validation, which leaves this KG questionable in reliability and weakens the one of the major contribution of the work. This has not been addressed in the revision.

# Response: Thanks for your suggestion. We strongly agree that validation is crucial for a knowledge graph. Directly performing a complete manual validation on a large-scale knowledge graph with nearly 100,000 nodes and 300,000 relationships is indeed impractical, so we adopted a systematic and scalable quality assurance strategy. First, at the structural level, we ensured the authenticity of all nodes through our entity normalization process, where each node corresponds to an ID in an authoritative database. Concurrently, we annotated every relationship with a clear Evidence Source property, making it directly traceable to the original paper, which guarantees the graph's transparency and verifiability. Second, at the functional level, the rigorous manual curation we conducted on the BioCDQA dataset served as a large-scale, task-driven validation process for its underlying subgraphs. While reviewing the 1,183 question-answer pairs, domain experts repeatedly verified the underlying knowledge paths required to support the answers. Any erroneous relationships on these paths would have been discovered, as they would lead to inaccurate answers. This process therefore serves as a powerful functional validation for the overall reliability of our knowledge graph.

#Q3: Limited novelty in the Progressive Reasoning-based Generation
Although authors have claimed that their method is fundamentally different. I was more interested in how the self-reflective evaluation is different from Self-Refine. To me, the proposed method is still a combination of Self-Refine and RAG.

# Response: Thanks for your suggestion. We acknowledge that "self-reflection" is a shared mechanism in both approaches, but we would like to emphasize that their application purposes and contexts are entirely different. The goal of Self-Refine is to look inward, leveraging the model's internal knowledge to optimize the text it has generated—a process of self-editing. In contrast, our method looks outward, using the reflection mechanism to solve a core challenge within the RAG framework: how to evaluate and filter the large volume of noisy evidence retrieved from external sources. Our self-reflective evaluation is not about revising the answer, but about quantitatively scoring the reliability of external evidence and filtering this knowledge to address the real-world challenges of complex, multi-document reasoning.

Response to Reviewer #2:
#Q1: While it is now clear why the authors chose Neo4j for their knowledge graph representation, the properties assigned to the nodes are still not discussed. Including this information would improve overall clarity and reproducibility.

# Response: Thanks for your suggestion. We agree with your point regarding the lack of description for node properties and have addressed it. To comprehensively and clearly present the structure of BioStrataKG, we have created a detailed SCHEMA.md file that systematically defines all node labels, relationship types, and the specific properties for each type of node. This file is available in our GitHub repository: https://github.com/yichun10/BioCDQA/blob/main/SCHEMA.md

Editorial Manager®

## Cookie Preference Center

We use cookies which are necessary to make our site work. We may also use additional cookies to analyze, improve and personalize our content and your digital experience. For more information, see our Cookie Policy.

You may choose not to allow some types of cookies. However, blocking some types may impact your experience of our site and the services we are able to offer. See the different category headings below to find out more or change your settings.

Allow all
**Manage Consent Preferences**

**Strictly Necessary Cookies**

**Always active**

These cookies are necessary for the website to function and cannot be switched off in our systems. They are usually only set in response to actions made by you which amount to a request for services, such as setting your privacy preferences, logging in or filling in forms. You can set your browser to block or alert you about these cookies, but some parts of the site will not then work.

**Functional Cookies**

These cookies enable the website to provide enhanced functionality and personalisation. They may be set by us or by third party providers whose services we have added to our pages. If you do not allow these cookies then some or all of these services may not function properly.

**Performance Cookies**

These cookies allow us to count visits and traffic sources so we can measure and improve the performance of our site. They help us to know which pages are the most and least popular and see how visitors move around the site.

**Targeting Cookies**

These cookies may be set through our site by our advertising partners. They may be used by those companies to build a profile of your interests and show you relevant adverts on other sites. If you do not allow these cookies, you will experience less targeted advertising.

**Cookie List**

Clear

Apply Cancel

Consent Leg.Interest

Confirm my choices

Powered by onetrus
