# Supplementary material for: A retrieval-augmented knowledge mining method with deep thinking LLMs for biomedical research and clinical support
Source: Gigascience. 2025 Sep 17;14:giaf109. doi: 10.1093/gigascience/giaf109 (PMC12448786; doi:10.1093/gigascience/giaf109)
Supplement: giaf109_Authors_Response_To_Reviewer_Comments_Revision_2 [file giaf109_authors_response_to_reviewer_comments_revision_2.pdf]

# Author's Response To Reviewer Comments

Response to EIC:

#Q1: Corresponding Author Clarification

# Response: To clarify, Dr. Lu Zhou and Dr. Yixue Li have been clearly indicated as the corresponding authors at all stages of manuscript preparation and submission. I was listed as the corresponding author in the submission system purely for administrative convenience and to facilitate communication during the submission process. In the manuscript itself, from the initial submission to the final accepted version, Dr. Zhou and Dr. Li were consistently marked as the corresponding authors.

According to Gigascience's guidelines, corresponding authors must be readily available throughout the submission and peer-review process to respond to editorial queries promptly. They are also expected to address post-publication critiques and comply with requests for additional data or information to address any issues that may arise after publication. If an author's seniority makes them too busy to fulfill this role effectively, it is important to ensure that the designated corresponding authors can respond promptly. Busy researchers often face delays due to travel, institutional responsibilities, or high email volumes, which can significantly hinder the publication process. This is why I acted as the corresponding author in the submission system—to streamline communication and ensure the timely handling of relevant matters.

#Q2: Data Availability Statement

# Response: We have updated the Data Availability section with the following statement: "The codes and datasets are openly available in the GigaScience repository, GigaDB [REF#], and have been formally cited in the References list.

#Q3: Citations Update

# Response: All arXiv references with published versions have been updated to their official journal citations. References not yet published in peer-reviewed journals or conferences remain in their preprint versions.

em Editorial Manager®

## Cookie Preference Center

We use cookies which are necessary to make our site work. We may also use additional cookies to analyze, improve and personalize our content and your digital experience. For more information, see our Cookie Policy.

You may choose not to allow some types of cookies. However, blocking some types may

impact your experience of our site and the services we are able to offer. See the different category headings below to find out more or change your settings.

Allow all

## Manage Consent Preferences

#### Strictly Necessary Cookies

**Always active**

These cookies are necessary for the website to function and cannot be switched off in our systems. They are usually only set in response to actions made by you which amount to a request for services, such as setting your privacy preferences, logging in or filling in forms. You can set your browser to block or alert you about these cookies, but some parts of the site will not then work.

#### Functional Cookies

These cookies enable the website to provide enhanced functionality and personalisation. They may be set by us or by third party providers whose services we have added to our pages. If you do not allow these cookies then some or all of these services may not function properly.

#### Performance Cookies

These cookies allow us to count visits and traffic sources so we can measure and improve the performance of our site. They help us to know which pages are the most and least popular and see how visitors move around the site.

#### Targeting Cookies

These cookies may be set through our site by our advertising partners. They may be used by those companies to build a profile of your interests and show you relevant adverts on other sites. If you do not allow these cookies, you will experience less targeted advertising.

### Cookie List

Clear

Apply Cancel

Consent Leg.Interest

Confirm my choices

Powered by onetrus
