# Supplementary material for: A retrieval-augmented knowledge mining method with deep thinking LLMs for biomedical research and clinical support
Source: Gigascience. 2025 Sep 17;14:giaf109. doi: 10.1093/gigascience/giaf109 (PMC12448786; doi:10.1093/gigascience/giaf109)
Supplement: giaf109_Reviewer_1_Report_Original_Submission — Yunsoo Kim -- 7/10/2025 [file giaf109_reviewer_1_report_original_submission.pdf]

Reviewer Report

Title: A Retrieval-Augmented Knowledge Mining Method with Deep Thinking LLMs for Biomedical Research and Clinical Support

Version: Original Submission **Date:** 7/10/2025

Reviewer name: Yunsoo Kim

Reviewer Comments to Author:

Summary
The work presents 3 major contributions: a new knowledge graph (BioStrataKG), dataset for reasoning capability (BioCDQA), and novel prompting method (IP-RAR).
IP-RAR outperforms other baseline methods such as SELF-RAG on the BioCDQA dataset as well as other datasets, BioASQ and MASH-QA.
Strengths
1. Comprehensive experiments for RAG variant prompting methods.
2. Comprehensive experiments for IP-RAR on generalizability across multiple datasets.
3. Releasing the manually validated QA dataset for medical reasoning capabilities of LLMs.
Weaknesses
1. Paper collection method is not clear.
PubMed has PubMed Central Open Access Subset FTP Bulk Download, which offers XML files in two distinct groups: commercial use and non-commercial use only. The paper collection needs to be clarified further why the PDFs were downloaded instead of XML files. Converting PDFs to Markdown format can introduce noises and the choice of PDFs is less appropriate as there were no usage of Figures or Tables. Also, how the PDFs were converted to markdown is unclear.
2. No comparison of LLMs in constructing the KG and QA.
Authors just used GPT4o-mini without comparing the performance of this model with other models to support this model selection for both the knowledge graph construction and QA dataset generation. This is important to ensure the quality of the knowledge graph, BioStrataKG, and the dataset, BioCDQA.
3. No manual validation on subgraph to ensure the quality of the LLM generated KG.
Unlike the QA dataset, KG did not go through manual validation, which leaves this KG questionable in reliability and weakens the one of the major contribution of the work.
4. Limited novelty in the Progressive Reasoning-based Generation
It is very similar to Self-Refine: Iterative Refinement with Self-Feedback (NeurIPS 2023). Especially the self-reflective evaluation is just another way of using self-refine.
5. Experiments should include other prompting methods such as Chain of Thought, In-context Learning (Few Shot), and medical agent based prompting methods such as MedAgent (MedAgents: Large Language Models as Collaborators for Zero-shot Medical Reasoning, ACL2024 Findings). This would ensure that IP-RAR is not only outperforming RAG approaches, but also other baseline methods.
Suggestions and Questions
1. How the Protein, Gene, and Biomarkers are ensured that they do not have redundant entities?

2. The relationship has disease indication, but there is no node type for the indication such as symptom. Does this mean symptom is included in the disease node type?
3. Including inference time for each method would support that IP-RAR's performance gain is not too costly.

Methods

Are the methods appropriate to the aims of the study, are they well described, and are necessary controls included? Choose an item.

Conclusions

Are the conclusions adequately supported by the data shown? Choose an item.

Reporting Standards

Does the manuscript adhere to the journal's guidelines on [minimum standards of reporting?]() Choose an item.

Choose an item.

Statistics

Are you able to assess all statistics in the manuscript, including the appropriateness of statistical tests used? Choose an item.

Quality of Written English

Please indicate the quality of language in the manuscript: Choose an item.

Declaration of Competing Interests

Please complete a declaration of competing interests, considering the following questions:

- Have you in the past five years received reimbursements, fees, funding, or salary from an organisation that may in any way gain or lose financially from the publication of this manuscript, either now or in the future?
- Do you hold any stocks or shares in an organisation that may in any way gain or lose financially from the publication of this manuscript, either now or in the future?
- Do you hold or are you currently applying for any patents relating to the content of the manuscript?
- Have you received reimbursements, fees, funding, or salary from an organization that holds or has applied for patents relating to the content of the manuscript?
- Do you have any other financial competing interests?
- Do you have any non-financial competing interests in relation to this paper?

If you can answer no to all of the above, write 'I declare that I have no competing interests' below. If your reply is yes to any, please give details below.

I declare that I have no competing interests

I agree to the open peer review policy of the journal. I understand that my name will be included on my report to the authors and, if the manuscript is accepted for publication, my named report including any attachments I upload will be posted on the website along with the authors' responses. I agree for my report to be made available under an Open Access Creative Commons CC-BY license (http://creativecommons.org/licenses/by/4.0/). I understand that any comments which I do not wish to be included in my named report can be included as confidential comments to the editors, which will not be published.

Choose an item.

To further support our reviewers, we have joined with Publons, where you can gain additional credit to further highlight your hard work (see: https://publons.com/journal/530/gigascience). On publication of this paper, your review will be automatically added to Publons, you can then choose whether or not to claim your Publons credit. I understand this statement.

Yes Choose an item.
