# Supplementary material for: A retrieval-augmented knowledge mining method with deep thinking LLMs for biomedical research and clinical support
Source: Gigascience. 2025 Sep 17;14:giaf109. doi: 10.1093/gigascience/giaf109 (PMC12448786; doi:10.1093/gigascience/giaf109)
Supplement: giaf109_Reviewer_1_Report_Revision_1 — Yunsoo Kim -- 8/15/2025 [file giaf109_reviewer_1_report_revision_1.pdf]

Reviewer Report

Title: A Retrieval-Augmented Knowledge Mining Method with Deep Thinking LLMs for Biomedical Research and Clinical Support

Version: Revision 1 **Date:** 8/15/2025

Reviewer name: Yunsoo Kim

Reviewer Comments to Author:

Summary
The work presents 3 major contributions: a new knowledge graph (BioStrataKG), dataset for reasoning capability (BioCDQA), and novel prompting method (IP-RAR).
IP-RAR outperforms other baseline methods such as SELF-RAG on the BioCDQA dataset as well as other datasets, BioASQ and MASH-QA.
The authors addressed some of the concerns (paper collection method and comparison with other prompting methods) raised in the last review.
Strengths
1. Comprehensive experiments for RAG variant prompting methods as well as other prompting methods such as Chain of Thought.
2. Comprehensive experiments for IP-RAR on generalizability across multiple datasets.
3. Releasing the manually validated QA dataset for medical reasoning capabilities of LLMs.
4. IP-RAR computational efficiency is better than SELF-RAG.
Weaknesses
1. Comparison of LLMs in constructing the KG and QA.
Authors provided extraction accuracy of GPT4o-mini on 20 paper samples. This is too little and does not justify how they just used GPT4o-mini without demonstrating the performance of this model over other models to support this model selection for both the knowledge graph construction and QA dataset generation. This is important to ensure the quality of the knowledge graph, BioStrataKG, and the dataset, BioCDQA.
2. No manual validation on subgraph to ensure the quality of the LLM generated KG.
Unlike the QA dataset, KG did not go through manual validation, which leaves this KG questionable in reliability and weakens the one of the major contribution of the work. This has not been addressed in the revision.
3. Limited novelty in the Progressive Reasoning-based Generation
Although authors have claimed that their method is fundamentally different. I was more interested in how the self-reflective evaluation is different from Self-Refine. To me, the proposed method is still a combination of Self-Refine and RAG.

Methods

Are the methods appropriate to the aims of the study, are they well described, and are necessary controls included? Choose an item.

Conclusions

Are the conclusions adequately supported by the data shown? Choose an item.

Reporting Standards

Does the manuscript adhere to the journal's guidelines on [minimum standards of reporting?] Choose an item.

Choose an item.

Statistics

Are you able to assess all statistics in the manuscript, including the appropriateness of statistical tests used? Choose an item.

Quality of Written English

Please indicate the quality of language in the manuscript: Choose an item.

Declaration of Competing Interests

Please complete a declaration of competing interests, considering the following questions:

- Have you in the past five years received reimbursements, fees, funding, or salary from an organisation that may in any way gain or lose financially from the publication of this manuscript, either now or in the future?
- Do you hold any stocks or shares in an organisation that may in any way gain or lose financially from the publication of this manuscript, either now or in the future?
- Do you hold or are you currently applying for any patents relating to the content of the manuscript?
- Have you received reimbursements, fees, funding, or salary from an organization that holds or has applied for patents relating to the content of the manuscript?
- Do you have any other financial competing interests?
- Do you have any non-financial competing interests in relation to this paper?

If you can answer no to all of the above, write 'I declare that I have no competing interests' below. If your reply is yes to any, please give details below.

I declare that I have no competing interests

I agree to the open peer review policy of the journal. I understand that my name will be included on my report to the authors and, if the manuscript is accepted for publication, my named report including any attachments I upload will be posted on the website along with the authors' responses. I agree for my

report to be made available under an Open Access Creative Commons CC-BY license (http://creativecommons.org/licenses/by/4.0/). I understand that any comments which I do not wish to be included in my named report can be included as confidential comments to the editors, which will not be published.

Choose an item.

To further support our reviewers, we have joined with Publons, where you can gain additional credit to further highlight your hard work (see: https://publons.com/journal/530/gigascience). On publication of this paper, your review will be automatically added to Publons, you can then choose whether or not to claim your Publons credit. I understand this statement.

Yes Choose an item.
