# Supplementary material for: A retrieval-augmented knowledge mining method with deep thinking LLMs for biomedical research and clinical support
Source: Gigascience. 2025 Sep 17;14:giaf109. doi: 10.1093/gigascience/giaf109 (PMC12448786; doi:10.1093/gigascience/giaf109)
Supplement: giaf109_Reviewer_2_Report_Original_Submission — Marco Mesiti -- 7/30/2025 [file giaf109_reviewer_2_report_original_submission.pdf]

Reviewer Report

Title: A Retrieval-Augmented Knowledge Mining Method with Deep Thinking LLMs for Biomedical Research and Clinical Support

Version: Original Submission **Date:** 7/30/2025

Reviewer name: Marco Mesiti

Reviewer Comments to Author:

In this article, the authors propose a novel approach that utilizes LLMs to construct biomedical knowledge graphs from articles and build a question-answering dataset based on them. The authors claim that the system can support rational design and drug discovery processes.
The paper is easy to follow and well-organized. However, several critical issues need to be addressed before the work can be considered for publication.
Major comments:
The main issue is that the paper repeatedly claims that the proposed tool can facilitate e.g. drug repurposing and synergy detection. However, the paper does not show that the tool is actually able to achieve these objectives. The claims made in the introduction do not seem to be substantiated by results or concrete examples within the study. I would also suggest the introduction of keywords that help in understanding the scope and depth of the tool's application.
While the resulting knowledge graph has a reasonable size, even simple metrics like node degree distribution are not present. Specific cardinalities per node and edge type, as well as the distribution of nodes, would provide valuable information about the graph's scale and coverage. Classic graph metrics (e.g., average degree, centrality measures) would further enhance the reader's understanding of the knowledge graph's characteristics.
The paper does not clarify how the authors differentiate genes from proteins if both are identified using UniProt IDs. Additionally, the relationships in the knowledge graph do not seem to be mapped onto a recognized reference ontology (e.g., RO).
The large use of LLMs in the pipeline raises the concern of hallucinations. The authors should address how they mitigate this risk, especially since hallucinations can affect the accuracy of the entire pipeline, from query to final output. In particular, how are hallucinations handled at each stage of the process, from academic database query to knowledge graph & Q/A dataset generation?
The paper mentions that the QA dataset is generated from a knowledge graph using an LLM-based procedure, and the EM metrics and the GPT scoring system are used to evaluate the performance of another LLM. There seems to be a risk of a circular evaluation loop, where the same technology is used to assess itself. Furthermore, the manual checking process is mentioned but not clearly explained. How is the manual review conducted, and what criteria are used to ensure that it is reliable?
It is unclear the reason for which the authors have chosen Neo4j for their knowledge graph when the relationships are modeled using triples rather than properties. The properties of nodes and edges are not discussed in the paper, though the supplementary materials mention extracting node properties from texts. It would be helpful to explain how these properties are derived, and how issues like data

duplication are prevented, especially when the same entity may be linked to different attributes across various texts. Additionally, the authors mention extracting properties from nodes but do not discuss any similar extraction for edges. Since relationships are central to the knowledge graph, it would be essential to include property extraction for edges to enhance the graph's semantic richness.
The authors do not clarify which vector database they are using. Is it ChromaDB? Moreover, the chunking strategy is not explained. How are large texts split into manageable pieces for processing?
The use of the Max factor in the similarity formula implies that every chunk is compared with all other chunks. This approach is computationally expensive, especially as the number of articles increases. The authors should discuss the scalability of their method and propose potential optimizations for larger datasets.
The related work section does not adequately introduce Contriever and CRAG, two methods that appear relevant to the problem. The authors should provide more detailed comparisons to these systems. Additionally, bioinfo-0 and dmiip3 are mentioned but not introduced.
Detailed comments:
Citation [1] is not pertinent and should be revised.
Figure 1: It is not clear why a query would be placed in the biological data sources box. A query should typically be used to extract or search data, not act as data itself. Moreover, there is a lack of clarity on the transition between steps b and c in the figure.
Figure 3b is introduced without proper context. It should be clearly explained in the text as an example of the relevant concept, as it seems to play a key role in the argument but is not sufficiently introduced.
In Figure 4, the interaction with the user is unclear. The figure suggests that the system involves expert interaction, but this is never elaborated on in the text.
Minor comments:
The authors use @prefix ex: <http://example.org/> as a namespace in the turtle representation of the graph. This is unclear to me. Additionally, providing an online endpoint for Neo4j rather than just a dump would improve the system's accessibility for future research.
In Figure 2, is the Academic Database PubMed?

Methods

Are the methods appropriate to the aims of the study, are they well described, and are necessary controls included? Choose an item.

Conclusions

Are the conclusions adequately supported by the data shown? Choose an item.

Reporting Standards

Does the manuscript adhere to the journal's guidelines on [minimum standards of reporting?]() Choose an item.

Choose an item.

Statistics

Are you able to assess all statistics in the manuscript, including the appropriateness of statistical tests used? Choose an item.

Quality of Written English

Please indicate the quality of language in the manuscript: Choose an item.

Declaration of Competing Interests

Please complete a declaration of competing interests, considering the following questions:

- Have you in the past five years received reimbursements, fees, funding, or salary from an organisation that may in any way gain or lose financially from the publication of this manuscript, either now or in the future?
- Do you hold any stocks or shares in an organisation that may in any way gain or lose financially from the publication of this manuscript, either now or in the future?
- Do you hold or are you currently applying for any patents relating to the content of the manuscript?
- Have you received reimbursements, fees, funding, or salary from an organization that holds or has applied for patents relating to the content of the manuscript?
- Do you have any other financial competing interests?
- Do you have any non-financial competing interests in relation to this paper?

If you can answer no to all of the above, write 'I declare that I have no competing interests' below. If your reply is yes to any, please give details below.

I declare that I have no competing interests

I agree to the open peer review policy of the journal. I understand that my name will be included on my report to the authors and, if the manuscript is accepted for publication, my named report including any attachments I upload will be posted on the website along with the authors' responses. I agree for my report to be made available under an Open Access Creative Commons CC-BY license (http://creativecommons.org/licenses/by/4.0/). I understand that any comments which I do not wish to be included in my named report can be included as confidential comments to the editors, which will not be published.

Choose an item.

To further support our reviewers, we have joined with Publons, where you can gain additional credit to further highlight your hard work (see: https://publons.com/journal/530/gigascience). On publication of this paper, your review will be automatically added to Publons, you can then choose whether or not to claim your Publons credit. I understand this statement.

Yes Choose an item.
